# Effect of a parenting and nutrition education programme on development and growth of children using a social safety-net platform in urban Bangladesh: a cluster randomized controlled trial

**DOI:** 10.1016/j.lansea.2024.100388

**Published:** 2024-03-19

**Authors:** Sheikh Jamal Hossain, Syed Moshfiqur Rahman, Jane Fisher, Anisur Rahman, Fahmida Tofail, Jena Derakhshani Hamadani

**Affiliations:** a Global Health and Migration Unit, Department of Women's and Children's Health, Uppsala University, Sweden; b International Centre for Diarrhoeal Disease Research Bangladesh (icddr,b), Mohakhali, Dhaka, 1212, Bangladesh; c Global and Women's Health, School of Public Health and Preventive Medicine, Monash University, Melbourne, VIC, 3004, Australia

**Keywords:** Children, Cognitive, Language, Development, Social safety-net, Bangladesh

## Abstract

**Background:**

Although sustainable development goals mandate for quality early childhood development (ECD) interventions for children <8 years, little occurs for children <3 years, especially in urban settings in low-and-middle-income countries (LMICs). Our primary objective was to measure the effect of an ECD-focused parenting and nutrition education on children's development through home visits using a social safety net platform of urban Bangladesh.

**Methods:**

A cluster randomized controlled trial was conducted with mothers of children aged 6–16 months in 20 clusters across the Rangpur city, Bangladesh. The intervention group received fortnightly ECD-focused parenting and nutrition education at homes by local Community Health Workers (CHWs) for one year. Bayley-III was used to measure children's cognitive, language and motor development. Data were analyzed using intention to treat. ClinicalTrials.gov Identifier: NCT03753646.

**Findings:**

Out of 599 mother-child dyads, 56.6% mothers were aged ≤ 25 years old. After one year, the intervened children had higher cognitive [Effect size Cohen's d; 0.42 SD (95% CI: 0.58–0.25)], language (0.38 SD, 95% CI: 0.55–0.22) and motor (0.17 SD, 95% CI: 0.01–0.34) development. In the intervention group, mothers experienced less violence [Odds ratio; 0.6 (95% CI: 0.4–1.0)] and fathers engaged more (0.23 SD, CI: 0.39–0.06) in ECD activities with their children compared to the comparison group. Total home stimulation and mothers' knowledge on child care were also improved in the intervention. But the children's growth was not improved.

**Interpretation:**

This ECD programme improves the development of children of young mothers in urban settings using a social safety-net platform. The evidence may help in increasing ECD coverage in urban areas in LMICs.

**Funding:**

10.13039/501100004828Grand Challenges Canada, Saving Brains Programme Grant Number: SB-1810-20176.

**Research in context**

**Evidence before this study**

We reviewed the literature exploring if there was any intervention that used a social safety net platform in urban areas in deprived settings to see the effect of ECD-focused parenting and nutrition education on children's developmental outcomes. We searched the PUBMED databases with the search terms “early childhood development”, “social safety net”, “cognitive function”, “language development”, “motor development”, “psychosocial stimulation” “parenting”, and “integrated child development interventions” to identify randomized controlled trials of ECD intervention programmes on 30 April, 2023. No language or time restrictions were applied to this search. We considered the main outcomes as children's cognitive, language and motor development. The literature review yielded a few randomized controlled trials that successfully integrated parenting into health and nutrition platforms and conditional cash transfer programmes. We did not find any study that used an unconditional cash transfer programme as a social safety net programme in urban deprived areas in low- and middle-income countries.

**Added value of this study**

Evidence from the literature suggests that early childhood development intervention for children less than three years is of global importance. Early childhood stimulation programme coverage for poor people is also a big challenge for low- and middle-income countries. The delay in early childhood development can be prevented or recovered by early childhood stimulation interventions integrated into health, nutrition, or social safety-net (conditional/unconditional cash transfer) programmes, especially those that directly target disadvantaged children and their families. Our intervention was effective for the development of economically disadvantaged Bangladeshi urban children using social a safety net programme (unconditional cash transfer), especially where mothers were young.

**Implications of all the available evidence**

Our findings support the potential for an ECD focused-parenting programme through home visits using a national social safety net programme (unconditional cash transfer programme) that can be used effectively to improve urban deprived children's cognitive, language and motor development in low-income settings such as urban Bangladesh where the primary health care system is complex and patchy. Since the intervention reduced violence against mothers, improved their knowledge on childcare and fathers' involvement, the effects may be long lasting.

## Introduction

The first five years of a child's life are very important since during this time about 90% of the brain architecture develops.^1^ During this early age, the children are exposed to several risk factors such as poverty, lack of nutrition and unstimulating home environments that can impede their healthy development.^2^ Developmental delay is a common problem in low- and middle-income countries where an estimated 249.4 million children fail to reach their developmental potential.^3^ When risk factors are present at an early age, their negative impacts can be observed throughout the life course of children not only in terms of their behaviour, schooling, income and social well-being but also at the level of national economy.^4^^,^^5^

Early intervention for children has been shown to improve their lives, both during the school-age years and afterward, including their employment prospects and level of income.^5^, ^6^, ^7^ In addition, these investments often help those most in need.^8^ Parenting programmes through home visits are a widely accepted method to promote healthy child development, where trained community health workers (CHWs) visit mother-child dyads in their homes to coach on specific interventions, such as parenting or father's involvement for proper care of mother and child.^9^ Home-visiting programmes are a promising approach ensuring children in LMICs receive the nurturing care they need to reach their developmental potential.^8^ One of the most important and ground-breaking randomized trials of an early childhood development (ECD), Reach Up and Learn focusing on home-visiting programmes in Jamaica, demonstrated that malnourished children whose mothers received home visits had better cognitive and motor development in childhood,^10^ higher IQ and language skills in adolescence,^11^ as well as less depression and violence, and higher earnings, in adulthood.^5^^,^^7^ After that, small-scale ECDs focusing on home visiting programmes were successfully operationalized in LMICs,^6^^,^^12^^,^^13^ however, success in scaling-up evidence-based ECD programmes to regional and national levels has been limited.^14^ One reason for this gap is the absolute complexity of delivering relationship-based services to large numbers of families across diverse settings.^15^^,^^16^

In Bangladesh, growing unplanned urbanization is a problem especially with regards to poor maternal and child health, development and education.^17^ While parents with adequate incomes are able to provide their children with plenty of books and play materials, enriching activities and high-quality childcare; parents with insufficient incomes find it more difficult to provide their children with experiences that support optimal child development.^18^ Father engagement may not be optimal in this scenario.^19^ In addition, mothers from low income communities are more prone to depressive symptoms and other mental health problems, which can undermine positive parenting.^20^^,^^21^ Many studies documented that the development of the children in deprived urban settings lags behind the development of children who are better off.^22^^,^^23^

The Government of Bangladesh (GoB) aims to achieve SDG target 4.2, where the country is committed to provide quality ECD interventions for all children aged 0–8 years but the intervention has so far rarely included children between the ages of 0–3 years. The GoB has developed a comprehensive Early Childhood Development Policy (Bangladesh ECCD policy 2013) and related strategy which presently requires evidence for younger children's development in order to make it scalable, especially in urban settings.

We previously adapted and tested the Reach Up and Learn intervention in different populations (e.g. malnourished, maltreated, poor, iron-deficient anemic) in rural settings in Bangladesh and found that the intervention produces had moderate and strong effect sizes.^24^, ^25^, ^26^, ^27^ One of the projects showed medium-term effects on children's development.^6^ However, we only evaluated the intervention in two small urban projects, where the children were severely malnourished and were identified through an urban health facility.^12^^,^^28^ Therefore, the evidence from urban Bangladesh is limited. A previous study of a similar intervention conducted in urban Brazil used readily available health workers and newly hired ones in two different arms of the study and found that neither of the intervention arm improved the children's cognitive, language and motor development.^29^ We have also identified that the intervention did not work in rural Madagascar.^30^ In light of the above, we planned to deliver a scalable parenting programme using a government social safety-net platform (lactating allowance/unconditional cash for the urban poor mothers) through CHWs home-visits in urban areas. We used a national social safety-net programme of the Ministry of Women and Children's Affairs (MoWCA), GoB. This ministry is responsible for coordinating child development activities based on the Comprehensive Early Childhood Development Policy of the GoB (Bangladesh ECCD Policy 2013).

There are several benefits to using a social safety-net platforms (lactating allowance/any form of unconditional cash) in deprived urban settings in Bangladesh to deliver parenting programme. Our previous models of ECD-focused parenting intervention integrated into the primary health care system were tested in rural areas of Bangladesh and we needed to produce evidence to show if this intervention would also work in deprived urban setting of the country. The urban primary health care system in Bangladesh is fragmented and patchy. It is run by private sector mostly, which are unable to provide services to most poor urban people as most of these services require fees. Unfortunately, most poor people are usually out of primary health care system and the poor are often in need of home delivered health and ECD services. The lactating allowance or unconditional cash programme is one of the biggest social safety-net programme for poor urban lactating mothers in Bangladesh. Using this functional platform, GoB will be able to support the development of children in most vulnerable urban families in the country with limited costs involved. Moreover, the lactating allowance or unconditional cash itself may be helpful for children's growth and development by potentially mitigating negative impacts of early life shocks.^31^ Unconditional cash may also improve consumption of animal foods by both mothers and children and reduce household food insecurity.^32^ The parenting intervention among the mothers who received lactating allowance or any form of unconditional cash may provide a number of additional benefits for both the mothers and children,^33^ although we were unable to measure the additive or independent effect of cash in the current intervention.

We primarily hypothesised that the ECD-focused parenting and nutrition education intervention combined with unconditional cash will improve children's cognitive, language and motor development of the children compared with only unconditional cash in deprived settings of urban Bangladesh. Our secondary hypotheses were that the combined intervention will improve children's growth and their stimulation environment, the mother's knowledge on child rearing and care practices, the father's involvement in stimulatory activities and reduce household violence against mothers.

## Methods

### Study design

We implemented a cluster randomized controlled trial in 20 clusters with two arms of i) intervention: receiving parenting and nutrition education through home visits + unconditional cash, and ii) comparison: receiving only unconditional cash. We recruited the mothers (intervention and comparison) from the urban lactating allowance programme (social safety-net for mother and child) of the GoB in Rangpur City Corporation. Poor urban lactating mothers were receiving unconditional cash of 800 BDT (9.4 USD, Ref: World Bank 2020) per month through this social safety-net programme. The clusters of this study were *Wards* (lowest geographical division) of the City Corporation. The clusters were similar in terms of their culture and customs. We enrolled participants in this trial from December, 2019 to March, 2020.

### Participants and sampling

To identify the participants, we collected the list of mothers in the urban lactating allowance programme in Rangpur City Corporation from the Department of Women, MoWCA, GoB. This City Corporation is characterized by high rates of poverty and stunting. The majority of the population are unskilled informal workers. No other ECD programmes or interventions were ongoing in these areas at the time of this study.

We enrolled mothers who had a child aged 6–16 months and who were not expected to leave the study site for longer than 2 months. The mothers/caregivers had to be able to understand the contents of the informed consent document and provide consent. We excluded the known congenital anomalies, developmental disorders or severe developmental delays, not possible to test the child due to physical or behavioural problems and children of multiple birth e.g. twin, triplets. We enrolled younger mothers first where there were more than 30 mothers in the same cluster. Due to the long distance between the groups, interaction between the mothers of the intervention and control groups was unlikely. Nevertheless, we tried to minimize the spillover effect by excluding mother child dyads located on the border of intervention and control clusters.

### Sample size

In our context, previous studies of psychosocial stimulation alone achieved an average effect size of 0.37 SD on children's cognitive development.^34^ In this study, we had additional cash as an intervention that might also positively affect children's development. But to be on the safe side, we assumed an effect size of 0.35 SD, 80% power, with 95% confidence intervals, 1.58 cluster design effect and 25% drop-out rate. The sample size was calculated to be 600 with 30 children in each of the 20 clusters.

### Randomization and blinding

The clusters were randomly allocated in a 1:1 ratio to one of the two trial arms: i) ECD-focused parenting and nutrition education through home visits + unconditional cash, and ii) comparison: only unconditional cash. Randomization was done by a non-study statistician using a computer-generated randomization list. The home visits for the intervention did not give us a chance to blind the implementers. Nevertheless, we took several steps to keep the data collectors as masked to group status as possible. First, the data collection team worked independently from programme implementers and was not in contact during training or interviewing. Second, the children's assessment was done in a suitable place other than the children's houses (school, community centre or other people's houses), so that the assessor could not take note of any play materials provided by the project staff and thus deduce the child's group status (intervention or comparison). Nevertheless, the mothers of the intervention group might have talked about the intervention during post intervention assessments and the assessors might have been unblinded. Additionally, the data analysis was blinded. All mother/child dyads had been recruited before randomization.

### Procedure

We used a structured curriculum based on the Reach Up and Learn curriculum and incorporated nutrition education for mothers and children relevant to the Bangladeshi context. The unadjusted Reach Up and Learn curriculum is available online.^35^ It has already been adapted and used previously in Bangladesh.^24^^,^^25^ We recruited CHWs and trained them for ten days on the curriculum to be able to deliver fortnightly sessions of 40–60 min at the household of the participants for one year. The total number of sessions planned was 25 for each mother. Training focused on the content of the intervention and effective communication skills as well as encouraging the involvement of the fathers and other caregivers alongside primary caregivers (mothers). Parents/caregivers were requested to practice between the sessions. The CHWs used a number of age-specific play materials such as plastic shakers, puzzles, balls, dolls, rings with string, and picture books that were made of locally available low-cost materials. The components of each session were i) building rapport with mothers/caregivers, ii) recap of the previous session iii) demonstrating the play materials and activities of the current session to the mother/caregivers and child, iv) delivering developmental messages, v) providing nutrition education and vi) leaving the play materials at the home so that the caregivers could use them for play with the child for the following two weeks. The intervention was based on Bandura's social-cognitive learning theory.^36^

We engaged only local female CHWs, whilst considering the future scalability of the study. All trained CHWs continued their sessions throughout the project period. Each mother and child dyad was attended to by a single CHW at all sessions. The average age of the CHWs was 25 (range 20–28 years) years and their minimum educational level was twelfth grades. They were permanent residents in the intervention area.

One female quality officer ensured the quality of the intervention through random visits to the CHWs. The quality officers offered a monthly short refresher training for first three months and short discussions with all CHWs when they found any deviation from the quality of the sessions throughout the intervention period. The CHWs took play materials with them to conduct the sessions while two male field organizers ensured the timely availability and dispatch of the play materials to the CHWs.

### Outcomes and measurements

We used the Bayley scales of infant and toddler development, third edition (Bayley III) to collect data on children's cognitive, language and motor development as primary outcomes. Our team validated the tool in Bangla and used it in a number of studies in Bangladesh.^24^ It is an individually administered instrument that measures the developmental level of children between 16 days and 42 months. We measured the children's growth using WHO standards,^37^ the mother's knowledge on child care practices (pre-specified in the protocol), the children's stimulation environment, the father's involvement in stimulatory activities with the children and household violence against the mothers (not pre-specified in the protocol) as secondary outcomes. Domestic violence was defined if the husband or any household member verbally threatened the mother or physically or sexually abused her. Mothers reported on domestic violence experienced in their households during the period of the previous one month. Family Care Indicators (FCI), a validated tool in Bangla was used to measure the children's stimulation environment and the father's involvement to child stimulation.^38^ The father's involvement with the child was measured using five activities (yes/no), i) reading a book, ii) telling stories, iii) playing together with a toy, iv) singing songs or rhymes and v) drawing or counting, performed over the previous three days preceding the interview.

We measured the families’ food security status using the Household Food Insecurity Access Scale (HFIAS)^39^ and we collected further socioeconomic information on these families.

Six female assessors (university-graduates with a degree in social science/psychology) conducted the Bayley-III test, anthropometry and other measurement s at enrollment and one-year after the intervention. Two assessors worked together in the field: one assessor conducted the Bayley test to assess cognition, language and motor development while another assessor interviewed the mother for other information. The assessors received one month of training on Bayley-III test administration and the performance of other necessary measurements.

### Statistical analysis

We analyzed the data using Stata version 12.1. A wealth index, as a measure of socio-economic status, was created by principal component analysis (PCA) from the information collected on household assets. We converted the children's height and weight to height-for-age and weight-for-age Z score following WHO standards.^37^ Continuous and categorical values were presented as mean (SD) and percentage, respectively, to be able to compare the socioeconomic information and outcomes by groups.

We fitted cluster-adjusted similar multivariate linear regressions for continuous outcomes and logistic regression for the binary outcomes of violence against the mothers. All models were adjusted for the age and sex of the children, the assessors' effect, respective baseline level of outcome and the background information that showed an imbalance between the groups. We used intention-to-treat analyses. We calculated effect sizes using Cohen's d for significant outcomes.

Post hoc mediation analysis were done using a structural equation model to see if the mothers' child care knowledge and quality of the home environment acted as mediators.^26^ We also investigated if there were differences in the outcomes of the intervention based on the mother's age and education and the children's sex. We conducted additional adjusted regression models controlling the interaction term: mothers' age∗intervention and in another model children's sex∗intervention and again in another model mothers' education and intervention. All post hoc analyses are presented in the Supplementary Tables.

### Ethical assurance

The project was approved by the Institutional Review Board of icddr,b (Protocol Number-18035) and the caregivers provided written informed consent.

### Role of the funding source

The funder of the study had no role in study design, data collection, data analysis, data interpretation, or writing of the report. All authors had full access to all the data in the study and had final responsibility for the decision to submit for publication.

## Results

**Fig. 1:**
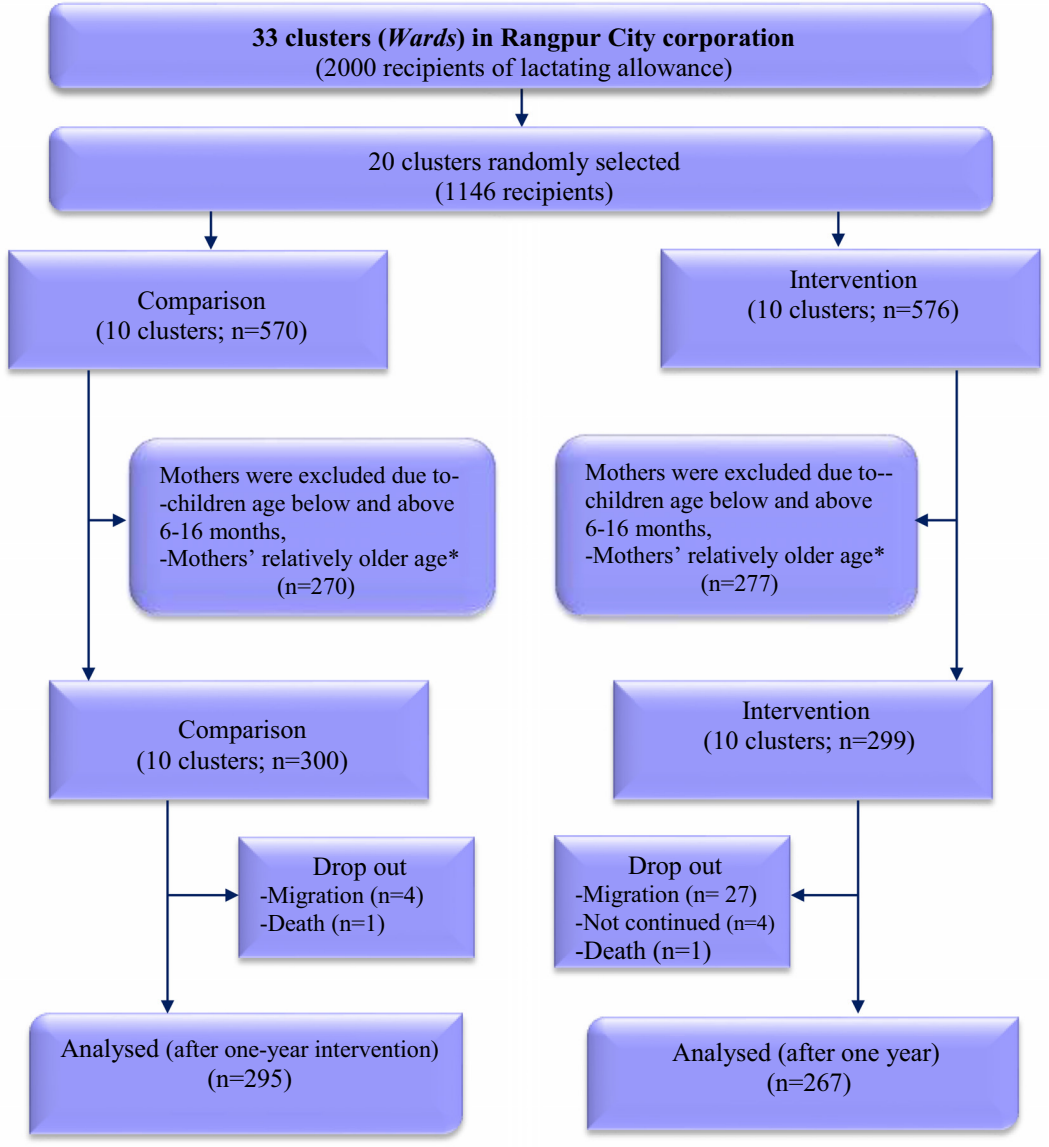
**Enrollment and follow-up of the participants**. Note: ∗First 30 younger mothers were enrolled in the cluster where there were more than 30 mothers from the list of mothers provided by Ministry of Women and Children's Affairs, Government of Bangladesh.

We enrolled a total of 599 mother-child dyads (intervention = 299, control = 300). After one-year of intervention, the attrition rate was 6.5% (n = 37), out of which 32 were in the intervention (10.70%) and 5 (1.66%) in the control groups (Fig. 1). Most attrition was due to families’ migration of families to other areas for livelihood. One child died due to drowning and another following diarrhoea episode. The background information between children lost and those tested at post-intervention were not statistically different (Table S1).

Prior to the beginning of the assessments, interobserver reliability was tested between each assessor and the trainer on 8–10 tests per assessor. Inter-observer reliabilities were acceptable for all measures. The agreement was more than 90% for Bayley (cognitive, language and motor development) scores and anthropometry measurements. Intraclass correlation coefficients (ICC) were >0.98 on Bayley composite scores. The questionnaires used for mothers had good internal consistency with Cronbach's α: 0.81 at baseline and 0.72 at post-intervention (Table S2).

Table 1 presents the background characteristics of the intervention and comparison groups at enrollment. The groups were well-balanced at enrollment apart from the mothers of the intervention group who experienced more domestic violence and had a higher wealth index compared to the mothers in the comparison group. Among the children, 303 (50.60%) were girls and the mean (SD) age of the children was 18.27 (4.02) months at enrollment. The mother's mean (SD) age was 25.62 (5.07 SD). In the study, 56.6% of the mothers were aged ≤ 25 years old (not presented in a table). Overall, the mothers had a mean (SD) of 7.70 (3.51) years of education.

**Table 1:** Background characteristics and outcomes at enrollment by groups.

Characteristics	Intervention (N = 299) Mean (SD)/n (%)	Comparison (N = 300) Mean (SD)/n (%)
Mothers' age	25.6 (4.8)	25.7 (5.4)
Mothers' education		
0–4 years	60 (20.1)	63 (21.0)
5–9 years	165 (55.2)	166 (55.3)
10≥ years	74 (24.7)	71 (23.7)
Mothers' BMI	22.7 (3.9)	23.1 (4.5)
Children's age in months	11.44 (3.1)	11.28 (3.2)
Children's sex (Female) n (%)	144 (48.2)	160 (53.3)
Crowding Index	3.0 (1.9)	2.8 (1.7)
Wealth Index n (%)		
Lowest	106 (35.4)	135 (45.0)
Middle	58 (19.4)	65 (21.7)
Upper	135 (45.2)	100 (33.3)
Food security status of the families n (%)		
Secure	68 (22.7)	55 (18.3)
Mild secure	18 (6.0)	23 (7.7)
Moderate secure	109 (36.5)	103 (34.3)
Insecure	104 (34.8)	119 (39.7)
**Outcomes**		
Children's cognitive development	94.49 (11.1)	93.5 (10.9)
Children's language development	92.1 (13.0)	90.7 (12.6)
Children's motor development	93.4 (15.2)	94.1 (14.7)
Length-for-age z score (LAZ)	−1.1 (1.2)	−1.1 (1.1)
Weight for age z score (WAZ)	−0.6 (1.0)	−0.7 (1.0)
Weight for Length age z score (WLZ)	−0.03 (1.0)	−0.1 (1.03)
Violence against mother (Yes) n (%)	157 (52.5)	132 (44.0)
Fathers' engagement for child care	0.4 (0.9)	0.4 (0.8)
Mothers' knowledge on child care	25.3 (6.2)	25.4 (7.0)
Total home stimulation environment	5.8 (3.2)	5.5 (3.3)

Values are mean (SD) unless otherwise stated. Child cognition, language and motor scores were measured using the Bayley Scales of Infant and Toddler Development-version III. Knowledge of child rearing practices was measured with a structured questionnaire used in previous studies (20 questions, potential range of scores: 0–60). Fathers' engagement was scored from five activities (yes = 1/no = 0): reading a book, telling story, playing, singing song or rhymes and drawing or counting to child. Total home stimulation environment was assessed using Family Care Indicators (24 questions, potential range of scores: 0–24).

After one year of intervention, the children in the intervention group had higher cognitive [Adjusted regression coefficient β (95% CI): 2.7 (1.3–4.1), Effect size Cohen's d; 0.42 SD (95% CI: 0.58–0.25)], language [β: 2.4 (95% CI: 0.4–4.4) (Effect size; 0.38 SD, 95% CI: 0.55–0.22)] and motor [β: 1.5 (95% CI: 0.1–2.8) (Effect size; 0.17 SD (95% CI: 0.01–0.34)] development (Table 2).

**Table 2:** Effect of intervention on primary and secondary outcomes.

Outcomes	Regression coefficient B (95% CI) n = 562	Effect size (95% CI) n = 562	P-value
Primary outcomes			
Children's cognitive development	2.7 (1.3–4.1)	0.4 (0.3–0.6)	0.001
Children's language development	2.4 (0.4–4.4)	0.4 (0.2–0.6)	0.02
Children's motor development	1.5 (0.1–2.8)	0.2 (0.01–0.3)	0.04
Secondary outcomes			
Father's engagement for child care	0.3 (0.03–0.5)	0.2 (0.1–0.4)	0.03
Violence against mother (Odds ratio)^$^	0.6 (0.4–1.0)	–	0.03
Total home stimulation	2.6 (2.1–3.1)	0.8 (1.0–0.6)	<001
Mother's knowledge on child care	3.8 (2.5–5.1)	0.6 (0.8–0.5)	<001
Height-for-age z score (HAZ)	0.1 (−0.1 to 0.2)	−0.2 (−0.3 to 0.01)	0.25
Weight for age z score (WAZ)	−0.1 (−0.2 to −0.03)	−0.01 (−0.2 to −0.2)	0.02
Height for weight z score (HWZ)	−0.2 (−0.4 to −0.03)	−0.1 (−0.04 to −0.3)	0.02

Child cognition, language and motor scores were measured using the Bayley Scales of Infant and Toddler Development-version III. Knowledge of child rearing practices was measured with a structured questionnaire used in previous studies (20 questions, potential range of scores: 0–60). Fathers' engagement were scored from five activities (yes = 1/no = 0): reading a book, telling story, playing, singing song or rhymes and drawing or counting to child. Total home stimulation environment was assessed using Family Care Indicators (24 questions, potential range of scores: 0–24). Linear regression analyses were adjusted for child age and sex, tester/interviewer, asset index, baseline corresponding score as fixed effects and cluster as a random effect. 1 = intervention, 0 = control. ^$^, logistic regression. 1 = yes (mother experienced violence), 0 = no (mothers did not experience violence).

For the secondary outcomes, the mothers of the intervention group experienced less domestic violence [Odds ratio; 0.6 (95% CI: 0.4–1.0)] and the fathers were more engaged in child development activities at home [β; 0.3 (95% CI: 0.03–0.5) (Effect size; 0.23 SD, 95% CI: 0.39–0.06) compared to the comparison group. Home environmental stimulation [β: 2.6 (95% CI: 2.1–3.1)] (Effect size; 0.82 SD, 95% CI: 0.99–0.64)] and mothers' knowledge on child care [β: 3.8 (95% CI: 2.5–5.1) (Effect size; 0.64 SD, 95% CI: 0.81–0.47)] were increased in the intervention group. Nonetheless, the children's growth was not improved.

Post hoc mediation analyses showed that total home stimulation and the mothers' knowledge on childcare significantly mediated the effect of the intervention on children's cognitive and language development (Table S3).

We found that the intervention had no differential effect of the intervention in younger mothers (age <25 years) compared to older mothers (age ≥25 years) (Figs. S1 & S2). Cognitive and language development of children did not differ by their sex (Figs. S3 & S4). Moreover, we found that there were no significant interactions between the mothers' age and the intervention or the children's sex and the intervention. The children of more educated mothers had higher language scores (Fig S5) but were similar in their cognitive scores. There was also no interaction between the mothers' education and the intervention (Fig S5).

## Discussion

This trial showed that ECD-focused parenting intervention through home visits using a social safety net (unconditional cash transfer) programme improved children's cognitive, language and motor development in the deprived settings of urban Bangladesh. We found that the intervention reduced domestic violence against mothers, improved father engagement in child development activities, total home environmental stimulation and the mother's childcare knowledge. The current study was a first step towards a scalable and effective ECD focused parenting intervention for urban Bangladesh through home visits. The positive findings demonstrate for the first time that the Jamaican Reach Up and Learn model with appropriate cultural adaptations can be successfully delivered in deprived settings of urban Bangladesh using a social safety net programme. Moreover, the findings indicate that the model is effective when run and managed by the CHWs for relatively young and deprived mothers and their children in urban settings in LMICs.

The results of the mediation analysis suggest that a stimulatory environment at home and maternal child-rearing knowledge played a significant role in mediating the effect of the intervention on children's cognitive and language development.

The lack of a differential effect of treatment in younger mothers or by children's sex suggests that the intervention was effective for all children regardless of the children's gender or the mothers' age. However, the children of more educated mothers had higher language scores, indicating that better educated mothers influenced their children's language development.

The impact of the ECD focused parenting intervention through home visits on cognitive and language development was comparable with the previous studies conducted in Bangladesh^24^, ^25^, ^26^^,^^40^ and other LMICs.^13^ Most of the ECD interventions using the Reach Up and Learn curriculum were operated in rural settings and we did not find any ECD interventions using social safety net programmes in urban deprived settings in low- and middle-income countries that would have been comparable with our findings. One study, conducted in an urban slum iin India which included all children in one cluster, was able to produce an impact on cognitive and language development,^13^ but the effect size was smaller than that of our study. Two studies in Bangladesh using community clinics in rural settings, conducted sessions with the pairs or groups of mother-child dyads, produced higher effect sizes on cognitive and language development of children and the authors assumed that it this was due to the involvement of professional health providers in the community who were familiar with and were respected by the participants.^24^^,^^26^ However, the authors noted that the Bangladeshi primary health care system does not have adequate coverage to include all children in rural areas.

The intervention programmes in urban Brazil and rural Madagascar did not show any impact on children's cognitive and language development. In the Brazilian study, the authors stressed the importance of paying attention to staff workloads when integrating parenting programmes into other services and providing ongoing supervision for quality intervention.^29^ The Madagascar study was conducted in a geographically dispersed area with low population density and high food insecurity. Moreover, the play materials were not left at home after each session.^30^ In our study, we recruited a full-time supervisor (female) who ensured the ongoing quality of sessions of the intervention delivered by CHWs. We also deployed two male field organizers who regularly travelled on motor bikes and checked in with CHWs to ascertain required play materials were all accounted for. They also monitored the availability of play materials left in the households so the children could continue using the toys. These field organizers oversaw the production and distribution of many play materials that were produced in the project area, which further provides support for the project's potential scalability (transportation of play materials from Dhaka to the Rangpur project area was costly and time consuming).

We recruited and trained female residents in the urban area to deliver the parenting intervention to ensure the scalability of the programme. The CHWs conducted 25 fortnightly sessions in one year and no CHWs were dropped from the study. Evidence supports the idea that the use of adequately trained women living in the same communities instead of externally hired fieldworkers boosts the sustainability and acceptance of large-scale ECD/parenting programmes.^14^ This is very important finding for urban areas where primary health care systems like the Bangladeshi health system, are unable to deliver ECD interventions at an early age due to a lack of available health infrastructures.

The urban primary health care systems differ from the rural system in Bangladesh and therefore cannot be compared to the rural settings. In addition, urban poor people do not have easy access to health centres as a result of supply and demand barriers to health service utilization. Hence, ECD-focused parenting and nutrition education through home visits can play an important role in children's development. While there are many approaches to ECD intervention, home visits remain one of the most effective ways to reach low income families, and reduce the intergenerational effects of severe poverty, disadvantage, and attachment disruption on a range of health, socioemotional and other related outcomes.^41^ Some recent work in high-income countries has found home visiting to be scalable, but little data is available in LMICs.^42^ In fact, for a variety of reasons, where the CHWs' role is practical (formative) at the clinic, it becomes more supportive in the home during a home visit which helps mothers or other family members discuss important issues, potentially bringing additional benefits to the mothers or the children.^43^

Our model benefited children's development and could be a model for urban areas where the primary health care systems are unable to cover ECD interventions for the poor, which may bring additional unexplored benefits. Our intervention reduced domestic violence against women, although the means through which this was achieved is not clear to us. A patriarchal society easily understand that intimate partner violence and victimization may severely compromise women's ability to parenting and provide for their children's needs.^44^^,^^45^ Intervention focused on children may change the family dynamics positively and help the mothers act properly with the support of male family members for the well-being of the children.^46^^,^^47^ Thus, the intervention ultimately benefited the welfare of the mothers.^46^^,^^48^ During the delivery of the home visiting programme, mothers or fathers had the opportunity to discuss many related social issues, and the providers might have given them social support. Therefore, it is possible that social support provided to the mothers during home visits, which we were not specifically targeting, might be indirectly responsible for this reduction. Further, the home-visit approach involves a range of family members, including the fathers, who, along with other perpetrators in the family, might have been promoted to commit less domestic violence. Home visits also decrease barriers related to transportation and distance that can hinder a centre based ECD delivery approach. Complementing centre-based delivery approaches with ECD home-visits and group work interventions can augment the range of services provided, further enhancing the impact of ECD investment through reaching the underprivileged and by addressing multifaceted family issues. Active coaching and strength-based models also help families draw on their own resources to better navigate formal and informal support structures.^49^

We found that the intervention had no differential impact on children's development based on the mothers' age or the children's gender, however, children of better educated mothers had better language development. Our findings suggest that if young mothers are well coached, they can better care for their children. This finding may encourage policymakers to scale-up the intervention in Bangladesh and other low-resource settings where most mothers are very young. On the other hand, during the scale-up of this intervention package, special attention must be paid to language development training of CHWs, so that all mothers have the potential to achieve the expected milestones of language development for their children. The literature supports the idea that girl children in urban slums are at greater risk for poverty-related problems like illiteracy, malnutrition, the adoption of unscientific health practices, early marriage, and early pregnancy.^50^^,^^51^ Hence, issues facing the urban poor girl child as a prospective mother should be focused on in the national policy as well as in the action plan for children's development. Our present intervention improved poor girls' and boys' cognitive and language development equally, therefore, this package could be one of the early life interventions that may reduce inequality even in later life. However, the follow-up of these children in the future is crucial.

Our study has several strengths, including the use of a well-balanced cluster-randomized study design, representative sample size, direct assessments (use of Bayley III) of child development, masked assessors and intention-to-treat analyses. The outcome measures had good psychometric properties. Although the Bayley Scales are not standardized for Bangladeshi children, they have good concurrent validity.^24^ The GoB is strongly committed to promoting early childhood development, but there are no evidence-based ECD intervention models that can be delivered through a national social safety net platform in urban, deprived areas. The GOB can introduce this model integrated into the upcoming Maternal and Child Benefit Programme (MCBP) to improve parenting practices and children's development in the country, especially in urban areas where the primary health care system is fragmented and patchy.

The most important study similar in nature that was conducted in Jamaica using weekly sessions for two years and had an effect size of 0.76 SD on cognitive development.^10^ The limitations in our study were the less frequent sessions and shorter durations which might not have been sufficient to demonstrate similar benefits for these children. The interventions did not improve children's growth, perhaps because the CHWs focused more on child developmental activities than nutrition education. Besides, the poverty-stricken children may require earlier food supplementation so as they do not become malnourished. Another risk of bias could be selection bias but, unfortunately, we had no control over the selection of participants. As cash transfer was used as part of the intervention, it is possible that some otherwise ineligible mothers from better-off populations might have wanted to be involved for financial gain. However, this did not affect the difference of the probability of receiving cash between the arms. Another bias could be the use of Bayley tools which are not standardized for Bangladeshi children. We had, however, previously validated them to Bangladeshi culture and changed some of the unfamiliar pictures. We have used the validated tools in a number of studies in Bangladeshi settings.^25^^,^^27^

Additional research focusing on ECD intervention at an earlier age along with the continuation of other services, such as pre-primary education and primary education, to school age and beyond, may be helpful in understanding which aspect of the intervention is bringing the maximum benefit. Moreover, follow-up of these children would be important to be able to explore long-term benefits of the intervention.

The ECD-focused parenting and nutrition education programme through home visits using social safety net programme proves to be feasible and effective for children's development in urban contexts. This evidence may aid in the scaling up of the intervention and furthering the coverage of ECD programmes in other deprived urban settings in LMICs.

## Contributors

Sheikh Jamal Hossain conceived the study. Sheikh Jamal Hossain, Jena D Hamadani designed the study. Jena D Hamadani, Fahmida Tofail and Sheikh Jamal Hossain led data collection. Sheikh Jamal Hossain and Jena D Hamadani did the statistical analyses. Sheikh Jamal Hossain wrote the first draft of the manuscript. All authors critically reviewed the manuscript. All authors approved the final draft.

## Data sharing statement

Data belongs to International Centre for Diarrhoeal Disease Research Bangladesh (icddr,b) and sharing with a third party is not allowed. All requests to obtain the original data must therefore be addressed to this authority. icddr,b's Department of Research Administration maintains a data repository and a copy of the complete dataset of this study will remain in the repository. Interested researchers may contact with corresponding author to access the data.

## Declaration of interests

The authors declare no conflict of interest.

## Appendix A Supplementary data

Supplementary Figures and Tables

Protocol_ERC
