# Supplementary material for: Effect of a parenting and nutrition education programme on development and growth of children using a social safety-net platform in urban Bangladesh: a cluster randomized controlled trial
Source: Lancet Reg Health Southeast Asia. 2024 Mar 19;25:100388. doi: 10.1016/j.lansea.2024.100388 (PMC10965454; doi:10.1016/j.lansea.2024.100388)
Supplement: Supplementary Figures and Tables [file mmc1.docx]

Contents

[Table S1. Child, family and maternal characteristics by lost at post-intervention 2](#_Toc154510085)

[Table S2: Internal reliabilities of questionnaires at baseline and post-postintervention 3](#_Toc154510086)

[Table S3: Mediation analyses with stimulation in the home (Family Care Indicators-FCI) and mothers’ knowledge on child care for children’s cognitive development. 3](#_Toc154510087)

[Table S4: Mediation analyses with stimulation in the home (Family Care Indicators-FCI) and mothers’ knowledge on child care for children’s language development. 3](#_Toc154510088)

[Figure S1: Children’s cognitive development by mother’s age at enrollment and post-intervention 4](#_Toc154510089)

[Figure S2: Children’s language development by mother’s age at enrollment and post-intervention 5](#_Toc154510090)

[Figure S3: Children’s cognitive development by their sex at enrollment and post-intervention 6](#_Toc154510091)

[Figure S4: Children’s language development by their sex at enrollment and post-intervention 7](#_Toc154510092)

[Figure S5: Children’s language development by their mothers’ education at enrollment and post-Intervention 8](#_Toc154510093)

# **Table S1. Child, family and maternal characteristics by lost at post-intervention**

| **Characteristics** | **Tested**  (N=562)  Mean (SD)/ | **Lost**  (N=37)  Mean (SD)/ | **P value** |
| --- | --- | --- | --- |
| Mothers’ Age | 25.7 (5.1) | 24.8 (4.7) | 0.28 |
| Mothers’ BMI | 22.9 (4.2) | 23.3 (4.6) | 0.56 |
| Mothers’ Education | 7.0 (3.5) | 7.9 (4.0) | 0.12 |
| Children’s Age | 11.3 (3.2) | 11.7 (3.2) | 0.50 |
| Outcomes | | | |
| Children’s cognitive development | 94.1 (10.9) | 92.8 (12.0) | 0.50 |
| Children’s language development | 91.6 (12.8) | 88.7 (13.1) | 0.18 |
| Children’s motor development | 93.0 (14.9) | 91.1 (17.6) | 0.27 |
| Home environment | 5.6 (3.2) | 5.8 (2.7) | 0.82 |
| Child caring knowledge | 25.4 (6.5) | 24.2 (7.3) | 0.27 |
| Child cognition, language and motor scores were measured using the Bayley Scales of Infant and Toddler Scale-version III. Knowledge of child rearing practices was measured with a structured questionnaire used in previous studies (20 questions, potential range of scores: 0–60). Home stimulation was assessed using Family Care Indicators (24 questions, potential range of scores: 0–24). | | | |

# **Table S2: Internal reliabilities of questionnaires at baseline and post-postintervention**

| Measurement | Internal Reliability (Cronbach’s alpha) | |
| --- | --- | --- |
|  | Base line | End line |
| Mothers’ knowledge on child care | 0.8 | 0.8 |
| Home stimulation environment (Family Care Indicators) | 0.7 | 0.8 |
| Household food security status | 0.9 | 0.9 |

# **Table S3: Mediation analyses with stimulation in the home (Family Care Indicators-FCI) and mothers’ knowledge on child care for children’s cognitive development.**

|  | Total Effect | | | Indirect Effect | | | Direct Effect | | |
| --- | --- | --- | --- | --- | --- | --- | --- | --- | --- |
|  | Coefficient | T value | P-value | Coefficient | T value | P-value | Coefficient | T value | P-value |
| Family Care Indicator | 0.6 | 3.4 | 0.001 | 0.2 | 4.7 | <0.001 | 0.4 | 2.2 | 0.03 |
| Mothers’ knowledge on child care | 0.6 | 3.7 | <0.001 | 0.2 | 4.1 | <0.001 | 0.4 | 2.4 | 0.02 |
| Structural equation model was followed to see if the family care indicator or mothers’ knowledge on child care is mediator.1=intervention, 0=control. For family care indicator, independent variables were child age and sex, tester, baseline corresponding cognitive score and baseline score for family care indicator. For mothers’ knowledge on child care, independent variables were child age and sex, tester, baseline corresponding cognitive score and baseline score of mothers’ child care knowledge. | | | | | | | | | |

# **Table S4: Mediation analyses with stimulation in the home (Family Care Indicators-FCI) and mothers’ knowledge on child care for children’s language development.**

|  | Total Effect | | | Indirect Effect | | | Direct Effect | | |
| --- | --- | --- | --- | --- | --- | --- | --- | --- | --- |
|  | Coefficient | T value | P-value | Coefficient | T value | P-value | Coefficient | T value | P-value |
| Family Care Indicator | 3.6 | 3.0 | 0.003 | 2.3 | 4.4 | <0.001 | 1.3 | 1.0 | 0.34 |
| Mothers’ knowledge on child care | 3.0 | 3.1 | 0.002 | 1.9 | 4.7 | <0.001 | 1.1 | 1.2 | 0.24 |
| Structural equation model was followed to see if the family care indicator or mothers’ knowledge on child care is mediator.1=intervention, 0=control. For family care indicator, independent variables were child age and sex, tester, baseline corresponding cognitive score and baseline score for family care indicator. For mothers’ knowledge on child care, independent variables were child age and sex, tester, baseline corresponding cognitive score and baseline score of mothers’ child care knowledge. | | | | | | | | | |


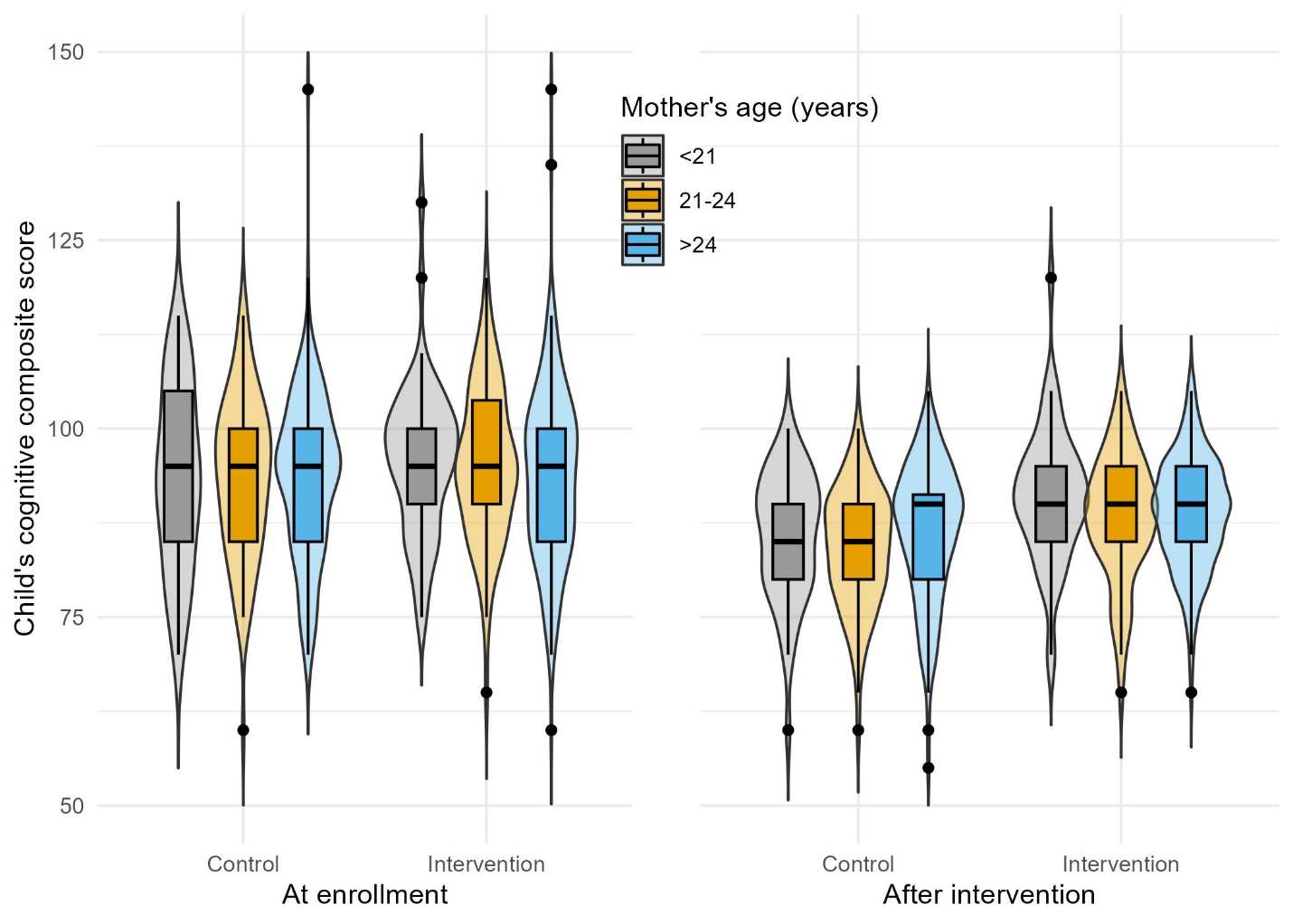


# **Figure S1: Children’s cognitive development by mother’s age at enrollment and post-intervention**

(Footnote:The figure depicts the distribution of the children’s cognitive composite scores at enrollment and after intervention by their groups- control and intervention with consideration of maternal age groups- <21 years, 21-24 years and >24 years. The distribution of the scores is presented in violin plots and box-scatter plots that show the minimum, quartile-1, median, quartile-3, and maximum scores in Y-axis. The width of the violins indicates the spread of the values and the few numbers of dots tell us about the extreme values. The colors of the violins and boxes are illustrated in the legends.)


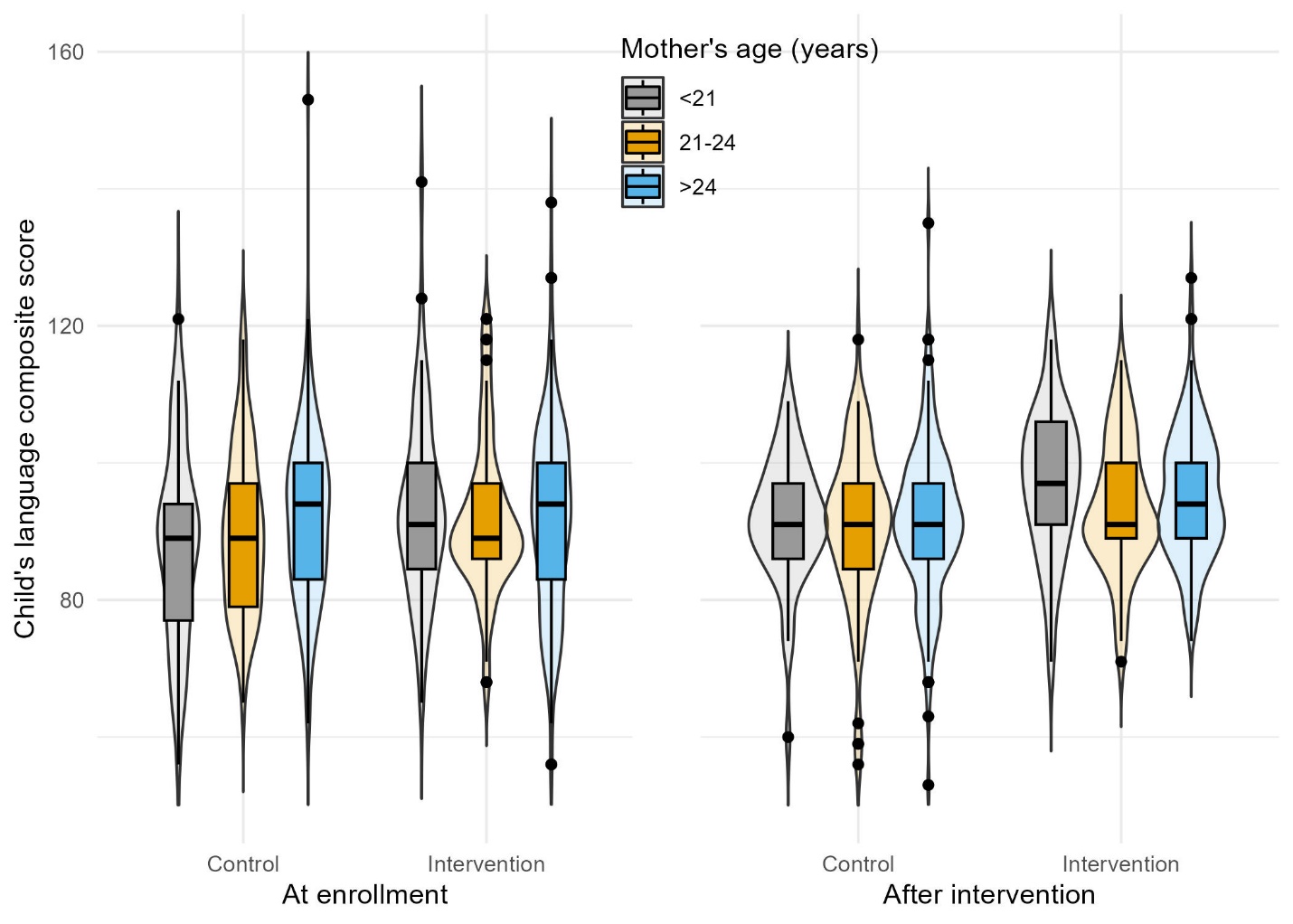


# **Figure S2: Children’s language development by mother’s age at enrollment and post-intervention**

(Footnote: The figure depicts the distribution of the children’s language composite scores at enrollment and after intervention by their groups- control and intervention with consideration of maternal age groups- <21 years, 21-24 years and >24 years. The distribution of the scores is presented in violin plots and box-scatter plots that show the minimum, quartile-1, median, quartile-3, and maximum scores in Y-axis. The width of the violins indicates the spread of the values and the few numbers of dots tell us about the extreme values. The colors of the violins and boxes are illustrated in the legends.)


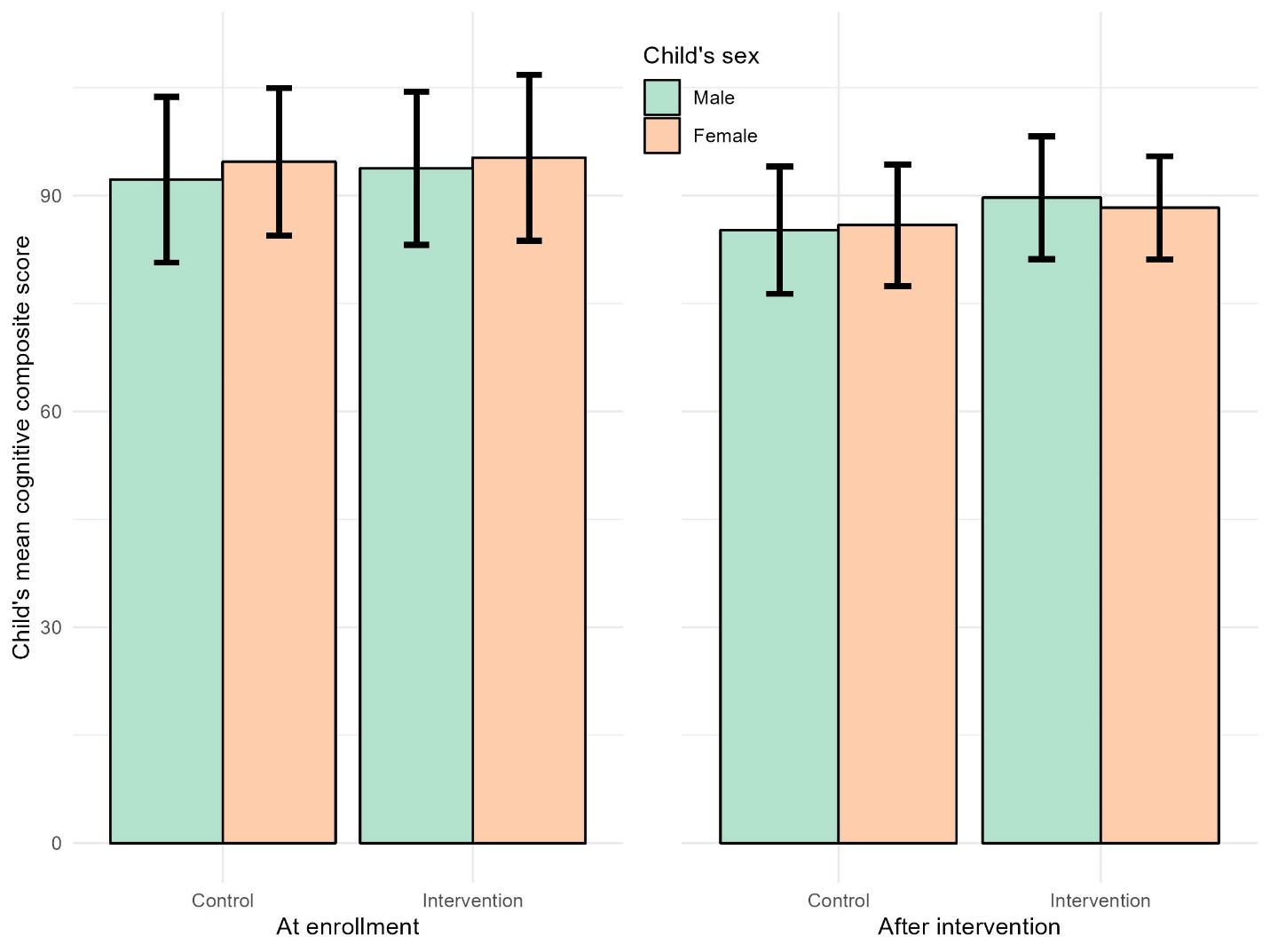


# **Figure S3: Children’s cognitive development by their sex at enrollment and post-intervention**

(Footnote: The figure depicts the mean and standard deviations (SDs) of the children’s cognitive composite scores at enrollment and after intervention by their groups- control and intervention along with consideration of their sex. The height of the bars indicates the means and the spikes indicate the SDs from the means, in Y-axis. The colors of the bars are illustrated in the legends.)


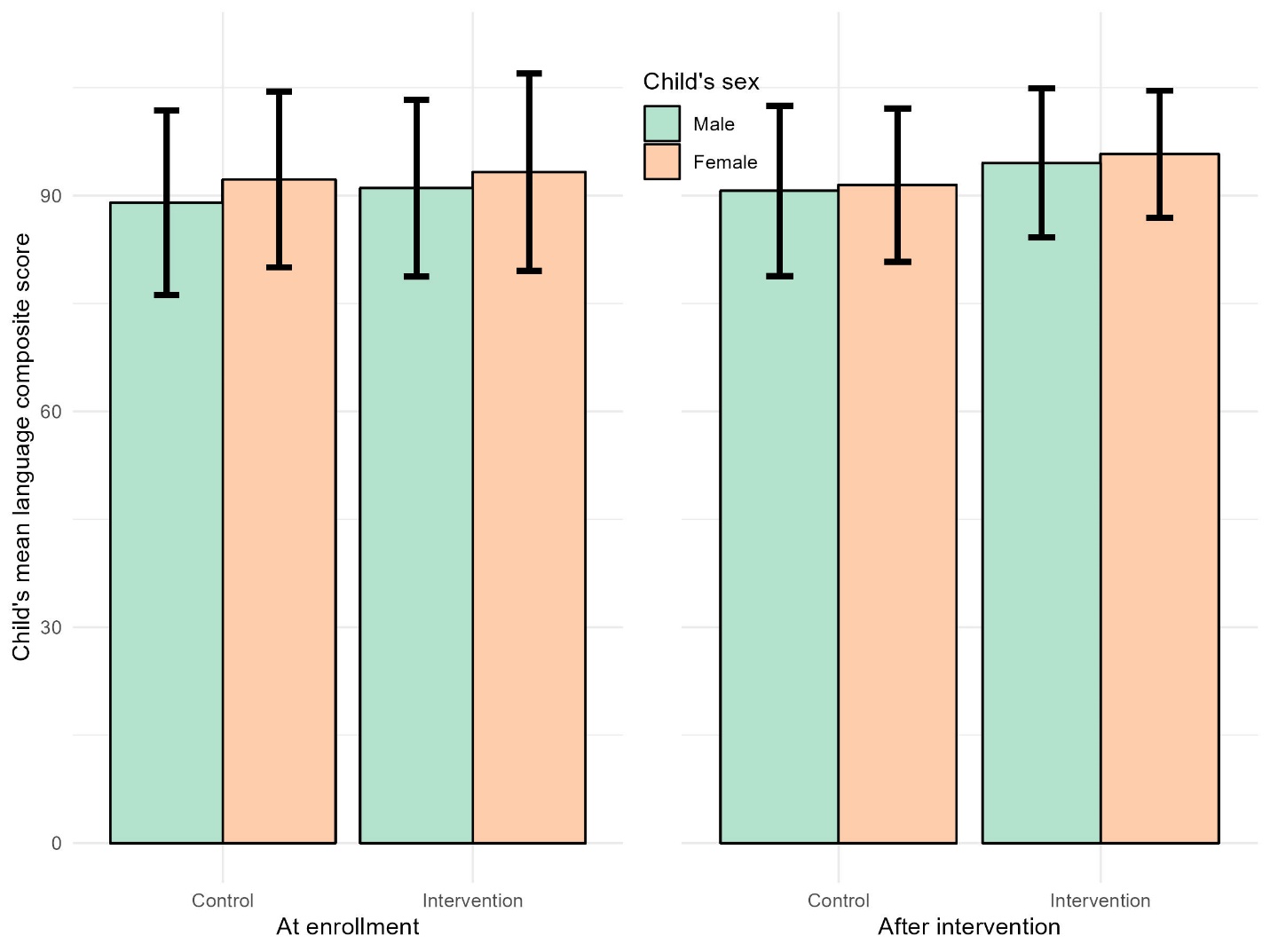


# **Figure S4: Children’s language development by their sex at enrollment and post-intervention**

(Footnote: The figure depicts the mean and standard deviations (SDs) of the children’s language composite scores at enrollment and after intervention by their groups- control and intervention along with consideration of their sex. The height of the bars indicates the means and the spikes indicate the SDs from the means, in Y-axis. The colors of the bars are illustrated in the legends.


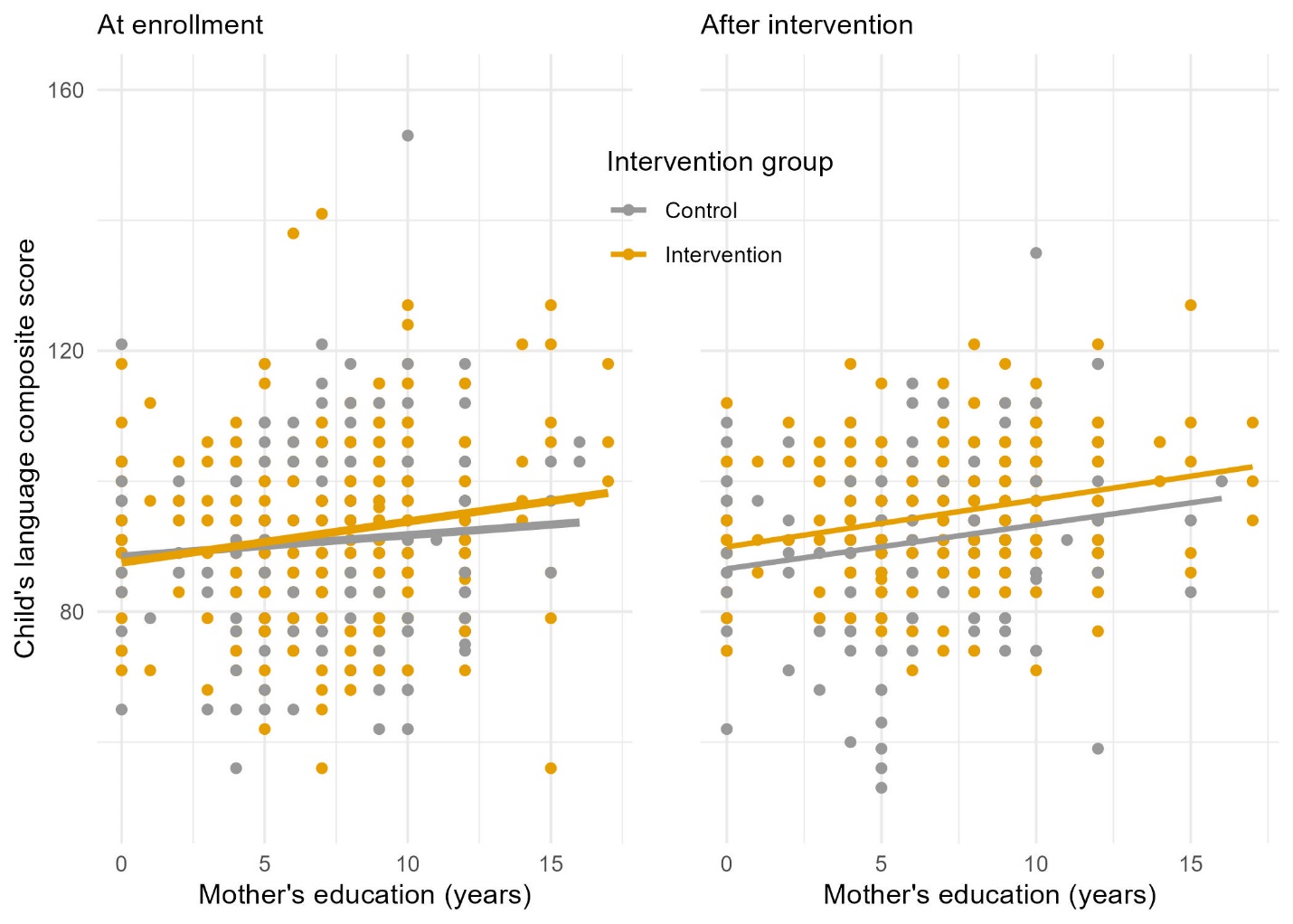


# **Figure S5: Children’s language development by their mothers’ education at enrollment and post-Intervention**

(Footnote: The figure depicts the distribution of the children’s language composite scores by their maternal education in completed years at enrollment and after intervention phases. The lines through the scatter plot are fitted for regression model considering the scores as response and maternal age and intervention group as predictors. The colors of the dots and the lines are illustrated for the intervention groups in the legends)
