# Supplementary material for: Effect of a parenting and nutrition education programme on development and growth of children using a social safety-net platform in urban Bangladesh: a cluster randomized controlled trial
Source: Lancet Reg Health Southeast Asia. 2024 Mar 19;25:100388. doi: 10.1016/j.lansea.2024.100388 (PMC10965454; doi:10.1016/j.lansea.2024.100388)
Supplement: Protocol_ERC [file mmc2.pdf]

Memorandum

15 July, 2019

To:

Chairperson

Research Review Committee (RRC)

From : Sheikh Jamal Hossain

Principal Investigator of research protocol # PR-18035

Maternal and Child Health Division

Sub: Approval of additional two external coinvestigators in the Research Protocol # PR-18035

Dear Sir,

With due respect, here we are sending our research protocol # PR-18035 on "Evaluating effect of integrating ECD activities into Bangladesh government's urban lactating mothers allowance program for the poor on children's cognition and behaviour" for your kind approval. Please note that we have added two external coinvestigators in this study so that we can exchange our thought on this topic.

icddr,b

ADDENDUM REQUEST FORM

PART-I: Protocol Information

Protocol Number: PR-18035

Protocol Title: **Evaluating effects of integrating ECD activities into Bangladesh Government's urban lactating mothers allowance program for the poor on children's cognition and behaviour in Rangpur city corporation, Bangladesh**

Principal Investigator: Sheikh Jamal Hossain Division: MCHD

Date of approval: RRC Date: 20 May, 2018 ERC Date: 1 July, 2018 AEEC

Have the protocol activities been started? ☐ No ☒ Yes Date of starting: 1st July. 2018

If yes, current status of implementation of the research protocol
(Check all boxes that are applicable):

☐ Continuing enrolment of the study participants
☐ Enrollment closed but follow-up or data analysis are ongoing
☐ Ongoing laboratory testing
☐ Study activities only involve data analysis or manuscript writing
☒ Others, please specify: We have just selected the intervention (study) area.

Indicate if this is the first addendum proposal to the research protocol? ☒Yes ☐ No

If No, Part II of the form to be completed

PART-II: Particulars of previously approved addendum of the research Protocol

Number	Description of approved addendum of the research protocol	Approval dates		
		RRC	ERC	AEEC

PART-III: Particulars of proposed addendum

Proposed changes affects: (check all boxes that are applicable)

☐ Investigator(s)
☐ Study objective(s)
☐ Research procedure(s)
☐ Number of participants to be enrolled
☐ Age and/or sex group of the study participants or addition of special group(s) e.g. pregnant women, malnourished children
☐ Eligibility (inclusion and/or exclusion) criteria
☐ Intervention (drug/vaccine formulation or dosing) or devise
☐ Collection of biological samples (type, number, amount, etc)
☐ Consent process
☐ Consent forms
☒ Study instrument (Health Economic (parental time) questionnaire has been added)
☒ Study sites
☐ Compensation for participation in research (e.g. increasing/decreasing the amount)
☐ Data Collection/analysis
☐ Budget
☐ Others, specify:

A. Provide itemised description of the proposed changes with **justifications**.

1. Study sites: Now, we have selected Rangpur City Corporation in lieu of Savar Pouroshova. There are a number of mothers who do not stay at study area (urban savar) but receive lactating allowance. So we will be unable to conduct our intervention outside the Pouroshova of Savar for one year.
2. Study instrument: Adding Indirect Cost Measurement Questionnaire (Parental Time)
3.
4.

B. Please respond to the following:

a) Is the request based on any new finding(s)?

☐ Yes ☒ No

If yes, describe the significance of the finding(s) (e.g. new adverse event) available during the course of research, or information concerning requested change(s) that may influence study participants' willingness to continue participation. In such events, the PI shall modify the consent form(s) and apply that for re-consenting of participants already enrolled in the study.

b) Will the requested change(s) alter/likely to alter the scientific validity of the study?

☐ Yes ☒ No

If yes, explain in detail

c) Do any of the proposed change(s) alter the risk (physical, psychological and sociological) /or benefit to the study participants?

☐ Yes ☒ No

If yes, explain in detail:

d) If answer to the question # (c) is 'YES', will the enrolled participants be willing to remain in the study;

☐ Yes ☐ No

If yes, describe how this will be done and mention if the study participants need to be informed or re-consented.

e) Do the proposed change(s) affect any other service benefits?

☐ Yes ☒ No

If yes, explain in detail:

f) Any other relevant information, which might not have been covered above:

I understand that I cannot initiate any change in the approved research protocol until my requested change(s) is/are approved by all relevant Committees.

Signature of the Principal Investigator	Signature of Division Director
Date:	Date:

Attachment 1 **(FACE SHEET)** Date: 14-07-2019

ETHICAL REVIEW COMMITTEE, ICDDR,B

Principal Investigator: Sheikh Jamal Hossain
Protocol Number: | P | R | 1 | 8 | 0 | 3 | 5 |

Protocol Title: Evaluating effects of integrating ECD activities into Bangladesh Government's urban lactating mothers allowance program for the poor on children's cognition and behaviour in Savar

Trainee Investigator (if any): Yes ☐ No ☒
Student Investigator (if any): Yes ☐ No ☒

Project Status: RRC approved

☒ New Study
☐ Secondary data analysis (Skip 2, 4 & 5)

Check the appropriate box to answer to each of the following (If Not Applicable write NA)

		Yes	No
1.	Source of population:		
(a)	Ill participants	☐	☒
(b)	Non-ill participants	☒	☐
(c)	Minor or persons under guardianship	☒	☐
(d)	Others :	☐	☒
2.	Does the study involve:		
(a)	Physical risk to the participants	☐	☒
(b)	Social risk to the participants	☐	☒
(c)	Psychological risks to participants	☐	☒
(d)	Discomfort to participants	☒	☐
(e)	Invasion of participants' privacy	☐	☒
(f)	Disclosure of information damaging to participants or others	☐	☒
3.	Does the study involve:		
(a)	Use of records (hospital, medical, death or other)	☒	☐
(b)	Use of fetal tissue or abortus	☐	☒
(c)	Use of organs or body fluids	☐	☒
(d)	Use of stored biological specimens	☐	☒
(e)	Use of already collected data	☐	☒
4.	Are participants clearly informed about:		
(a)	Nature and purposes of the study	☒	☐
(b)	Procedures to be followed including alternatives used	☒	☐
(c)	Physical risk	☒	☐
(d)	Sensitive questions	☒	☐
(e)	Benefits to be derived	☒	☐
(f)	Right to refuse to participate or to withdraw from the study	☒	☐
(g)	Confidential handling of data	☒	☐
(h)	Compensation and/or treatment where there are risks or privacy is involved in any particular procedure	☒	☐

		Yes	No
5.	Will informed consent be obtained from		
	(a) Study participants	☐	☒
	(b) Parent or guardian or next to kin (if study participants are minor and/or under guardianship)	☒	☐
	(c) Participant aged 11 – 17 years (Assent)	☐	☐
6.	Will precautions be taken to protect anonymity of study participants	☒	☐

7. Check documents being submitted herewith to Committee:

☐ Umbrella proposal - Initially submit an overview (all other requirements will be submitted with individual research protocol)

Research protocol should include:

☒ Abstract Summary
☐ Consent form for study participants
☒ Consent form for parent or guardian or next to kin
☐ Assent form for participant under Guardianship
☒ Questionnaire*

* If the final instrument is not ready at the time of submission of the protocol for review by the ERC, the following information should be included in the abstract summary.

1 Issues to be covered in the questionnaire or interview which could be considered either sensitive or which would constitute an invasion of privacy.

2 The final questionnaire must be approved by the committee before its use.

We agree to obtain approval of the Ethical Review Committee for any changes involving the rights and welfare of study participants before making such changes.

Principal Investigator	Trainee investigator	Student investigaton

icddr,b
KNOWLEDGE FOR GLOBAL LIFESAVING SOLUTIONS

Attachment 1a

INFORMATION TO INCLUDE IN ABSTRACT SUMMARY

The Committee will not consider any application, which does not include an abstract summary. The abstract should summarize the purpose of the study, the methods and procedures to be used, by addressing each of the following items. If an item is not applicable, please note accordingly:

1. Describe the requirements for a subject population and explain the rationale for using in this population special groups such as children, or groups whose ability to give voluntary informed consents may be in question.

   RESPONSE: This is a cluster randomized controlled trial with 2 arms: i) Psychosocial stimulation+ Lactational Allowance (LA) and ii) LA only.

   We will recruit 300 children in each arm. We have set inclusion and exclusion criteria for recruitment of the children.

   Inclusion criteria:
   Mothers with a child aged 6-16 months.
   Eligible to receive LA.
   Not expected to leave the study site for more than 2 months.
   Has a legally acceptable representative capable of understanding the informed consent document and providing consent on the participant's behalf.

   Exclusion criteria:
   Legal guardian unwilling or unable to provide written informed consent.
   Known congenital anomaly, developmental disorder or severe developmental delay.
   If not possible to test the child due to physical or behavioural problems
   Children of multiple birth e.g. twin, triplets

   We will seek consent from the children's parent or their guardians.

2. Describe and assess any potential risks – physical, psychological, social, legal or other – and assess the likelihood and seriousness of such risks. If methods of research create potential risks, describe other methods, if any, that were considered and why they will not be used.

   RESPONSE: There are no major risks involved in participating in this study.

3. Describe procedures for protecting against or minimizing potential risks and an assessment of their likely effectiveness.

   RESPONSE: There are no minimum risks of this research for the project. If the child becomes sick during the study we will refer him/her to hospital under our guidance.

4. Include a description of the methods for safeguarding confidentiality or protecting anonymity.

   RESPONSE: Consent forms and all data will be linked by identifiers and will be treated with strict confidence throughout and beyond the duration of the trial.

5. When there are potential risks to the subject, or the privacy of the individual may be involved, the investigator is required to obtain a signed informed consent statement from the subject. For minors, informed consent must be obtained from the authorized legal guardian or parents of the subject. Describe consent procedures to be followed including how and where informed consent will be obtained.

   a) If signed consent will not be obtained, explain why this requirement should be waived and provide an alternative procedure.

      RESPONSE: NA

b) If information is to be withheld from a subject, justify this course of action.

RESPONSE: NA

c) If there is a potential risk to the subject or privacy of the individual is involved in any particular procedure include a statement in the consent form stating whether or not compensation and/or treatment will be available.

RESPONSE: It is included in the consent form

6. If study involves an interview, describe where and in what context the interview will take place. State approximate length of time required for the interview.

RESPONSE:

If the participants agree to participate in the study, we will visit her and her child and collect some information about your quality of life, food availability in your family, your income and expenditure, how you seek health during any illness, any violence experienced by you in your family, type of toys and stimulation activities your child receives at home, etc. This visit will take about 40-50 minutes of her time. We will also bring her and her child at the beginning of the study and after a year to a nearby local office and see how your child plays with some toys to assess his/her behaviour and brain development. At that session, we will measure your child's weight, length, arm and head circumference, your weight, height and arm circumference, your depressive symptoms and collect information on your socio-economic condition. This session takes about one hour. In addition, we will select some of the participants, based on lottery to be visited every fortnight by an intervention worker who will discuss with you how to play and stimulate your child. The fortnightly visits will take about one hour.

7. Assess the potential benefits to be gained by the individual subject as well as the benefits which may accrue to society in general as a result of the planned work. Indicate how the benefits outweigh the risks.

RESPONSE: The parents will be able to know the child's development and anthropometry status and if there is any problem, we will refer the child for appropriate treatment. By participating in this study, the participants will be contributing to the development of science and the results can be shared with policy makers for adoption of new methods.

8. State if the activity requires the use of records (hospital, medical, birth, death or other), organs, tissues, body fluids, the fetus or the abortus.

The statement to the subject should include information specified in item 2,3,4,5(c) and 7 as well as indicating the approximate time required for participation in the activity.

RESPONSE: We will collect a list of LA recipients from the government authorities.

Memorandum

28 June 2018

To: Professor Saria Tasnim

Chairperson
Ethical Review Committee (ERC)

From: Mr Sheikh Jamal Hossain
Principal Investigator of research protocol # PR-18035
Maternal and Child Health Division (MCHD)

Sub: Ethical approval of Research protocol # PR-18035

Thank you for your feedback on research protocol # PR-18035 titled "Evaluating effect of integrating ECD activities into Bangladesh government's urban lactating mothers allowance program for the poor on children's cognition and behaviour in Savar, Bangladesh". Please find our response under each comment for your review and kind approval.

a) Face sheet 1(a) should be marked as No.

Response: Thank you. We have corrected in the face sheet.

b) Face sheet 2(d) should be marked as Yes. There are many questions to be asked.

Response: Thank you. We have corrected in the face sheet. The participants may feel little discomfort.

c) Abstract summary: 6th question – answer is not given, rather it is written it is included in consent form. Abstract summary is self-explanatory, better to give proper response.

Response: We have added the text in the abstract

d) Main protocol page 15: Design and Method- In method it is written that investigator will understand and analyze bottleneck of ECD services and document all these ECD activities. Whereas in the title, it is written integrated ECD activities, which means you would have additional component in ECD. Methodology stage-1 is not reflected in your title. Moreover, in Methodology stage-2, you would have two intervention arms, there is no control arm. How would you interpret your result if there is no control arm?

Response: We will conduct a survey in the study area to understand nature of ECD and its bottlenecks through a Tanahsi framework. We will also seek information from this survey who, when, where and how the services is delivered. With these information we will share with stakeholders and relevant expertise to select a platform to deliver of our stimulation package. If we try to incorporate our this objective in the title the title will be misleading. So we avoid it.

You are right we do not have pure comparison group. Our study aims to compare if psychosocial stimulation works when added to lactating allowance in urban area. So the participants who are receiving only lactating allowance group will be the comparison group for our study.

e) Questionnaire- page 40, 48, 52, 55, 57, 59, 60, 64: Project title is given as LAPS- how relevant it is? Only study title can be written. LAPS was not introduced in this protocol.

Response: we have deleted those words

f) Consent form:

i) In Bangla Consent it is written 600 participants will be recruited

ii) In English Consent form it is written 660 participants will be recruited and there will be 4 arms, each arm with 165. In the entire protocol it was never seen with 4 arms.

iii) Major revision is needed in consent forms

Response: Thank you for your comments. We have corrected as per our protocol and revised consent forms.

Thank you.

Memorandum

21 June 2018

To: Mr Sheikh Jamal Hossain
Principal Investigator of research protocol # PR-18035
Maternal and Child Health Division (MCHD)

From: Professor Saria Tasnim
Chairperson
Ethical Review Committee (ERC)

Sub: Research protocol # PR-18035

Thank you very much for submitting your revised version of research protocol # PR-18035, titled "Evaluating effects of integrating ECD activities into Bangladesh Government's urban lactating mothers allowance program for the poor on children's cognition and behaviour in Savar, Bangladesh" for consideration in its May ERC meeting held on 24 May 2018. The protocol cannot be considered in the above meeting as since the primary reviewer could not review the protocol due to his official pre-occupation. However, the reviewer was advised to review the protocol through expedited review process and she made following observations on it:

a) Face sheet 1(a) should be marked as No.

b) Face sheet 2(d) should be marked as Yes. There are many questions to be asked.

c) Abstract summary: 6th question – answer is not given, rather it is written it is included in consent form. Abstract summary is self-explanatory, better to give proper response.

d) Main protocol page 15: Design and Method- In method it is written that investigator will understand and analyze bottleneck of ECD services and document all these ECD activities. Whereas in the title, it is written integrated ECD activities, which means you would have additional component in ECD. Methodology stage-1 is not reflected in your title. Moreover, in Methodology stage-2, you would have two intervention arms, there is no control arm. How would you interpret your result if there is no control arm?

e) Questionnaire- page 40, 48, 52, 55, 57, 59, 60, 64: Project title is given as LAPS- how relevant it is? Only study title can be written. LAPS was not introduced in this protocol.

f) Consent form:

  iv) In Bangla Consent it is written 600 participants will be recruited

  v) In English Consent form it is written 660 participants will be recruited and there will be 4 arms, each arm with 165. In the entire protocol it was never seen with 4 arms.

  vi) Major revision is needed in consent forms

Please modify the protocol addressing the above observations and submit a revised version of the protocol for consideration of the Chair.
Thank you.

Cc: Senior Director, MCH

Memorandum

20 May, 2018

To:

Chairperson

Research Review Committee (RRC)

From : Sheikh Jamal Hossain

Principal Investigator of research protocol # PR-18035

Maternal and Child Health Division

Sub: Approval of Research protocol # PR-18035

Dear Sir,

With due respect, here we are sending our justification and answer of your feedback of the research protocol # PR-18035 on "Evaluating effect of integrating ECD activities into Bangladesh government's urban lactating mothers allowance program for the poor on children's cognition and behaviour" for your review and kind approval. Please note that we have changed our study design based on feedback and discussion with RRC members, and now it is two arms cluster randomized controlled trial (page-16).

Feedback and answer:

a) Title: The study title is not clear. Needs to be simplified. Savar and Manikgonj Pourashova cannot be generalized for whole Bangladesh. So, the title should be revisited following area specific.

Answer: Thank you for the comment. We have now mentioned in the title: "**Evaluating effects of integrating ECD activities into Bangladesh Government's urban lactating mothers allowance program for the poor on children's cognition and behaviour in Savar, Bangladesh**"

b) Too many abbreviations were used without spelling out in its first use. A list of abbreviations should be prepared and attached with the protocol.

Answer: Thank you for the comments. We have added a list of abbreviation in the pages 21 & 22.

c) What is urban lactating allowance – needs elaboration. Is ECD services are in operation in Health Systems?

Answer: Lactating allowance activities have been elaborated in page 17 for make it more clearly. ECD services in urban area for age group under three years are very limited.

d) The "Background" could have been a little elaborate with citation of related literature in this area of research to grasp the objectives of the present research.

Answer: Thank you for your concern. We have added some new citations in the background in page 14.

e) Too many objectives are there. Can these objectives be examined by the research team once more to reduce the number without hampering the major objective?

Answer: Thank you for your suggestion. We have reduced our objectives.

f) Sample size determination could have been made more explicit.

Answer: We have elaborated our sample size calculation in page 19 & 20.

g) Data analysis plan could also be elaborated in the light of meeting study objectives.

Answer: Thank you for the comments. We have elaborated our data analysis plan in page 20.

h) ECD activities could have been described briefly to make it more meaningful to the readers.

Answer: ECD activities have been elaborated in page 17 for make it clear.

i) Page 15, under household survey: the sentence "first, respondents .... then after every five household will be selected to fill 20 respondents in each cluster" should be revisited.

Answer: Sorry for the typo. We have corrected it.

j) Data collection tools (18) – investigators mentioned about cognition and behaviour; but not mentioned to measure Food Security, Health Seeking behaviour. How Food Security behaviour is relevant to the investigators? What tools will be used for Food Security.

Answer: Thank you sir. We have added the tools for food security in the proposal.

k) Informed Consent Form: Mobile number of the PI or his representative should be given in the informed consent form so that potential study participants can contact when needed. Direct phone number (9827084) IRB Coordinator should be given instead of PABX number. The mentioned PABX number is also not right.

Answer: We have added the mobile number of PI so that the participants can contact directly PI for any query.

Thank you.

Memorandum

10 May 2018

To: Mr Sheikh Jamal Hossain
Principal Investigator of research protocol # PR-18035
Maternal and Child Health Division (MCHD)

From: Shafiqul Alam Sarker, MD, PhD
Chairperson
Research Review Committee (RRC)

Sub: <u>Research protocol # PR-18035</u>

Thank you for submitting your research protocol # PR-18035 titled "Evaluating effects of integrating ECD activities into Bangladesh Government's urban lactating mothers allowance program for the poor on children's cognition and behavior" for consideration of the RRC and present it before the Committee in its May meeting held on 07 May 2018. This is to inform you that after review and discussion, the committee made the following observations on the protocol:

l) Title: The study title is not clear. Needs to be simplified. Savar and Manikgonj Pourashova cannot be generalized for whole Bangladesh. So, the title should be revisited following area specific.

m) Too many abbreviations were used without spelling out in its first use. A list of abbreviations should be prepared and attached with the protocol.

n) What is urban lactating allowance – needs elaboration. Is ECD services are in operation in Health Systems?

o) The "Background" could have been a little elaborate with citation of related literature in this area of research to grasp the objectives of the present research.

p) Too many objectives are there. Can these objectives be examined by the research team once more to reduce the number without hampering the major objective?

q) Sample size determination could have been made more explicit.

r) Data analysis plan could also be elaborated in the light of meeting study objectives.

s) ECD activities could have been described briefly to make it more meaningful to the readers.

t) Page 15, under household survey: the sentence "first, respondents .... then after every five household will be selected to fill 20 respondents in each cluster" should be revisited.

u) Data collection tools (18) – investigators mentioned about cognition and behaviour; but not mentioned to measure Food Security, Health Seeking behaviour. How Food Security behaviour is relevant to the investigators? What tools will be used for Food Security.

v) Informed Consent Form: Mobile number of the PI or his representative should be given in the informed consent form so that potential study participants can contact when needed. Direct phone number (9827084) IRB Coordinator should be given instead of PABX number. The mentioned PABX number is also not right.

You are, therefore, advised to address each of the above mentioned observations of the committee and submit the revised version of the protocol for consideration by the chair.

Thank you once again.

Cc: Senior Director, MCHD.

Memorandum

25 April, 2018

To:

Chairperson

Research Review Committee (RRC)

From : Sheikh Jamal Hossain

Principal Investigator of research protocol # PR-18035

Maternal and Child Health Division

Sub: Exemption of external review for Research protocol # PR-18035

Dear Sir,

With due respect, here we are sending our research protocol # PR-18035 on "Evaluating effect of integrating ECD activities into Bangladesh government's urban lactating mothers allowance program for the poor on children's cognition and behaviour" for your exemption of external review. This protocol was reviewed by Health Research Institute, Canada and then was awarded by Grand Challenges Canada (GCC) for two years. If this study works in urban settings of Bangladesh, we will get a new platform for Early Childhood Development service delivery for urban poor children.

Thank you.

Memorandum

25 April, 2018

To:

Chairperson

Research Review Committee (RRC)

From : Sheikh Jamal Hossain

Principal Investigator of research protocol # PR-18035

Maternal and Child Health Division

Sub: Approval of Research protocol # PR-18035

Dear Sir,

With due respect, here we are sending our research protocol # PR-18035 on “Evaluating effect of integrating ECD activities into Bangladesh government’s urban lactating mothers allowance program for the poor on children’s cognition and behaviour” for your review and kind approval. Please noted that this protocol has been awarded by Grand Challenge Canada (GCC) for two years (2018-2020) will be implemented in urban settings of Bangladesh. If this study works in urban settings of Bangladesh, we will get a new platform for Early Childhood Development service delivery for urban poor children.

Thank you.

icddr,b

RRC APPLICATION FORM

RESEARCH PROTOCOL
Number: PR-18035
Version No. 0.05
Version date: 14-07-2019

FOR OFFICE USE ONLY

RRC Approval:	☒ Yes	☐ No	Date:20 May, 2018
ERC Approval:	☐ Yes	☐ No	Date:1 July, 2018
AEEC Approval:	☐ Yes	☐ No	Date:
External IRB Approval	☐ Yes	☐ No	Date:

Name of External IRB: ____________________

Protocol Title:* (maximum 250 characters including space) **Evaluating effects of integrating ECD activities into Bangladesh Government’s urban lactating mothers allowance program for the poor on children’s cognition and behaviour in Rangpur city corporation, Bangladesh**

Short Title: (maximum **100** characters including space) **Urban Lactating Allowance and Psychosocial stimulation (UnLAPS)**

Key Words:* Bangladesh, Behaviour, child, cognition, Psychosocial stimulation, Poor, Urban lactating allowance

Name of the Research Division Hosting the Protocol:*

☐ Health Systems and Population Studies Division (HSPSD)	☒ Maternal and Child Health Division (MCHD)
☐ Nutrition and Clinical Services Division (NCSD)	☐ Laboratory Sciences and Services Division (LSSD)
☐ Infectious Diseases Division (IDD)	☐ Other (specify) ______________________

Has the Protocol been Derived from an Activity:* ☒ No ☐ Yes (please provide following information):

Activity No. :

Activity Title:

PI:

Grant No.: Budget Code: Start Date: 2018 End Date: 2020

icddr,b Strategic Priority/ Initiative (SP 2015-8):* (check all that apply)

☒ Reducing maternal and neonatal mortality	☒ Achieving universal health coverage
☐ Controlling enteric and respiratory infections	☐ Examining the health consequences of climate change
☒ Preventing and treating maternal and childhood malnutrition	☐ Preventing and treating non-communicable diseases
☐ Detecting and controlling emerging and re-emerging infections	☐ Others (specify) improve child cognition and behaviour

Research Phase (4 Ds):* (check all that apply)

☒ Discovery	☒ Delivery
☐ Development	☒ Evaluation of Delivery

Anticipated Impact of Research:* (check all that apply and please provide details below)

☒ Knowledge Production	☒ Informing Policy
☐ Capacity Building	☐ Health and Health Sector Benefits
	☒ Economic Benefits

Please provie details here: The mothers will be able to receive knowledge about proper parenting. The policy will get information about a new plat form to deliver ECD activites for poor urban children. The lactating allowance may help economic benefit for health**.**

Which of the Sustainable Development Goal This Protocol Relates to?:* (check all that apply)

☒ 1. End poverty in all its forms everywhere
☒ 2. End hunger, achieve food security and improved nutrition and promote sustainable agriculture
☒ 3. Ensure healthy lives and promote well-being for all at all ages
☐ 4. Ensure inclusive and equitable quality education and promote lifelong learning opportunities for all
☒ 5. Achieve gender equality and empower all women and girls
☐ 6. Ensure availability and sustainable management of water and sanitation for all
☐ 7. Ensure access to affordable, reliable, sustainable and modern energy for all
☐ 8. Promote sustained, inclusive and sustainable economic growth, full and productive employment and decent work for all
☐ 9. Build resilient infrastructure, promote inclusive and sustainable industrialization and foster innovation
☒ 10. Reduce inequality within and among countries
☐ 11. Make cities and human settlements inclusive, safe, resilient and sustainable
☐ 12. Ensure sustainable consumption and production patterns
☐ 13. Take urgent action to combat climate change and its impacts
☐ 14. Conserve and sustainably use the oceans, seas and marine resources for sustainable development
☐ 15. Protect, restore and promote sustainable use of terrestrial ecosystems, sustainably manage forests, combat desertification, and halt and reverse land degradation and halt biodiversity loss
☐ 16. Promote peaceful and inclusive societies for sustainable development, provide access to justice for all and build effective, accountable and inclusive institutions at all levels
☐ 17. Strengthen the means of implementation and revitalize the global partnership for sustainable development

**Does this Protocol Use the Gender Framework:*** (Please visit: http://shetu.icddrb.org/index.php?option=com_content&view=article&id=265&Itemid=677 for Gender Alanysis Tool with instructions)	☒ Yes (please complete Gender Analysis Tool)<br>☐ No

If 'no' is the response, its reason(s) in brief:

**Will this Research Specifically Benefit the Disadvantaged (**economically, socially and/or otherwise**):**	☒ Yes<br>☐ No
**Does this Protocol use Behaviour Change Communication:**	☒ Yes<br>☐ No
**Principal Investigator (Should be icddr,b staff):*** Sex ☐ Female ☒ Male<br>**Sheikh Jamal Hossain**<br>(Position, phone no, extension no, cell, and email address**):**<br>Senior Research Investigator, cell: 01712191414 Email: sheikh.jamal@icddrb.org<br>Do you have ethics certification? ☐ No ☒ Yes (please attach in your CV below)<br>Do you have RBM training certification? ☐ No ☒ Yes (please attach the certificate with CV below)	Primary Scientific Division of the PI:<br>MCHD
**Co-Principal Investigator(s) Internal**: Sex ☒ Female ☐ Male<br>**Dr. Jena D Hamadani**<br>(Position, phone no, extension no, cell, and email address **):**<br>Scientist, email:jena@icddrb.org<br><br>Signature or written consent of Co-PI: ___________________<br>(electronic signature or email or any sort of written consent)<br>[if more than one, please copy and paste this row for additional Co-PIs]<br><br>Do you have ethics certification? ☐ No ☒ Yes (please attach in your CV below)<br>Do you have RBM training certification? ☐ No ☐ Yes (please attach the certificate with CV below)	Primary Scientific Division/ Programme of the Co-PI<br>MCHD<br><br>_____________________________<br>Approval of the Respective Senior Director/ Programme Head<br><br>(Signature)
**Co-Principal Investigator(s) - External:** Sex ☐ Female ☐ Male<br><br>Address (provide full official address, including land phone no(s), extension no. (if any), cell phone number, and email address).<br><br>Signature or written consent of Co-PI: ___________________<br>(electronic signature or email or any sort of written consent)<br>[if more than one, please copy and paste this row for additional Co-PIs]	
**Co-Investigator(s) - Internal:** Sex ☒ Female ☐ Male<br>**Dr. Fahmida Tofail**<br>(Position, phone no, extension no, cell, and email address **):**<br>Scientist, Email: ftofail@icddrb.org<br><br>Signature or written consent of Co-I: ___________________<br>(electronic signature or email or any sort of written consent)<br>[if more than one, please copy and paste this row for additional Co-Is<br><br>Do you have ethics certification? ☐ No ☒ Yes (please attach in your CV below)<br>Do you have RBM training certification? ☐ No ☐ Yes (please attach the certificate with CV below)	Primary Scientific Division of the Co-I<br>NCSD<br><br>_____________________________<br>Approval of the Respective Senior Director/ Programme Head<br><br>(Signature)

**Co-Investigator(s) - Internal:** Sex ☐ Female ☒ Male<br>**Mohiuddin Ahsanul Kabir Chowdhury** (Position, phone no, extension no, cell, and email address **):**<br>SRI, Email Mohiuddin.Chowdhury@icddrb.org<br><br>Signature or written consent of Co-I: __________________<br>(electronic signature or email or any sort of written consent)<br>[if more than one, please copy and paste this row for additional Co-Is<br><br>Do you have ethics certification? ☐ No ☒ Yes (please attach in your CV below)<br>Do you have RBM training certification? ☐ No ☒ Yes (please attach the certificate with CV below)	Primary Scientific Division of the Co-I: MCHD<br><br>____________________________<br>Approval of the Respective Senior Director/ Programme Head<br><br>(Signature)
**Co-Investigator(s) - Internal:** Sex ☒ Female ☐ Male<br>**Dr. Mohammad Imrul Hasan**<br>(Position, phone no, extension no, cell, and email address **):**<br>SRI, Email: imrul@icddrb.org<br><br>Signature or written consent of Co-I: __________________<br>(electronic signature or email or any sort of written consent)<br>[if more than one, please copy and paste this row for additional Co-Is<br><br>Do you have ethics certification? ☐ No ☒ Yes (please attach in your CV below)<br>Do you have RBM training certification? ☐ No ☒ Yes (please attach the certificate with CV below)	Primary Scientific Division of the Co-I: MCHD<br><br>____________________________<br>Approval of the Respective Senior Director/ Programme Head<br><br>(Signature)
**Co-Investigator(s) - Internal:** Sex ☒ Female ☐ Male<br>**Ms. Fardina Mehrin**<br>(Position, phone no, extension no, cell, and email address **):**<br>RI, Email: fardina.mehrin@icddrb.org<br><br>Signature or written consent of Co-I: __________________<br>(electronic signature or email or any sort of written consent)<br>[if more than one, please copy and paste this row for additional Co-Is<br><br>Do you have ethics certification? ☐ No ☒ Yes (please attach in your CV below)<br>Do you have RBM training certification? ☐ No ☒ Yes (please attach the certificate with CV below)	Primary Scientific Division of the Co-IMCHD<br><br>____________________________<br>Approval of the Respective Senior Director/ Programme Head<br><br>(Signature)
**Co-Investigator(s) - Internal:** Sex ☒ Female ☐ Male<br>**Sk. Masum Billah, SRI**<br>Masum@icddrb.org<br>(Position, phone no, extension no, cell, and email address **):**<br>RI, Email: fardina.mehrin@icddrb.org<br><br>Signature or written consent of Co-I: __________________<br>(electronic signature or email or any sort of written consent)<br>[if more than one, please copy and paste this row for additional Co-Is<br><br>Do you have ethics certification? ☐ No ☒ Yes (please attach in your CV below)<br>Do you have RBM training certification? ☐ No ☒ Yes (please attach the certificate with CV below)	Primary Scientific Division of the Co-I<br>MCHD<br><br>____________________________<br>Approval of the Respective Senior Director/ Programme Head<br><br>(Signature)

**Co-Investigator(s) - Internal:** Sex ☒ Female ☐ Male<br>**Ms. Shamima Shiraji, RI**<br>Shamima.shiraji@icddrb.org<br>(Position, phone no, extension no, cell, and email address **):**<br>RI, Email: fardina.mehrin@icddrb.org<br><br>Signature or written consent of Co-I: ___________________<br>(electronic signature or email or any sort of written consent)<br>[if more than one, please copy and paste this row for additional Co-Is<br><br>Do you have ethics certification? ☐ No ☒ Yes (please attach in your CV below)<br><br>Do you have RBM training certification? ☐ No ☒ Yes (please attach the certificate with CV below)	Primary Scientific Division of the Co-I<br>MCHD<br><br>_____________________________<br>Approval of the Respective Senior Director/ Programme Head<br><br>(Signature)
**Co-Investigator(s) – External:** Sex ☒ Female ☐ Male<br>**Address** (provide full official address, including land phone no(s), extension no. (if any), cell phone number, and email address):<br>Jane Fisher<br>Professor of Global Health, Head of Division of Social Sciences,<br>Public health and preventive medicine<br>Monash University<br>Australia<br>Jane.fisher@monash.edu<br><br>Signature or written consent of Co-I: ___________________<br>(electronic signature or email or any sort of written consent)<br>[if more than one, please copy and paste this row for additional Co-Is]<br><br>**Co-Investigator(s) – External:** Sex ☐ Female ☒ Male<br>**Address** (provide full official address, including land phone no(s), extension no. (if any), cell phone number, and email address):<br>**Dr. Thach Tran**<br>Research Fellow<br>Public health and preventive medicine<br>Monash University<br>Australia<br>Thach.tran@monash.edu<br><br>Signature or written consent of Co-I: ___________________<br>(electronic signature or email or any sort of written consent)<br>[if more than one, please copy and paste this row for additional Co-Is]	
**Student Investigator(s) - Internal:** Sex ☐ Female ☐ Male<br><br>(Position, phone no, extension no, cell, and email address **):**<br><br>Signature or written consent of Student Investitor: ___________________<br>(electronic signature or email or any sort of written consent)<br><br>Have ethics certificate? ☐ No ☐ Yes (If Yes, please attach to your CV below)	Students Affiliation<br><br>_____________________________<br>Approval of the Respective Senior Director/ Programme Head<br><br>(Signature)
**Student Investigator(s) - External:** Sex ☐ Female ☐ Male<br>Address (provide full official address, including land phone no(s), extension no. (if any), cell phone number, and email address):<br><br>Signature or written consent of Student Investitor: ___________________<br>(electronic signature or email or any sort of written consent)	

Collaborating Institute(s): Please provide full official address

Institution # 1

Country	Australia
Contact person	Professor Jane Fisher
Department (including Division, Centre, Unit)	Global Health, School of Social Science, Faculty of Preventive Medicne and Public Health
Institution (with official address)	Monash University, Melbourne Australia.
Directorate (in case of GoB i.e. DGHS)	
Ministry (in case of GoB)	

Institution # 2

Country	
Contact person	
Department (including Division, Centre, Unit)	
Institution (with official address)	
Directorate (in case of GoB i.e. DGHS)	
Ministry (in case of GoB)	

Institution # 3

Country	
Contact person	
Department (including Division, Centre, Unit)	
Institution (with official address)	
Directorate (in case of GoB i.e. DGHS)	
Ministry (in case of GoB)	

Note: If less than or more than three collaborating institutions, please delete or insert blocks as needed.

Contribution by the Members of the Scientific Team:

Members' Name	Contribution								
	Research idea/ concept	Study design	Protocol writing	Respond to external reviewers' comments	Defending at IRB	Developing data collection Tool(s)	Data Collection	Data analysis/ interpretation of results	Manuscript writing
Sheikh Jamal Hossain	☒	☒	☒	☒	☒	☒	☒	☒	☒
Dr. Jena Hamadani	☒	☒	☒	☒	☒	☒	☒	☒	☒
Dr. Fahmida Tofail	☐	☒	☒	☒	☒	☒	☐	☒	☒
Dr. Mohiuddin Ahsanul Kabir Chowdhury	☐	☒	☒	☒	☒	☒	☐	☒	☒
Dr. Mohammad Imrul Hasan	☐	☐	☐	☐	☒	☒	☐	☒	☒
Ms. Fardina Mehrin	☐	☐	☐	☐	☒	☒	☐	☒	☒
Sk. Masum Billah	☐	☒	☐	☐	☒	☒	☐	☒	☒
Ms. Shamima Shiraji	☐	☐	☐	☐	☒	☒	☒	☒	☐
Professor Jane Fisher	☐	☐	☐	☐	☐	☐	☐	☒	☒
Dr Thach Tran	☐	☐	☐	☐	☐	☐	☒	☒	☒
	☐	☐	☐	☐	☐	☐	☐	☐	☐
	☐	☐	☐	☐	☐	☐	☐	☐	☐
	☐	☐	☐	☐	☐	☐	☐	☐	☐
	☐	☐	☐	☐	☐	☐	☐	☐	☐

Study Population: Sex, Age, Special Group and Ethnicity

Research Subject:
☒ Human
☐ Animal
☐ Microorganism
☐ Other (specify): ______________

Sex:
☒ Male
☒ Female
☐ Transgender

Age:
☒ 0 – 4 years
☐ 5 – 10 years
☒ 11 – 17 years
☒ 18 – 64 years
☐ 65 +

Special Group:
☐ Pregnant Women
☐ Fetuses
☐ Prisoners
☐ Destitutes
☐ Service Providers
☐ Cognitively Impaired
☐ CSW
☐ Expatriates
☐ Immigrants
☐ Refugee
☐ Others (specify): Poor lactating mothers and children

Ethnicity:
☒ No ethnic selection (Bangladeshi)
☐ Bangalee
☐ Tribal group
☐ Other (specify): _________________

NOTE: It is icddr.b's policy to include men, women, children and transgender in its research projects involving participation of humans, unless there is strong justification(s) for their exclusion.

Consent Process: (Check all that apply)

☒ Written
☐ Oral
☐ Audio
☐ Video
☐ None

Language:

☒ Bangla
☐ English
☐ Other (specify: ________________

Project/Study Site: (Check all that apply)

☐ Chakaria
☐ Bandarban
☐ Dhaka Hospital
☐ Kamalapur Field Site/HDSS
☐ Mirpur (Dhaka)
☐ Matlab DSS Area
☐ Matlab non-DSS Area
☐ Matlab Hospital
☐ Mirzapur
☐ Bianibazar (Sylhet)
☐ Kanaighat (Sylhet)
☐ Jakigonj (Sylhet)
☐ Other community in Dhaka
Name: ______________________________
☒ Other sites in Bangladesh
Name: Rangpur City corporation
☐ Multi-national Study
Name of the country____________________

Project/Study Type: (Check all that apply)

☐ Case Control Study
☐ Clinical Trial (Hospital/Clinic/Field)*
☒ Community-based Trial/Intervention
☐ Cross Sectional Survey
☐ Family Follow-up Study
☐ Longitudinal Study (cohort or follow-up)
☐ Meta-analysis
☐ Programme Evaluation
☐ Programme (Umbrella Project)
☐ Prophylactic Trial
☐ Record Review
☐ Secondary Data Analysis
Protocol No. of Data Source: ____________
☐ Surveillance/Monitoring
☐ Systematic Review
☐ Other (specify): ______________________

***Note:** International Committee of Medical Journal Editors (ICMJE) defines Clinical Trial as "*Any research project that prospectively assigns human participants to intervention and comparison groups to study the cause-and-effect relationship between a medical intervention and a health outcome*".

PI of the RRC- and ERC-approved Clinical Trials should provide necessary information to IRB Secretariat (Research Administration) for registration and uploading into relevant websites (usually at the https://register.clinicaltrials.gov/). They should also provide relevant information to the IRB Secretariat in the event of amendment/modification after their approval by RRC and ERC.

Biological Specimen:

a) Will the biological specimen be stored for future use?	☐ Yes ☐ No ☒ Not applicable
b) If the response is 'yes', how long the specimens will be preserved?	__________ years
c) What types of tests will be carried out with the preserved specimens?	
d) Will the consent be obtained from the study participants for use of the preserved specimen for other initiative(s) unrelated to this study, without their re-consent?	☐ Yes ☐ No ☒ Not applicable
e) Will the specimens be shipped to other country/ countries? If yes, name of institution(s) and country/countries.	☐ Yes ☐ No ☒ Not applicable<br>Name ______________________________
f) If shipped to another country, will the surplus/unused specimen be returned to icddr,b? If the response is 'no', then the surplus/unused specimen must be destroyed.	☐ Yes ☐ No ☒ Not applicable
g) Who will be the custodian of the specimen at icddr,b?	
h) Who will be the custodian of the specimen when shipped outside Bangladesh?	
i) Who will be the owner(s) of the specimens?	
j) Has a MoU been signed with regards to collection, storage, use and ownership of specimen? If the response is 'yes', please attach a copy of the MoU.. If the response is 'no', appropriate justification should be provided for not signing a MoU.	☐ Yes ☐ No ☒ Not applicable

Proposed Sample Size:
Sub-group (Name of subgroup e.g. Men, Women) and Number

Name	Number	Name	Number
(1) Lactating allowance + Psychosocial stimulation	300		
(2) Only Lactating Allowance	300		
		Total sample size	600

Determination of Risk: Does the Research Involve (Check all that apply)

☐ Human exposure to radioactive agents?
☐ Foetal tissue or abortus?
☐ Investigational new device?
Specify: ________________
☐ Existing data available from Co-investigator?
☐ Human exposure to infectious agents?
☐ Investigational new drug?
☐ Existing data available via public archives/sources?
☐ Pathological or diagnostic clinical specimen only?
☒ Observation of public behaviour?
☐ New treatment regime?

	Yes	No
Will the information be recorded in such a manner that study participants can be identified from the information directly or through identifiers linked to the study participants?	☐	☒
Does the research deal with sensitive aspects of the study participants' sexual behaviour, alcohol use or illegal conduct such as drug use?	☐	☒

Could information on study participants, if available to people outside of the research team:

	Yes	No
a) Place them at risk of criminal or civil liability?	☐	☒
b) Damage their financial standing, reputation or employability, or social rejection, or lead to stigma, divorce etc.?	☐	☒

Do you consider this research: (check one)

☐ Greater than minimal risk	☒ No more than minimal risk	☐ Only part of the diagnostic test

Note: Minimal Risk: The probability and the magnitude of the anticipated harm or discomfort to participants is not greater than those ordinarily encountered in daily life or during the performance of routine physical, psychological examinations or tests, e.g. the risk of drawing a small amount of blood from a healthy individual for research purposes is no greater than when the same is performed for routine management of patients.

**Risk Group of Infectious Agent and Use of Recombinant DNA**				
a) Will specimens containing infectious agent be collected?	☐ Yes	☐ No	☒ Not applicable	
b) Will the study involve amplification by culture of infectious agents?	☐ Yes	☐ No	☒ Not applicable	
c) If response to questions (a) and/or (b) is 'yes', to which Risk Group (RG) does the agent(s) belong? (Please visit http://shetu.icddrb.org/index.php?option=com_content&view=article&id=265&Itemid=677 to review list of microorganism by Risk Group)	☐ RG1	☐ RG2	☐ RG3	☐ RG4
d) Does the study involve experiments with recombinant DNA?	☐ Yes	☐ No	☒ Not applicable	

Does the study involve any biohazards materials/agents or microorganisms of risk group 2, 3, or 4 (GR2, GR-3 or GR4)?

☐ Yes ☒ No

[If the response is 'yes'] I, (print name of the PI) affirm that we will use the standard icddr,b laboratory procedures for biosafety of the hazardous materials/agents or microorganisms in the conduction of the study.

________________________ ________

Signature of the Principal Investigator **Date**

Dissemination Plan: [please explicitly describe the plans for dissemination, including how the research findings would be shared with stakeholders, identifying them if known, and the mechanism to be used; anticipated type of publication (working papers, internal (institutional) publication, international publications, international conferences/seminars/workshops/ agencies. [Check all that are applicable]

**Dissemination type**	**Response**		**Description (if the response is a yes)**
Seminar for icddr,b scientists/ staff	☐ No	☒ Yes	The investigators at the conclusion of the trial, to explain the results and outcomes of the trial.
Internal publication	☐ No	☒ Yes	We will publish in icddr'b health bulletin
Working paper	☐ No	☐ Yes	
Sharing with Government of Bangladesh (GoB)	☐ No	☒ Yes	We will share with relevant government department e.g Ministry of Women and Children Affairs (MOWCA), Ministry of Health and Family Welfare (MOH&FW), Ministry of Local Government and Rural Development and Cooperatives (MOLRD&C)
Sharing with national Non Government Organizations (NGOs)	☐ No	☒ Yes	We will submit our work for presentation at national workshop/ seminars which focus on child development
Presentation at national workshop/ seminar	☐ No	☒ Yes	We will submit our work for presentation at leading international conferences.
Presentation at international workshop/ conference	☐ No	☒ Yes	We will present in national conferences and workshop
Peer-reviewed publication	☐ No	☒ Yes	We will do peer review publication
Sharing with international agencies	☐ No	☒ Yes	As above, we will prepare a policy brief for sharing with international donors. We will also be available to discuss the results in person and at workshops.
Sharing with donors	☐ No	☒ Yes	
Policy brief	☐ No	☒ Yes	As above, we will prepare a policy brief for sharing with international agencies. We will also be available to discuss the results in person and at workshops.
Other			
Other			

Funding:

Is the protocol fully funded?	☒ Yes	☐ No
If the answer is yes, please provide sponsor(s)'s name	1. Grand Challenges Canada (GCC)	
	2.	
Is the protocol partially funded?	☐ Yes	☒ No
If the answer is yes, please provide sponsor(s)'s name	1.	
	2.	

If fund has not been identified:

Is the proposal being submitted for funding?	☐ Yes	☒ No
If yes, name of the funding agency	1.	
	2.	

Conflict of interest:

Do any of the participating investigators and/or member(s) of their immediate families have an equity relationship (e.g. stockholder) with the sponsor of the project or manufacturer and/or owner of the test product or device to be studied or serve as a consultant to any of the above?

☐ No ☐ Yes (please submit a written statement of disclosure to the Executive Director, icddr,b)

Proposed Budget:

Dates of Proposed Period of Support

(Day, Month, Year - DD/MM/YY)

Beginning Date : June, 2018

End Date : May, 2020

Cost Required for the Budget Period ($)

Years	Direct Cost	Indirect Cost	Total Cost
**Year-1**	91,376	11918	103293
**Year-2**	79,639	13927	93558
**Year-3**			
**Year-4**			
**Year-5**			
**Total**	171015	25845	196850

Certification by the Principal Investigator:

I certify that the statements herein are true, complete and accurate to the best of my knowledge. I am aware that any false, fictitious, or fraudulent statements or claims may subject me to criminal, civil, or administrative penalties. I agree to accept the responsibility for the scientific conduct of the project and to provide the required progress reports including updating protocol information in the NAVISION if a grant is awarded as a result of this application.

I also certify that I have read icddr,b Data Policies and understand the PIs' responsibilities related to archival and sharing of research data, and will remain fully compliant to the Policies. (Note: The Data Policies can be found here: http://shetu.icddrb.org/index.php?option=com_content&view=article&id=273&Itemid=685)

______________ ______________

Signature of PI **Date**

Approval of the Project by the Division Director of the Applicant:

The above-mentioned project has been discussed and reviewed at the Division level.

Dr. Shams El Arifeen ______________

Name of the Division Director Signature Date of Approval

Table of Contents

RRC APPLICATION FORM 16
Project Summary 282
Hypothesis to be tested: 294
Specific Objectives: 294
Background of the Project including Preliminary Observations: 294
Research Design and Methods 316
Sample Size Calculation and Outcome (Primary and Secondary) Variable(s) 20
Data Analysis 20
Data Safety Monitoring Plan (DSMP) 21
Ethical Assurance for Protection of Human rights 21
Use of Animals 21
Collaborative Arrangements 22
Facilities Available 22
Literature Cited 37
Budget 38
Other Support 39
Biography of the Investigators 39
Format for Consent Form 36
Check-List 92

☒ Check here if appendix is included

Project Summary

[The summary, within a word limit of 300, should be stand alone and be fully understandable.]

Principal Investigator: Sheikh Jamal Hossain
Research Protocol Title: Evaluating effects of integrating ECD activities into Bangladesh Government's urban lactating mothers allowance program for the poor on children's cognition and behaviour in Rangpur City corporation, Bangladesh
Proposed start date: May 2018   Estimated end date: April 2020

Background (brief):

a. Burden:

Provision of health care to urban poor population is a great challenge because of supply and demand barriers in the urban health system, which is considered patchy and fragmented. The poor urban people, especially mothers have little access to government health facilities. The health care platform is not well designed in primary health care delivery for urban health system but presently almost 30 percent people are living in urban area of the country.

b. Knowledge gap:

Little is known about what happen if psychosocial stimulation is provided using urban lactating allowance program on children's cognition and behaviour. We also do not know nature and bottleneck of Early Childhood Development (ECD) activities available in the urban Bangladesh.

c. Relevance:

This is an opportunity to develop a combined package integrating psychosocial stimulation with the existing urban lactating allowance program on disadvantaged children'sdevelopment. As the urban health system is complex, patchy and fragmented, prior to our intervention we will analyze ECD services and its bottleneck in the project area through Tanahashi framework.

Hypothesis (if any):

Adding psychosocial stimulation to urban lactating allowance program will have additional effect on children's cognitive, motor and language development and behaviour compared to the comparsion group

Secondary Hypothesis:

Additionally the intervention will-

improve family care environment (e.g. father involvement), mothers' knowledge, quality of life and reduce their depressive symptoms and violence against mother

be cost effective,

Objectives:

-To evaluate the effect of integrated urban lactating allowance and psychosocial stimulation on children's cognitive, motor and language development and behaviour

-To measure nature and bottleneck of ECD services in urban Bangladesh

Secondary objectives:

To measure effect of the programmes on:

-mothers' quality of life and mental health (depression symptoms), violence against mother, stimulation environment (e.g. father involvement), mother knowledge on child care.

-cost effectiveness of the intervention

Methods: A two-arm, Cluster Randomized Controlled Trial: i) Lactating allowance + Psychosocial stimulation; (ii) Only lactating allowance

Outcome measures/variables: Childs's cognitive, motor and language development measured on Bayley-III, behaviour on Wolke's rating scales, Mother's quality of life and depressive symptoms and violence against mothers, household food security status, socioeconomic status, quality of home stimulation using family care indicators, Mother's knowledge on child care and development, children's growth measured by length/height, weight and head circumference, direct and indirect cost.

Description of the Research Project

Hypothesis to be tested:

In a hypothesis testing research proposal, briefly mention the hypothesis to be tested and provide the scientific basis of the hypothesis, critically examining the observations leading to the formulation of the hypothesis.

Does this research proposal involve testing of hypothesis: ☐ No ☒ Yes (describe below)

Adding psychosocial stimulation to urban lactating allowance program will improve children's cognitive, motor and language development and behaviour compared to the comparison group

Specific Objectives:

Describe the specific objectives of the proposed study. State the specific parameters, gender aspects, biological functions, rates, and processes that will be assessed by specific methods.

Primary objectives:

To evaluate the effect of adding psychosocial stimulation to existing urban lactating allowance on children's cognitive, motor and language development and behaviour

To measure nature and bottleneck of ECD services in urban Bangladesh

Secondary objectives:

To measure effect of adding psychosocial stimulation to existing urban lactating allowance on:

-mothers' quality of life, violence agaist mothers and mental health (depression symptoms), stimulation environment (fathers' involvement)
-cost effectiveness of the intervention

Background of the Project including Preliminary Observations:

Provide scientific validity of the hypothesis based on background information of the proposed study and discuss previous works on the research topic, including information on sex, gender and diversity (ethnicity, SES) by citing specific references. Critically analyze available knowledge and discuss the questions and gaps in the knowledge that need to be filled to achieve the proposed aims. If there is no sufficient information on the subject, indicate the need to develop new knowledge.

Background:

It is estimated that 250 million children do not reach their potential due to poverty, inadequate stimulation and associated risk factors in low and middle income countries(1) .
In Bangladesh more than 50% of children under 5 years are at risk of developmental delay (2). Urban population is about 28% and it is expected to rise to more than 40% by 2030 (3). In fact, urbanization in

Bangladesh is rapidly growing but is not well planned (4). Poor urban women and children are vulnerable in terms of health and education (5) .

The urban health care facilities are fragmented and patchy and every year rise in population is putting more pressure on health system. Moreover, there is huge economic disparity between rich and poor in the urban areas and poorer people have little access to health care due to both supply and demand barriers. So the situation is rolling more difficult every day.

Health care is provided by two different ministries like Ministry of Health and Family Welfare (MOHFW) and Ministry of Local Government and Rural Development & Cooperatives (MOLGRDC) in the urban areas.Recently, Ministry of Women and Children Affairs (MOWCA) has also taken initiatives to help urban poor mothers through their lactating allowance (LA) and limited health education programs. The mothers receive the LA through banking system directly. Conditional and unconditional cash (allowance) transfer to the poor mothers has proven to reduce poverty and improve health outcomes for mothers and children worldwide (6-10). A study conducted in Zimbabwe documented that unconditional cash transfer showed an increase in receiving children’s birth certificate and imuunization in the age group of 0-4years and school attendance in 6-12 years (11). Recent systematic review concluded that unconditional cash transfer may not improve health services use in children and adult in low and middle income coutries but may have positive impact on food choices and food security (12). We hope that this urban lactating allowance will develop economic capacity of people, empower women, improve their quality of life and reduce gender gap. Our psychosocial stimulation will add to mothers’ capacity for child care and developmental practices as reported previously (13).

Very few ECD programs are available for poor urban children especially for the 1st thousand days. Our idea is to develop a combined package integrating psychosocial stimulation with the existing urban lactating allowance program and measuring its synergistic effects on disadvantaged children’s development.

As the urban health system is complex, patchy and fragmented (14), prior to our intervention we will analyze ECD services and its bottleneck in the project area through Tanahashi framework. We will also conduct facility assessment to understand nature of ECD services. Then we will take decision for service delivery vehicle (Who, when, where, how etc.) through workshop with stakeholders. We hope to identify a sustainable delivery method with little additional expenses. So, this will be an integrated, cost effective and scalable program to improve development of children of poor young mothers in urban Bangladesh which has not been tried previously in this population. Therefore, if successful, this program will help the country in achieving Sustainable Development goals (SDGs) No. 3, 4 & 5.

Government of Bangladesh (GoB) has developed health policy and national health policy in 2011 emphasizing health care for the disadvantaged and the poor. Recently GOB has also developed urban health strategy to focus health of urban people. It is implementing fifth Health, Population and Nutrition Sector Program (HPNSP) 2017-2021 for ensuring health and nutrition of the people giving priority to the vulnerable population. MoWCA revised ‘the National Child Policy 1994’ in 2011. The first aim and objective of ‘the National Children Policy 2011’ is to ensure optimum child development and growth of the children in the country irrespective of any economic, social, gender and geographical barriers (15). Our present study is completely aligned with the Children Policy 2011, Bangladesh Health policy, Bangladesh urban health strategy and Health Population Nutrition Sector Program (HPNSP) 2017-2021. We expect this study, if successful, will have an influence on sustainable Development Goals (SDG) of Bangladesh in the field of children’s health and development if scaled throughout the nation. In addition maternal health is also one of the priorities of the GoB and our innovation will help improve maternal physical, mental and emotional well-being.

Research Design and Methods

Describe the research design and methods and procedures to be used in achieving the specific aims of the research project. If applicable, mention the type of personal protective equipment (PPE), use of aerosol confinement, and the need for the use BSL2 or BSL3 laboratory for different part of the intended research in the methods.. Define the study population with inclusion and exclusion criteria, the sampling design, list the important outcome and exposure variables, describe the data collection methods/tools, and include any follow-up plans if applicable. Justify the scientific validity of the methodological approach (biomedical, social, gender, or environmental).

Also, discuss the limitations and difficulties of the proposed procedures and sufficiently justify the use of them.

This study will follow two sequential stages:

Stage I: Understand nature and analyse bottleneck of ECD services in selected urban areas of Bangladesh through a survey using Tanahasi framework.

Stage II: Document that adding ECD activities to urban lactating allowance program will improve children's cognition, motor, language and behaviour.

Methodology for stage I:

First, we will analyze ECD program in urban area through Tanahashi framework. We will document availability, accessibility, utilization and adequate coverage of the ECD activities.

We will analyze ECD and related services through:

a) Household Survey and
b) Facility assessment

a) Household survey

We will conduct a survey with 426 mothers having child less than five years from two pouroshova/municipalities. On an average 22 respondents will be recruited from each of our cluster. We have 20 clusters for our interventional study (stage ii). First respondent of each cluster will be selected based on EPI coverage survey and then after every five household will be selected to fill 22 respondents in each cluster.

Sample size was calculated assuming 50% effective coverage, 20% error, 95% confidence interval and 10% refusal rate the total sample size calculated was 426 in each pouroshova.

b) Facility Assessment

Moreover, we will visit different facilities providing ECD or relevant services. We will collect facilities working for ECD and related services from local government elected authorities, MOWCA deployed personnel, NGO service delivery Program (NSDP) personnel or other NGO services personnel and then will pick convenient number of of services cenrter randomly from the pouroshova.

We will use two check lists; one for facility assessment another for household survey. The latest Lancet series of child development accumulate the services relavant for child development(16). We will develop the checklist based on that lancet article. We will collect information of availability of services facility assessment and information of accessibility, utilization and coverage will be collected from interview of mother child dyads. With this quantitative result, we will conduct at least two discussion sessions with ECD experts, Urban health expert, urban health program managers, district/division officer of MOWCA, pre primary education experts, and others GoB and non GoB stakeholders to conclude decision on –who (type of health workers) will provide ECD activities, when the ECD will be delivered (participant's time), How (group or individual) the delivery will be conducted, where would be the delivery point (domiciliary or facility) etc. We aim to do

this primary study so that we can find a good platform for smooth and effective delivery of our integrated ECD packages.

Methodology for (RCT) stage II:

Our main aim is to document that adding ECD activities to urban lactating allowance program improves children's cognition, language and behaviour during the first thousand days.

Study design:
This is a cluster randomized controlled trial of the effects of adding psychosocial stimulation to the existing lactating allowance of the country with 2 arms: i) Psychosocial stimulation+ Urban lactating allowance and ii) Only urban lactating allowance.

Selection of the study area:

We will select pouroshovas/municipalities required for our study. Initially we are planning to conduct the study in Rangpur City Corporation.

Recruitment:
We will require 20 clusters (10 interventions & 10 comparisons) and 30 mother child dyads in each cluster. In Rangpure City Corporation there are 33 Wards. A Ward will be considered as a cluster. Cluster will be assigned randomly to intervention or comparisions group. We will create a buffer zone to reduce spill over effect with remaining Wards and or villages.

As the Government is providing urban lactating allowance to 2000 mothers in Rangpur City Corporation, we are expecting approximately at least 50 mother child dyads in each cluster. We will list all mothers and then we will recruit 30 participants considering mothers age <35 years and/or those with single child.

Disabled mothers (if any) will be considered for all intervention but we will not be included in data analysis.

Some other eligibility criteria for this study:
Children will be assigned to the study only if they meet all of the inclusion criteria and none of the exclusion criteria.

Inclusion criteria:

- Mothers with a child aged 6-16 months
    -
- Not expected to leave the study site for more than 2 months
- Has a legally acceptable representative capable of understanding the informed consent document and providing consent on the participant's behalf.

Exclusion criteria:

- Legal guardian unwilling or unable to provide written informed consent.
- Known congenital anomaly, developmental disorder or severe developmental delay
- If not possible to test the child due to physical or behavioural problems
- Children of multiple birth e.g. twin, triplets

Intervention and Project Execution Plan:
The study aims to integrate two distinct interventions: i) Urban lactating allowance and ii) Psychosocial stimulation.

i) Urban lactating allowance:

Directorate General of Women Affairs (DGWA) under the Ministry of Women and Children Affairs (MOWCA) in Bangladesh has started a safety net program for urban poor lactating mothers called lactating allowance program. The eligibility criteria of participants are: poor mothers (mother's income less than 5000BDT and no other source of income); working mothers (including those performing household chores), permanent residence in urban areas, pregnant and lactating mothers, mother's age>20 years, number of children<3. They provide Taka 500 (US$6.3) for each selected mothers every month. In fiscal year 2016-17 there were 240,000 beneficiary mothers in urban area. The beneficiaries also receive minimum level of health education by some local developmental organizations. The objectives of this program is to help pregnant and lactating mothers protect their own and their children's health and wellbeing. Indeed, the GOB program is an integration to urban health system for poor women and children's health as these population have little access to GOB health facilities and the private health care is costly for them. We will add psychosocial stimulation with this GOB program to enhance child development. So this multisectoral integrated approach will benefit quality of life and mental health of the mothers as well as developmental and nutritional status of their children.

iii) ECD activities/Psychosocial stimulation: The participants will receive fortnightly sessions of psychosocial stimulation for one year. The play leader will demonstrate play activities to the mothers using toys made from recycled materials; the toys will be provided to mothers and exchanged with new toys on the following visit.The mothers will be trained on how to interact with the child during play and even during household chore e.g. during bathing, feeding, clothing etc. the mother can teach something to the child. All play and messages will be provided based on child's age. Mother will continue the activities with her child upto next visit by play leader when she will be given new activities. Local rhymes, songs etc. will be taught to mothers to make it easily deliverable to the child. The curriculum that will be used was first developed and used in Jamaica and then a culturally modified and translated version was developed for use in Bangladesh. The curriculum is based on improving the mother-child interaction, and providing developmentally appropriate activities for the child. The curriculum has already been used in several projects in Bangladesh run by Child Development Unit of icddr,b and has shown significant benefits to children's development(17-20) and growth(18, 19).

Training: Data will be collected at baseline and endline of the study. All testers will collect data after an intensive training for one month on the Bayley, behaviour ratings, anthropometry and other data collection tools. When the inter observer reliability coefficient will be more than 0.8, we will consider the trainee to be eligible to take part in the test procedure. Quality of data collection will be checked at the field by supervisors/master trainers on 10% of the assessments.

Play leaders will receive training on the curriculum and will be monitored by the supervisors.
Data will be checked by supervisors every day and will be transferred to icddr,b head office for entry every fortnight. All hard copies will be kept in a locker under PI's supervision. The data will be entered into a personal computer which is password protected.

Team Composition:

We have a team composition of Child development scientists, health economist, Health education expert and psychologists. With the team we have a long experience to work in this field.

Expected Health Outcomes and Outputs:

Children's cognitive, language and motor development, behaviour and growth
Mothers' quality of life, violence status and depressive symptoms and stimulation environment (e.g. father envolvement)
Family food security status and health seeking behaviour

Data collection tools:
All data will be collected from all groups. Data will be collected at baseline and after one year of intervention at end line

Cognition and behavior: Bayley Scales of Infant and Toddler Development-III (Bayley-III) will be used to measure children's cognitive, language and motor development and Wolke's behaviour ratings will be used to measure children's behaviour by testers with a Masters degree in psychology or equal education.

Anthropometric measurements: Weight, length and head circumference of children, weight and height of mothers will be measured by testers just after the Bayley test using standard WHO techniques.

Maternal Quality of Life and Depressive symptoms: Maternal quality of life will be measured by World Health Organization (WHO) Quality of life questionnaire (WHOQoL) and depressive symptom will be measured by Self Reporting Questionnaire (SRQ 20). Household Food Insecurity status wll be measured by Household Food Insecurity Access Scale (HFIAS). Violence gaist mother and falily care indicators. All the above scales have been used in Bangladesh.
Demographic and socio economic status, Health seeking behavior, and women empowerment questionnaire will be used by the field research assistants (FRAs).

Direct and indirect cost information of the intervention will be collected during the last 3 months of study and at the end we will get all direct intervention cost

Quality of data and management:

The main activities of the project are: Cluster randomization, Enrolment of the participants, training of the testers, baseline data collection, intervention, end line data collection, data entry, analysis and report writing. We will ensure quality of the activities in all the steps. Before enrolment we will randomize clusters by a colleague who is not involved in the study. We will recruit a master trainer with previous experiences on Bayley. The master trainer will train all the testers and conduct interobserver reliability measures by observing and independently scoring at least 10 tests by each tester. She will also ensure ongoing quality by observing 10% of the tests in the field. Similarly the FRAs will be trained and monitored by a trainer/supervisor. We will also measure pre and post training knowledge of the participants.

Psychosocial stimulation intervention is the most important part of the project. We will hire a trainer with experience in ECD activities. She will provide extensive training to the play leaders on the curriculum and will supervise their sessions regularly. Personnel will coordinate all activities in the field with the consultation of investigators. Weekly trouble shooting meetings will be held for first two months and any serious problem will be discussed and then again measures will be taken to solve it.Later, monthly meeting will be organized to solve any field problem. The investigators have long experience in this field of research and will ensure smooth running of project activities in every step.

Data Storage
Data will be stored in databases saved on icddr,b servers.The databases will be password protected and only members of the study team and investigators will have access to the password. Only deidentified data will be stored on the database.

Record Retention
Data will be available to the principal investigators. Following the archival period, hard copies of the data will be destroyed according to local procedures. The database will be deleted at this point by the investigators.

Time Line:
Project Preparatory phase: Month 1st to 3rd

Enrollment and baseline assessments: Month 4th to 6th
Intervention: Month 7th to 19th
Endline assessments: Month 20th to 22nd
Data analysis and report writing: Month 22nd to 24th

Sample Size Calculation and Outcome (Primary and Secondary) Variable(s)

Clearly mention your assumptions. List the power and precision desired. Describe the optimal conditions to attain the sample size. Justify the sample size that is deemed sufficient to achieve the specific aims.

In total, sample is 600. In our context, previous studies of psychosocial stimulation alone achieved an effect size of 0.4 SD on children's development. In this study, we have additional cash as an intervention that might have also positive effect on the child development. But to be on the safe side, we assume an effect size of 0.35 SD, 80% power, 95% confidence interval, 1.58 cluster design effect and 25% drop-out rate the sample size was calculated to be 300 in each arm with 30 children in each of the 20 clusters.

$$n = \frac{2(sd)^2}{(M_1-M_2)^2} \times f(\alpha\beta)$$

Design Effect = 1 + [(m-1) * ICC]

n=sample size per group
sd=Standard deviation of population=1
M1-M2= Difference between the groups assumed=0.35
αβ= 80% power and 5% significance
m=Number of mother/child pairs in each cluster=30
ICC=0.02

Data Analysis

Describe plans for data analysis, including stratification by sex, gender and diversity. Indicate whether data will be analysed by the investigators themselves or by other professionals. Specify what statistical software packages will be used and if the study is blinded, when the code will be opened. For clinical trials, indicate if interim data analysis will be required to determine further course of the study.

Stage I: Data will be checked for normality. Appropriate measures will be taken if there are any abnormal distributions. Frequency table and percentage will be used to present nature of ECD services and its bottleneck. Service availability would be presented in a table through facility assessment and the bottlenecks (accessibility, utilization and coverage) will also be presented through interviews with mothers.

Stage II for RCT: For interventional trial, differences between groups in background characteristics will be analyzed using t-test for continuous variables and chi-square test for categorical variables.. Effect of the ECD intervention will be measured using intention-to-treat analysis controlling for the clustering effect in multivariate regression analysis. All covariates will be controlled and mulnicolinearity will be checked before regression analysis. Quality of life data will be analysed based on WHO guideline for WHOQoL tools and then we will compare different domain of quality of life between the arms. Similarly, total score of SRQ will be calculated and then differences between arms will be analysed.

Cost-effectiveness Analysis:
The objective of the study is to estimate the resource use and costs associated with interventions. We will measure direct and indirect cost for all the groups. Average costs per participants will be calculated by multiplying the cost of resource items by their respective unit costs. We will also get the outcomes of the study after the intervention and the differences will be measured before and after the intervention. Then the incremental cost-effectiveness ratio (ICER) will be used in cost-effectiveness analysis.

Data Safety Monitoring Plan (DSMP)

All clinical investigations (research protocols testing biomedical and/or behavioural intervention(s)) should include the Data and Safety Monitoring Plan (DSMP). The purpose of DSMP is to provide a framework for appropriate oversight and monitoring of the conduct of clinical trials to ensure the safety of participants and the validity and integrity of the data. It involves involvement of all investigators in periodic assessments of data quality and timeliness, participant recruitment, accrual and retention, participant risk versus benefit, performance of trial sites, and other factors that can affect study outcome.

N/A

Ethical Assurance for Protection of Human rights

Describe the justifications for conducting this research in human participants. If the study needs observations on sick individuals, provide sufficient reasons for using them. Indicate how participants' rights will be protected, and if there would be benefit or risk to each participants of the study. Discuss the ethical issues related to biomedical and social research for employing special procedures, such as invasive procedures in sick children, use of isotopes or any other hazardous materials, or social questionnaires relating to individual privacy. Discuss procedures safeguarding participants from injuries resulting from study procedures and/or interventions, whether physical, financial or social in nature. [Please see Guidelines]

There is no risk to your child and it will be more like a fun game. However, if there is any obvious disability in your child that concerns you, we will refer him/her to the proper institutions and will also give you a referral card so that you can easily take your child to them.

The tests we are using are not standardized for Bangladeshi children and will only pick up differences between the intervention and control groups. We therefore cannot diagnose delayed IQ and will not report the results to parents.

Obvious disability is defined as any disability that is apparently visible like visual or hearing impairment, limb paralysis, etc. Learning disabilities will be considered if the child falls in the lowest quintile of the tested population. However, parents cannot be notified at the end of the test because we need the entire sample to be assessed before we can assign a child to this group.
For those children who require referral, we will invite their parents to a separate place and will inform them by a clinical psychologist who will disclose the status.

Use of Animals

Describe if and the type and species of animals to be used in the study. Justify with reasons the use of particular animal species in the research and the compliance of the animal ethical guidelines for conducting the proposed procedures.

N/A

Collaborative Arrangements

Describe if this study involves any scientific, administrative, fiscal, or programmatic arrangements with other national or international organizations or individuals. Indicate the nature and extent of collaboration and include a letter of agreement between the applicant or his/her organization and the collaborating organization.

This project will be implemented in collaboration of University of Western Australia and University of Queensland, Australia

Facilities Available

Describe the availability of physical facilities at site of conduction of the study. If applicable, describe the use of Biosafety Level 2 and/or 3 laboratory facilities. For clinical and laboratory-based studies, indicate the provision of hospital and other types of adequate patient care and laboratory support services. Identify the laboratory facilities and major equipment that will be required for the study. For field studies, describe the field area including its size, population, and means of communications plus field management plans specifying gender considerations for community and for research team members.

N/A

Abberiviations:

Bayley-III: Bayley Scales of Infant and Toddler Development-III
BDT: Bangladeshi Taka
DGWA: Directorate General of Women Affairs
FRAs: Field Research Assistants
GoB: Government of Bangladesh
WHO: World Health Organization
WHOQoL: Quality of life questionnaire
HPNSP: Health, Population and Nutrition Sector Program
ICER: incremental cost-effectiveness ratio
MOHFW: Ministry of Health and Family Welfare
MOLGRDC: Ministry of Local Government and Rural Development & Cooperatives
MOWCA: Ministry of Women and Children Affairs
SDGs: Sustainable Development goals
SRQ 20: Self Reported Questionnaire
SD: Standard Deviation

Literature Cited

Identify all cited references to published literature in the text by number in parentheses. List all cited references sequentially as they appear in the text. For unpublished references, provide complete information in the text and do not include them in the list of Literature Cited. There is no page limit for this section, however, exercise judgment in assessing the "standard" length.

2. Hamadani JD, Tofail F, Huda SN, Alam DS, Ridout DA, Attanasio O, et al. Cognitive deficit and poverty in the first 5 years of childhood in Bangladesh. Pediatrics. 2014;134(4):e1001-e8.
3. Bangladesh Bureau of Statistics (BBS) SaID, Ministry of planning. Population Projection of Bangladesh:Dynamics and Trends 2011-2061. 2015.
4. Ellis P, Roberts M. Leveraging urbanization in South Asia: Managing spatial transformation for prosperity and livability: World Bank Publications; 2015.
5. Afsana K, Wahid SS. Health care for poor people in the urban slums of Bangladesh. The Lancet. 2013;382(9910):2049-51.
6. Adato M, Hoddinott J. Conditional cash transfer programs: A" magic bullet" for reducing poverty? 2007.
7. Fiszbein A, Schady NR. Conditional cash transfers: reducing present and future poverty: World Bank Publications; 2009.
8. Rawlings LB, Rubio GM. Evaluating the impact of conditional cash transfer programs. The World Bank Research Observer. 2005;20(1):29-55.
9. Fernald LC, Gertler PJ, Neufeld LM. Role of cash in conditional cash transfer programmes for child health, growth, and development: an analysis of Mexico's Oportunidades. The Lancet. 2008;371(9615):828-37.
10. Levere M, Acharya G, Bharadwaj P. The role of information and cash transfers on early childhood development: evidence from Nepal. National Bureau of Economic Research, 2016.
11. Robertson L, Mushati P, Eaton JW, Dumba L, Mavise G, Makoni J, et al. Effects of unconditional and conditional cash transfers on child health and development in Zimbabwe: a cluster-randomised trial. The Lancet. 2013;381(9874):1283-92.
12. Pega F, Liu SY, Walter S, Pabayo R, Saith R, Lhachimi SK. Unconditional cash transfers for reducing poverty and vulnerabilities: effect on use of health services and health outcomes in low-and middle-income countries. The Cochrane Library. 2017.
13. Hamadani JD, Huda SN, Khatun F, Grantham-McGregor SM. Psychosocial stimulation improves the development of undernourished children in rural Bangladesh. The Journal of nutrition. 2006;136(10):2645-52.
14. Ahmed SM, Evans TG, Standing H, Mahmud S. Harnessing pluralism for better health in Bangladesh. The Lancet. 2013;382(9906):1746-55.

15. Ministry of Women and Children Affairs GoB. the Children Policy 2011. 2011.
16. Britto PR, Lye SJ, Proulx K, Yousafzai AK, Matthews SG, Vaivada T, et al. Nurturing care: promoting early childhood development. The Lancet. 2017;389(10064):91-102.
17. Hamadani JD, Huda SN, Khatun F, Grantham-McGregor SM. Psychosocial stimulation improves the development of undernourished children in rural Bangladesh. J Nutr. 2006;136(10):2645-52.
18. Nahar B, Hamadani J, Ahmed T, Tofail F, Rahman A, Huda S, et al. Effects of psychosocial stimulation on growth and development of severely malnourished children in a nutrition unit in Bangladesh. European Journal of Clinical Nutrition. 2009;63(6):725-31.
19. Nahar B, Hossain MI, Hamadani JD, Ahmed T, Huda SN, Grantham-McGregor SM, et al. Effects of a community-based approach of food and psychosocial stimulation on growth and development of severely malnourished children in Bangladesh: a randomised trial. Eur J Clin Nutr. 2012;66(6):701-9.
20. Tofail F, Hamadani JD, Mehrin F, Ridout DA, Huda SN, Grantham-McGregor SM. Psychosocial stimulation benefits development in nonanemic children but not in anemic, iron-deficient children. J Nutr. 2013;143(6):885-93.

Budget [Please add]

Project name: Urban Lactating Allowance and PS
PI: Sheikh Jamal Hossain
Duration: 24 months
2018 to 2020
Conversion rate icddr,b rate for Feb 2018 = 1 USD = 1.27 CAD

Items name	Level	Number	Rate	Effort	Months	Total in USD-Y1	Total in CAD-Y1	months	Rate	effort	Total in USD-Y2	Total in CAD-Y2	Total USD Y1+Y2	Total CAD Y1+Y2
Personnel														
PI (Jamal)	NOB/2	1	2,600	40%	11	11,442	14,531	11	2,860	52%	16,432	20,869	27,874	35,400
Co-PI-Dr Jena	P4/3	1	15,634	5%	11	8,083	10,265	12	16,416	5%	9,317	11,833	17,400	22,098
Co-I -Dr Fahmida	NOD/5	1	5,403	5%	11	2,675	3,397	6	5,944	5%	1,605	2,038	4,279	5,435
Co_I_Dr. Muhith	NOB/2	1	2,600	20%	3	1,560	1,982					-	1,560	1,982
Consultant		1				-	-				3,000	3,810	3,000	3,810
Finance Manager	NOA/2	1	2,079	25%	9	4,725	6,000	9	2,287	25%	5,199	6,603	9,924	12,603
HR Manager	NOA/7	1	2,277	25%	9	5,123	6,507	9	2,505	20%	4,554	5,783	9,677	12,290
Field Research Officer (coordination)	GS5-CSA	1	653	100%	10	6,534	8,298	6	719	100%	4,312	5,477	10,846	13,775
Field Research Supervisor (field & quality assuarence)	GS4/1-	1	506	100%	9	4,554	5,784	5	557	100%	2,783	3,534	7,337	9,318
Research Fellow /Field research Supervisor	GS4/1-	1	506	100%	9	4,554	5,784	9	557	100%	5,009	6,362	9,563	12,146
Field research Assistant (tester) for tests	GS3/1-CSA	6	428	100%	4	10,270	13,042	4	471	100%	11,297	14,347	21,566	27,389
Intervention workers (FO)	Sp level	11	138	100%	7	10,588	13,446	7	151	100%	11,646	14,791	22,234	28,237
Attendent		1	575	60%	6	2,070	2,629	12	633	55%	4,175	5,302	6,245	7,931
Data collectors (To identify mothers)		5	113	100%	5	2,833	3,597		-		-	-	2,833	3,597
Subtotal						75,009	95,261				79,329	100,748	154,338	196,009
Travel														
Travel costs of testers/mothers for baseline		660	3			1,980	2,515					-	1,980	2,515
Travel costs of testers/mothers for endline		660				-	-		3		1,980	2,515	1,980	2,515
Travel and related cost (Govt. Personnel, PI, CoPI, project personnel & others stake holders)						3,300	4,191				2,500	3,175	5,800	7,366
PI Int Travel(ticket, perdium & others)		1	3,000	1	1	3,000	3,810		3,000	1	4,000	5,080	7,000	8,890
Subtotal						8,280	10,516				8,480	10,770	16,760	21,285
Reimbursable goods and services													-	
Bayley record form & others		1,320	5.5	1	1	7,260	9,220				-	-	7,260	9,220
Bayley Permission Fee and related cost		500	3	1	1	1,500	1,905				-	-	1,500	1,905
Stimulation materials & related cost		1	330	1	2	660	838	1			-	-	660	838
Supplies item and Others						800	1,016				1,199	1,522	1,999	2,538
Meeting & workshop, dissemination and (all related cost)				1	1	1,500	1,905				1,000	1,270	2,500	3,175
Subtotal						11,720	14,884				2,199	2,792	13,919	17,676
Reimburseable Project Administration cost														
Printing and photocopy		1	500	1	1	540	686	1	1,000	1	1,000	1,270	1,540	1,956
Participants wageloss (660 participants and 90 training)		750	2	1	1	1,125	1,429	1	2	1	1,050	1,334	2,175	2,762
legal fees(RRC & ERC)						1,530	1,943					-	1,530	1,943
Field Office Rent						800	1,016				500	635	1,300	1,651
Communication (mobile, Phone, courier & etc)		1	1,089	1	1	1,089	1,383	1	1,000	1	1,000	1,270	2,089	2,653
Subtotal						5,084	6,457				3,550	4,509	8,634	10,965
Equipments														
Computer and accessories for data entry		1	2,500	1	1	2,500	3,175				-	-	2,500	3,175
Scanner			700	1	1	700	889				-	-	700	889
Subtotal						3,200	4,064				-	-	3,200	4,064
Grand Total_USD						103,293	131,182				93,558	118,818	196,850	250,000

22.02.18
Md. Rezaur Rahman
Manager, Finance
MCHD, icddr,b
25 FEB 2018

Budget Justifications

Please provide one page statement justifying the budgeted amount for each major item, including the use of human resources, major equipment, and laboratory services.

Remuneration for Project lead, Co project lead, coinvestigator, filed research officer, supervisor and testers etc will be incurred as required for the project. Travel cost was calculated as per project requirement. Reimbursable goods and services have been calculated for the project implementation. Reimbursable project administration cost include printing and photocopy, participants wage loss, expenditure for RRC and ERC, Field office rent and communication.

Other Support

Describe sources, amount, duration, and grant number of all other research funding currently granted to PI or under consideration.

N/A

Biography of the Investigators

Provide biographical data in the following format for all key personnel including the Principal Investigator. Copy the same format for each of them.
Note: Biography of the External Investigators may, however, be submitted in the format as convenient to them..

Biography of the Principal Investigator

Provide biographical data in the following format for all key personnel including the Principal Investigator. Copy the same format for each of them.
Note: Biography of the External Investigators may, however, be submitted in the format as convenient to them..

1. **Name: Sheikh Jamal Hossain**
2. **Present Position: Senior** Research Investigator
3. **Educational background**: (last degree and diploma & training relevant to the present research proposal)

	Institution	Year
Master of Public Health (HP&HE)	NIPSOM, University of Dhaka	2009
Master in Health Economics	Institute of Health Economics, University of Dhaka	2002
Post Graduate Diploma in Health Economics	Institute of Health Economics, University of Dhaka	2001
Scientific Project Management	icddr,b	2014
Training on Result Based Management	icddr,b	2015

4. **Ethics Certification:**

		If Yes		
		Issuing Authority	Registration No	Valid Until
**No** ☐	Yes ☒	NIH	2117415	

Note: If the response is "no", please get certification from CITI or NIH before study initiation and submit a copy to the Committee Coordination Secretariat

5. **List of ongoing research protocols/ activities**

Protocol/ Activity Number	Role (PI, Co-PI, Co-I)	Starting date	End date	Percentage of time
16063	Co-I	1/6/16	30/5/20	50%
1709	PI	1/7/2017	31/3/2019	50%%

6. **Publications**

Types of publications	Numbers
a) Original scientific papers in peer-review journals	2
b) Peer reviewed articles and book chapters	
c) Papers in conference proceedings	7
d) Letters, editorials, annotations, and abstracts in peer-reviewed journals	
e) Working papers	
f) Monographs	1

7. **Five recent publications including publications relevant to the present research protocol**

1. *Hasan, M. I**., **Hossain, S. J***., Braat, S., Dibley, M. J., Fisher, J., Grantham-McGregor, S., ... & Biggs, B. A. (2017). Benefits and risks of Iron interventions in children (BRISC): protocol for a three-arm parallel-group randomised controlled field trial in Bangladesh. *BMJ open*, *7*(11), e018325.
2. **Hossain SJ**, Tofail F, Hasan MI, Fardina M, Hamadani J. Gender Differences in the Quality of Psychosocial Stimulation in Rural Bangladesh Homes. Journal of Child: Care Health and Development; 2018. In Press
3. F Akter, RA Sarker, , SJ Hossain, F Tofail 'Adaptation of Cognitive Behavioral Therapy (CBT) in the cultural perspective of Bangladesh; an integration of quantitative and qualitative approach' (Year:2014) abstract presentation at 'National Public health conference'
4. RA Sarker, F Akter, SJ Hossain, F Tofail. 'Mothers' perception about depressive symptoms: A qualitative study '(Year: 2014) abstract presentation at 'National Public health conference'.

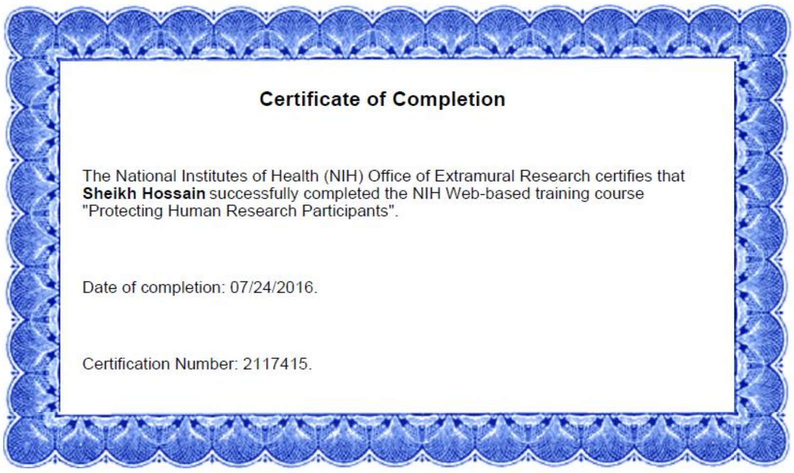
Certificate of Completion

The National Institutes of Health (NIH) Office of Extramural Research certifies that **Sheikh Hossain** successfully completed the NIH Web-based training course "Protecting Human Research Participants".

Date of completion: 07/24/2016.

Certification Number: 2117415.

Biography of the co principal Investigators

Provide biographical data in the following format for all key personnel including the Principal Investigator. Copy the same format for each of them.
Note: Biography of the External Investigators may, however, be submitted in the format as convenient to them..

1. **Name: Dr. Jena D. Hamadani**

2. **Present Position:** Scientist

3. **Educational background**: (last degree and diploma & training relevant to the present research proposal)

	Institution	Year
Ph D in Child Development	Institute of Child Health, University College London	2004
Diploma in Child Health	Bangladesh Institute of Child Health, Dhaka University	1996
MBBS	Rajshahi Medical College	1984
Training on WPPSI-III and MABC	Institute of Child Health, University College London, U.K.	2005
Training on methods of psychosocial stimulation	Tropical Metabolism Research Unit, University of West Indies, Jamaica	2000

4. **Ethics Certification:**

		If Yes		
		Issuing Authority	Registration No	Valid Until
**No** ☐	Yes ☒	NIH	945553	
**No** ☐	Yes ☒	ICH Good Clinical Practice	193335	

Note: If the response is "no", please get certification from CITI or NIH before study initiation and submit a copy to the Committee Coordination Secretariat

5. List of ongoing research protocols/ activities

Protocol/ Activity Number	Role in the protocol/ activity (PI, Co-PI, Co-I)	Starting date	End date	Percentage of time
PR-17064	PI	01/06/2018	31/05/2019	25
PR-17096	PI	29/09/2017	31/03/2019	30
PR-17009	Co-PI	01/07/2017	30/06/2019	9
PR-16063	PI	1/12/2016	28/02/2020	20

6. Publications

Types of publications	Numbers
a. Original scientific papers in peer-review journals	73
b. Peer reviewed articles and book chapters	1
c. Papers in conference proceedings	3
d. Letters, editorials, annotations, and abstracts in peer-reviewed journals	63
e. Working papers	2
f. Monographs	5

7. Five recent publications including publications relevant to the present research protocol

- **Hamadani JD**, Tofail F, Huda SN, Alam DS, Ridout DA, Attanasio O, Grantham-McGregor SM. Cognitive deficit and poverty in the first 5 years of childhood in Bangladesh. Pediatrics. 2014 Oct;134(4):e1001-8. doi:10.1542/peds.2014-0694.
- Tofail F, **Hamadani JD**, Mehrin F, Ridout DA, Huda SN, Grantham-McGregor SM. Psychosocial stimulation benefits development in nonanemic children but not in anemic, iron-deficient children. *Journal of Nutrition* 143:885-893, 2013.
- **Hamadani JD,** Tofail F, Hilaly A, Mehrin F, Shiraji S, Banu S, S.N. Huda SN. Association of postpartum maternal morbidities with children's mental, psychomotor and language development in rural Bangladesh. J Health Popul Nutr 2012 Mar;30(2):193-204.
- **Hamadani JD,** Tofail F, Nermell B, Gardner R, Shiraji S, Bottai M, Arifeen SE, Huda SN, Vahter M. Critical windows of exposure for arsenic-associated impairment of cognitive function in pre-school girls and boys: a population-based cohort study. Int J Epidemiol. 2011 Dec;40(6):1593-604.
- **Hamadani JD,** Nahar B, Huda SN, Tofail F. Integrating Early Child Development programs into health and nutrition services in Bangladesh: Benefits and challenges. Annals of the New York Academy of Sciences 2014 Jan;1308:192-203. doi: 10.1111/nyas.12319, 2013.

Certificate of Completion

The National Institutes of Health (NIH) Office of Extramural Research certifies that **Jena Hamadani** successfully completed the NIH Web-based training course “Protecting Human Research Participants”.

Certificate of Completion

Date of completion: 07/01/2012

Certification Number: 945553

Biography of the Co-Investigators

Provide biographical data in the following format for all key personnel including the Principal Investigator. Copy the same format for each of them.
Note: Biography of the External Investigators may, however, be submitted in the format as convenient to them.

1. **Name: Dr. Fahmida Tofail**
2. **Present Position:** Scientist & Sr. Consultant Physician (Registration Number: A 23209)
3. **Educational background**: (last degree and diploma & training relevant to the present research proposal)

	Institution	Year
Post. Doc	Division of Metals & Health, Karolinska Institutet, Stockholm, Sweden,	Continuing
PhD in Child Development	Institute of Child Health, University College London	2006
MBBS	Mymensing Medical College	1992

4. **Ethics Certification:**

		If Yes		
		Issuing Authority	Registration No	Valid Until
**No** ☐	Yes ☒	National Institutes of Health (NIH)	945557	

Note: If the response is “no”, please get certification from CITI or NIH before study initiation and submit a copy to the Committee Coordination Secretariat

5. **List of ongoing research protocols/ activities**

Protocol/ Activity Number	Role in the protocol/ activity (PI, Co-PI, Co-I)	Starting date	End date	Percentage of time
PR-16037 (RINEW)	Co-I	2016	2018	20
PR-14014	Co-PI	2014	2017	10
PR-14110 (Neuro)	Co-PI	2014	2017	10

6. **Publications**

Types of publications	Numbers
a. Original scientific papers in peer-review journals	56
b. Peer reviewed articles and book chapters	3
c. Papers in conference proceedings	
d. Letters, editorials, annotations, and abstracts in peer-reviewed journals	26
e. Working papers	
f. Monographs	1

7. **Five recent publications including publications relevant to the present research protocol**

1. **Tofail F,** Fernald LC, Das KK, Rahman M, Ahmed T, Jannat KK, Unicomb L, Arnold BF, Ashraf S, Winch PJ, Kariger P, Stewart CP, Colford JM Jr, Luby SP. Effect of water quality, sanitation, hand washing, and nutritional interventions on child development in rural Bangladesh (WASH Benefits Bangladesh): a cluster-randomised controlled trial. Lancet Child Adolesc Health. 2018 Apr;2(4):255-268.
2. Matias SL, Mridha MK, **Tofail F,** Arnold CD, Khan MS, Siddiqui Z, Ullah MB,Dewey KG. Home fortification during the first 1000 d improves child development in Bangladesh: a cluster-randomized effectiveness trial. Am J Clin Nutr. 2017 Apr;105(4):958-969.
3. **Tofail F,** Hamadani JD, Mehrin F, Ridout D, Huda SN, Grantham-McGregor SM. Psychosocial stimulation benefits development in nonanemic children but not in anemic, iron-deficient children. J Nutr. 2013 Jun;143(6):885-93.
4. **Tofail F**, Hamadani JD, Mehrin F, Huda SN. Play materials at home and growth in early infancy show positive association with development of poor urban Bangladeshi infants. Early Child hood Development- A key to child rights. Islam Z and Mamun AA. Early Concern and Manusher Jonno Foundation. 2011; 172-81
5. **Tofail F.** & Hamadani JD. Prevalence of iron-defciency anaemia among young children in rural Bangladesh Health and Science Bulletin (English) Vol. 8 No. 2 June 2010; pg-1-22 *(IF:KN)*

Biography of the Co-Investigators

1. **Name: Mohiuddin Ahsanul Kabir Chowdhury**

2. **Present Position: Senior Research Investigator**
   Centre for Child and adolescent Health
   International Centre for Diarrhoeal Diseases Research, Bangladesh

3. **Educational background**: (last degree and diploma& training relevant to the present research proposal)

	Institution	Year
MPH	James P. Grant School of Public Health	2014
MBBS	Chittagong Medical College	2004

4. **Ethics Certification:**

		If Yes		
		Issuing Authority	Registration No	Valid Until
**No** ☐	Yes ☒	**NIH**	**1439132**	

Note: If the response is “no”, please get certification from CITI or NIH before study initiation and submit a copy to the Committee Coordination Secretariat

5. **List of ongoing research protocols/ activities**

Protocol/ Activity Number	Role in the protocol/ activity (PI, Co-PI, Co-I)	Starting date	End date	Percentage of time
PR-16013	Co-I	April 2016	June 2019	20%
PR-16024	Co-PI	July 2016	September 2017	20%
PR-16030	PI	July 2016	September 2017	40%
PR-15074	Co-I	June 2015	December 2016	20%
PR-17033	PI	June 2017	April, 2018	50%
PR-17177	PI	November, 2017	February, 2018	40%

6. Publications

Types of publications	Numbers
a. Original scientific papers in peer-review journals	2
b. Peer reviewed articles and book chapters	
c. Papers in conference proceedings	1
d. Letters, editorials, annotations, and abstracts in peer-reviewed journals	
e. Working papers	
f. Monographs	

7. Five recent publications including publications relevant to the present research protocol

7.1. **Chowdhury, Mohiuddin Ahsanul Kabir;** Salma Morium. "Domestic violence against women: a historic and socio-cultural reality in Bangladesh." *European Scientific Journal, ESJ* 11.26 (2015).

7.2. **Chowdhury, M.A.K**., Anwar, R. and Saha, A., 2018. Ambiguous genitalia–A social dilemma in Bangladesh: A case report. International journal of surgery case reports, 42, pp.98-101.

Biography of the Co-Investigators

Provide biographical data in the following format for all key personnel including the Principal Investigator. Copy the same format for each of them.
Note: Biography of the External Investigators may, however, be submitted in the format as convenient to them..

1. **Name:** Dr. Mohammed Imrul Hasan
2. **Present Position:** Senior Research Investigator
3. **Educational background**: (last degree and diploma & training relevant to the present research proposal)

	Institution	Year
Masters in Public Health	State University of Bangladesh	2013
M.B.B.S	Dhaka Medical College, Bangladesh	2001
IMCI Clinical Management Training		2005

4. **Ethics Certification:**

		If Yes		
		Issuing Authority	Registration No	Valid Until
**No** ☐	Yes ☒	11/26/2013	1334483	

Note: If the response is "no", please get certification from CITI or NIH before study initiation and submit a copy to the Committee Coordination Secretariat

5. List of ongoing research protocols/ activities

Protocol/ Activity Number	Role (PI, Co-PI, Co-I)	Starting date	End date	Percentage of time
PR-16063	Co-I	2016	2020	100
PR-17096	Co-I	2017	2019	00

6. Publications

Types of publications	Numbers
**a.** Original scientific papers in peer-review journals	07
**b.** Peer reviewed articles and book chapters	
**c.** Papers in conference proceedings	
**d.** Letters, editorials, annotations, and abstracts in peer-reviewed journals	01
**e.** Working papers	
**f.** Monographs	

7. Five recent publications including publications relevant to the present research protocol

a. ***Hasan, M. I***., Hossain, S. J., Braat, S., Dibley, M. J., Fisher, J., Grantham-McGregor, S., ... & Biggs, B. A. (2017). Benefits and risks of Iron interventions in children (BRISC): protocol for a three-arm parallel-group randomised controlled field trial in Bangladesh. *BMJ open*, *7*(11), e018325.

b. Takeuchi, H., Khan, A. F., Yunus, M., ***Hasan, M. I***., Hawlader, M. D. H., Takanashi, S., ... & Nakahara, S. (2016). Anti-Ascaris immunoglobulin E associated with bronchial hyper-reactivity in 9-year-old rural Bangladeshi children. *Allergology International*, *65*(2), 141-146.

c. Ali, H., Hamadani, J., Mehra, S., Tofail, F., ***Hasan, M. I***., Shaikh, S., ... & Christian, P. (2017). Effect of maternal antenatal and newborn supplementation with vitamin A on cognitive development of school-aged children in rural Bangladesh: a follow-up of a placebo-controlled, randomized trial. *The American Journal of Clinical Nutrition*, ajcn134478.

d. Sternäng, O., Palmer, K., Kabir, Z. N., **Hasan, M. I**., & Wahlin, Å. (2018). Associations Between Functional Biological Age and Cognition Among Older Adults in Rural Bangladesh: Comparisons With Chronological Age. *Journal of aging and health*, 0898264318757147.

e. Qureshi, N. K., Hossain, T., ***Hassan, M. I***., Akter, N., Rahman, M. M., Sultana, M. M., ... & Latif, Z. A. (2017). Neck circumference as a marker of overweight and obesity and cutoff values for Bangladeshi adults. *Indian Journal of Endocrinology and Metabolism*, *21*(6), 803.

f. Islam, S., F. Nusrat, S. A. Esha, F. Mehrin, A. Hilaly, S. Shiraji, **M. I. Hasan**, F. Tofail, and J. D. Hamadani. "How Does Family Structure Relate With Children's Language Development? A Cross Sectional Experience from Urban Slums in Dhaka." *Mymensingh medical journal: MMJ* 26, no. 4 (2017): 775-782.

g. Takeuchi, H., Khan, A. F., **Hasan, M. I**., Hawlader, M. D. H., Yunus, M., Zaman, K., ... & Iwata, T. (2016). Comment on IgE responses to Ascaris and mite tropomyosins are risk factors for asthma. Clinical & Experimental Allergy, 46(1), 178-180.

h. Hossain SJ, Tofail F, **Hasan MI**, Fardina M, Hamadani J. Gender Differences in the Quality of Psychosocial Stimulation in Rural Bangladesh Homes. Journal of Child: Care Health and Development; 2018. In Press

Certificate of Completion

The National Institutes of Health (NIH) Office of Extramural Research certifies that **Mohammed Imrul Hasan** successfully completed the NIH Web-based training course “Protecting Human Research Participants”.

Date of completion: 11/26/2013

Certification Number: 1334483

Biography of the Co-Investigators

1. **Name:** Syeda Fardina Mehrin
2. **Present Position:** Research Investigator
3. **Educational background**: (last degree and diploma & training relevant to the present research proposal)

	Institution	Year
Degree	M.Sc in Psychology , University of Dhaka	2000
Degree	BSC in Psychology , University of Dhaka	1999
Training	Advance Biostatistical method and STATA	2016
Training	Advance course on Epidemiology, Research method and SPSS	2016
Training	Scientific Project Management, icddr,b	2014
Training	Exclusive course on “Pathway to Manuscript Writing”, Training and Education Unit (TEU), icddr,b	2009
Training	Bayley-III in India, WPPSI, MABC in UK	
Training	Introductory Course on Epidemiology and Biostatistics at icddr,b	2003

4. **Ethics Certification:**

		If Yes		
		Issuing Authority	Registration No	Valid Until
**No** ☐	Yes ☒	NIH	1335239	

5. **List of ongoing research protocols/ activities**

Protocol Number	Role in the protocol	Starting date	End date	% of time
PR-17096	Co-I	25/10/2017	31/03/2019	70

6. **Publications**

Types of publications	Numbers
g. Original scientific papers in peer-review journals	5
h. Peer reviewed articles and book chapters	2
i. Papers in conference proceedings	
j. Letters, editorials, annotations, and abstracts in peer-reviewed journals	
k. Working papers	

l. Monographs	

7. Five recent publications including publications relevant to the present research protocol

- Hossain SJ, Tofail F, Hasan MI, Fardina M, Hamadani J. Gender Differences in the Quality of Psychosocial Stimulation in Rural Bangladesh Homes. Journal of Child: Care Health and Development; 2018. In Press
- Gustin, K., Tofail, F., **Mehrin, F**., Levi, M., Vahter, M., & Kippler, M. (2017). Methylmercury exposure and cognitive abilities and behavior at 10 years of age. *Environ Int*. doi: 10.1016/j.envint.2017.02.004
- Tofail F, Hamadani JD, **Mehrin F**, Ridout D, Huda SN, Beard J, Grantham-McGregor SM. Randomized controlled trial of stimulation on development of iron deficient anemic and non-anemic children. Submitted to Am J Clin Nutr 2011.
- Hamadani JD, Tofail F, Hilaly A, **Mehrin** F, Shiraji S, Huda SN. Association of maternal morbidities with children's development. J Health Popul Nutr 2011.
- Tofail F, Hamadani JD, Ahmed AZ, **Mehrin F**, Hakim M, Huda SN. The mental development and behavior of low-birth-weight Bangladeshi infants from an urban low-income community. Eur J Clin Nutr. 2011 Sep 28. doi:10.1038/ejcn.2011.165.
- Hamadani JD, Baker-Henningham H, Tofail F, **Mehrin F**, Huda SN, Grantham-McGregor SM. The validity and reliability of mothers' report of language development in one year old children in a large scale survey in Bangladesh. Food Nutr Bull 2010 Jun:31(2 Suppl):S198-206.

Biography of the Co-Investigators

Name: Sk. Masum Billah

1. **Present Position: Senior** Research Investigator
   Maternal and Chils Health Division
   International Centre for Diarrhoeal Disease Research, Bangladesh

2. **Educational background**: (last degree and diploma& training relevant to the present research proposal)

	Institution	Year
**Degree**		
MPH	The University of Melbourne, Australia	2012
BURP	Khulna University, Khulna, Bangladesh	2005
**Trainings**		
Impact Evaluation of Population, Health and Nutrition Programs	Measure Evaluation	2013
Short course on International Child health	The University of Melbourne, Australia	2011
Introductory course on Epidemiology and Biostatistics	icddr,b	2008
Introductory course of Bioethics	icddr,b	2007

3. **Ethics Certification:**

		If Yes		
		Issuing Authority	Registration No	Valid Until
**No** ☐	Yes ☒	NIH	1419101	Date of completion: 03/03/2014

Note: If the response is "no", please get certification from CITI or NIH before study initiation and submit a copy to the Committee Coordination Secretariat

4. **List of ongoing research protocols/ activities**

Protocol/ Activity Number	Role in the protocol/ activity (PI, Co-PI, Co-I)	Starting date	End date	Percentage of time

PR-17084	PI	2017	30/4/2018	80%
PR-15122	PI	2014	30/4/2018	20%
PR-15107	PI	2015	30/4/2018	50%
PR-14124	PI			

5. Publications

Types of publications	Numbers
m. Original scientific papers in peer-review journals	3
n. Peer reviewed articles and book chapters	
o. Papers in conference proceedings	
p. Letters, editorials, annotations, and abstracts in peer-reviewed journals	
q. Working papers	
r. Monographs	

6. Five recent publications including publications relevant to the present research protocol

a. Hoque DME, Arifeen SE, Rahman M, Chowdhury EK, Haque TM, Begum K, Hossain MA, Akter T, Haque F, Anwar T, **Billah SM**, Rahman AE, Huque MH, Christou A, Baqui AH, Bryce J and Black RE; **Improving and sustaining quality of child health care through IMCI training and supervision: experience from rural Bangladesh**. *Health Policy and* Planning. 2013 doi:10.1093/heapol/czt05

b. Billah, S. M., Saha, K. K., **Khan, A. N. S**., Chowdhury, A. H., Garnett, S. P., El Arifeen, S., & Menon, P. (2017). Quality of nutrition services in primary health care facilities: Implications for integrating nutrition into the health system in Bangladesh. PloS one, 12(5), e0178121.

c. Billah SM, Ferdous TE, Karim MA, Dibley MJ, Raihana S, Moinuddin M, et al. A community-based cluster randomised controlled trial to evaluate the effectiveness of different bundles of nutrition-specific interventions in improving mean length-for-age z score among children at 24 months of age in rural Bangladesh: study protocol. BMC public health. 2017;17(1):375

d. Sk Masum Billah, D M Emdadul Hoque, Muntasirur Rahman, Aliki Christou, Ngatho Samuel Mugo, Khadija Begum, Tazeen Tahsina, Qazi Sadeq-ur Rahman, Enayet K Chowdhury, Twaha Mansurun Haque, Rasheda Khan, Ashraf Siddik, Jennifer Bryce, Robert E Black, Shams El Arifeen, Feasibility of engaging "Village Doctors" in the Community-based Integrated Management of Childhood Illness (C-IMCI): Experience from rural Bangladesh; Journal of Global Health (accepted, due for publication)

e. Hoque DM, Rahman M, **Billah SM**, Savic, Karim AR, Chowdhury EK, Hossian A, Musa SM, Kumar H, Malhotra S, Matin S, Raina N, Weber M, El Arifeen S; **An assessment of the quality of care for children in eighteen randomly selected district and sub-district hospitals in Bangladesh.** BMC Pediatir.2012;12:197

f. Shams E Arifeen, DM Emdadul Hoque, Tasnima Akter, Muntasirur Rahman, Mohammad Enamul Hoque, Khadija Begum, Enayet K Chowdhury, Rasheda Khan, Lauren S Blum, Shakil Ahmed, M Altaf Hossain, Ashraf Siddik, Nazma Begum, Qazi Sadeq-ur Rahman, Twaha M Haque, **Sk. Masum Billah**, Mainul Islam, Reza Ali Rumi ,Erin Law, ZA Motin Al-Helal, Abdullah H Baqui, Joanna Schellenberg, Taghreed Adam, Lawrence H. Moulton, Jean-Pierre Habicht, Robert Scherpbier, Cesar Victora, Jennifer Bryce, Robert E Black; **Effect of Integrated Management of Childhood Illness strategy on childhood mortality and nutrition in a rural area in Bangladesh: a cluster randomized trial**. The Lancet. 2009;374:393–403

Biography of the Co Investigators

Provide biographical data in the following format for all key personnel including the Principal Investigator. Copy the same format for each of them.**Note**: Biography of the External Investigators may, however, be submitted in the format as convenient to them..

1. **Name:** Shamima Shiraji
2. **Present Position:** Research Investigator
3. **Educational background**: (last degree and diploma & training relevant to the present research proposal)

	Institution	Year
Degree	M.Sc in Psychology , University of Dhaka	2002
Degree	BSC in Psychology , University of Dhaka	2000
Training	Advance course on Epidemiology, Research method and SPSS	2016
Training	Basic Course on **Qualitative Research**	2015
Training	Training course on **Information Literacy and Literature Search**	2012
Training	Introductory Course on Epidemiology and Biostatistics at icddr,b	2007
Training	**HIV counselling STI (Sexual Transmitted diseases)** conducted by Family Health International (FHI)	2006

4. **Ethics Certification:**

		If Yes		
		Issuing Authority	Registration No	Valid Until
**No** ☐	Yes ☒	NIH	**2369629**	

Note: If the response is "no", please get certification from CITI or NIH before study initiation and submit a copy to the Committee Coordination Secretariat

5. **List of ongoing research protocols/ activities**

Protocol/ Activity Number	Role in the protocol/ activity (PI, Co-PI, Co-I)	Starting date	End date	Percentage of time

6. **Publications**

Types of publications	Numbers
a. Original scientific papers in peer-review journals	3
b. Peer reviewed articles and book chapters	
c. Papers in conference proceedings	
d. Letters, editorials, annotations, and abstracts in peer-reviewed journals	
e. Working papers	
f. Monographs	

7. Five recent publications including publications relevant to the present research protocol

1. Hamadani JD**,** Tofail F, Hilaly A, Mehrin F, **Shiraji** S, Banu S, S.N. Huda SN. Association of postpartum maternal morbidities with children's mental, psychomotor and language development in rural Bangladesh. J Health Popul Nutr 2012 Mar;30(2):193-204
2. Hamadani JD, Tofail F, Hilaly A, Mehrin F, **Shiraji S**, Huda SN. Association of maternal morbidities with children's development., J Health Popul Nutr 2011.
3. Hamadani JD, Tofail F, Nermell B, Gardner R, **Shiraji S**, Bottai M, Arifeen SE, Huda SN, Vahter M. Critical windows of exposure for arsenic-associated impairment of cognitive function in pre-school girls and boys: a population-based cohort study. Int J Epidemiol. 2011 Dec;40(6):1593-604.

Certificate of Completion

The National Institutes of Health (NIH) Office of Extramural Research certifies that **Shamima Shiraji** successfully completed the NIH Web-based training course "Protecting Human Research Participants".

Date of completion: 04/04/2017.

Certification Number: 2369629.

Biography of the Co-Investigator

Provide biographical data in the following format for all key personnel including the Principal Investigator. Copy the same format for each of them.
Note: Biography of the External Investigators may, however, be submitted in the format as convenient to them..

8. **Name: Jane Fisher**
9. **Present Position:** Professor of Global Health, Head of Division of Social Sciences, Monash University
10. **Educational background**: (last degree and diploma & training relevant to the present research proposal)

	Institution	Year
Doctor of Philosophy	University of Melbourne	1994
Registered Specialist Clinical Psychologist	Psychologists Registration Board of Victoria	1994
Registered Psychologist	Psychologists Registration Board of Victoria	1977
Bachelor of Science Honours (Psychology)	University of Queensland	1973

11. **Ethics Certification:**

		If Yes		
		Issuing Authority	Registration No	Valid Until
**No** ☐	Yes ☒	Monash University	N/A	12/10/2019

Note: If the response is "no", please get certification from CITI or NIH before study initiation and submit a copy to the Committee Coordination Secretariat

12. **List of ongoing research protocols/ activities**

Protocol/ Activity Number	Role (PI, Co-PI, Co-I)	Starting date	End date	Percentage of time
APP1164736. Refugee women, intimate partner violence and settlement: The first cohort study.	Co-PI	2019	2022	5%
1146034 .Addressing an unrecognised public health problem in Vietnam: a clustered randomised controlled trial of the culturally adapted Resourceful Adolescent Program (RAP-V) to improve adolescent mental health	Co-PI	2019	2021	10%

TTS-1803-22331. Learning clubs to improve women's health & infant's health and development in Vietnam. A cluster randomised controlled trial to inform transition to scale.	PI	2018	2019	10%
APP1100147. Learning clubs to improve women's health and infant's health and development in Vietnam: a cluster randomised controlled trial of a low-cost, evidence-informed, structured intervention	PI	2016	2019	20%
APP1163202. Eggsurance? A randomisedơ controlled trial of a novel Decision Aid for women considering egg freezing	Co-PI	2019	2021	5%
APP1153592. Centre of Research Excellence on Women and Non-communicable Disease (CRE WaND): Prevention and Detection	Co-PI	2019	2023	5%
APP1153419. The Centre of Research Excellence in Sexual and Reproductive Health for Women: Achieving Better Outcomes through Primary Care	Co-PI	2019	2023	5%
APP1153419. Centre of Research Excellence in Childhood Adversity and Associated Depression and Anxiety	Co-PI	2019	2023	5%
Preparing for Parenthood	Co-PI	2019	2021	5%
Latrobe Valley early parenting - What Were We Thinking	PI	2018	2020	5%
Review and Evaluation of the Jean Hailes for Women's Health Anxiety Portal and e-resources for women and anxiety	PI	2018	2019	2%
Contributing to optimal mental health for older women in Australia: a multiple methods research program	PI	2018	2019	5%
Provision of a perinatal depression online support tool and smartphone application	PI	2017	2019	5%
APP1103262. Defining the impact of universal iron interventions in young children:	Co-PI	2016	2020	5%

a randomized controlled trial in rural Bangladesh				

13. Publications

Types of publications	Numbers
g) Original scientific papers in peer-review journals	176
h) Peer reviewed articles and book chapters	63
i) Papers in conference proceedings	4
j) Letters, editorials, annotations, and abstracts in peer-reviewed journals	3
k) Working papers	5
l) Monographs	

14. **Five recent publications including publications relevant to the present research protocol**

- FISHER J, Cabral de Mello M, Patel V, Rahman A, Tran T, Holton S, Holmes W. Prevalence and determinants of common perinatal mental disorders in women in low and lower middle income countries: a systematic review of the evidence. Bulletin of the World Health Organization 2012; 90:139–149.

- Rahman A, FISHER J, Bower P, Luchters S, Tran TD, Yasamy T, Saxena S, Waheed W. Interventions for common perinatal mental disorders in women in low- and middle-income countries: a systematic review and meta-analysis Bulletin of the World Health Organization, 2013; 91:593–601

- Tran TD, Tran T, Simpson JA, Tran HT, Nguyen TT, Hanieh S, Dwyer T, Biggs BA, FISHER J. Infant motor development in rural Vietnam and intrauterine exposures to anaemia, iron deficiency and common mental disorders: A prospective community-based study. BMC Pregnancy and Childbirth 2014, 14:8, DOI:10.1186/1471-2393-14-8

- Tran TD, Biggs BA, Tran T, Simpson JA, Cabral de Mello M, Hanieh S, Nguyen TT, Dwyer T, FISHER J. Perinatal common mental disorders among women and the social and emotional development of their infants in rural Vietnam. Journal of Affective Disorders 2014; 160:104–112. DOI: 10.1016/j.jad.2013.12.034

- Tran TD, Biggs BA, Tran T, Simpson JA, Hanieh S, Dwyer T, FISHER J. Impact on infants' cognitive development of antenatal exposure to iron deficiency disorder and common mental disorders. PLoS ONE 2013;8(9):e74876. DOI: 10.1371/journal.pone.0074876

Biography of the Principal Investigator

Provide biographical data in the following format for all key personnel including the Principal Investigator. Copy the same format for each of them.
Note: Biography of the External Investigators may, however, be submitted in the format as convenient to them..

15. **Name: Thach Tran**

16. **Present Position: Research Fellow, Monash University**

17. **Educational background**: (last degree and diploma & training relevant to the present research proposal)

	Institution	Year
Doctor of Philosophy	The University of Melbourne, Australia	2014
Master of Science in Health Development (Epidemiology)	Chulalongkorn University, Thailand	2007
Bachelor of Economics (Hons)	Phuong Dong University, Vietnam	1998

18. **Ethics Certification:**

		If Yes		
		Issuing Authority	Registration No	Valid Until
**No** ☐	Yes ☒	Monash University	N/A	25/08/2021

Note: If the response is "no", please get certification from CITI or NIH before study initiation and submit a copy to the Committee Coordination Secretariat

19. **List of ongoing research protocols/ activities**

Protocol/ Activity Number	Role (PI, Co-PI, Co-I)	Starting date	End date	Percentage of time
Addressing an unrecognised public health problem in Vietnam: a clustered randomised controlled trial of the culturally adapted Resourceful Adolescent Program (RAP-V) to improve adolescent mental health	PI	2019	2021	20%
Learning clubs to improve women's health & infant's health and development in Vietnam. A cluster randomised controlled trial to inform transition to scale.	Co-PI	2018	2019	5%

Improving child health and development in resource-constrained settings: A multi-component study to inform policy and more effective interventions	PI	2016	2019	30%
Learning clubs to improve women's health and infant's health and development in Vietnam: a cluster randomised controlled trial of a low-cost, evidence-informed, structured intervention	Co-PI	2016	2019	5%

20. Publications

Types of publications	Numbers
m) Original scientific papers in peer-review journals	65
n) Peer reviewed articles and book chapters	5
o) Papers in conference proceedings	12
p) Letters, editorials, annotations, and abstracts in peer-reviewed journals	
q) Working papers	7
r) Monographs	

21. Five recent publications including publications relevant to the present research protocol

Tran TD, Luchters S, Fisher J. Early childhood development: impact of national human development, family poverty, parenting practices and access to early childhood education. *Child: Care, Health and Development* 2017**;** 43 (3), 415–426 DOI: 10.1111/cch.12395.

Tran TD, Biggs B-A, Hoton S, Nguyen H, Hanieh S, Fisher J. Comorbid anaemia and stunting among preschool-aged children in low- and middle-income countries: A syndemic. *Public Health Nutrition* 2019; 22(1):35-43. DOI: 10.1017/S136898001800232X

Tran TD, Tran T, Simpson JA, Tran HT, Nguyen TT, Hanieh S, Dwyer T, Biggs BA, Fisher J. Infant motor development in rural Vietnam and intrauterine exposures to anaemia, iron deficiency and common mental disorders: A prospective community-based study. *BMC Pregnancy and Childbirth* 2014, 14:8. DOI:10.1186/1471-2393-14-8

Tran TD, Biggs BA, Tran T, Simpson JA, Cabral de Mello M, Hanieh S, Nguyen TT, Dwyer T, Fisher J. Perinatal common mental disorders among women and the social and emotional development of their infants in rural Vietnam. *Journal of Affective Disorders* 2014; 160:104–112. DOI: 10.1016/j.jad.2013.12.034

Tran TD, Biggs BA, Tran T, Simpson JA, Hanieh S, Dwyer T, Fisher J. Impact on infants' cognitive development of antenatal exposure to iron deficiency disorder and common mental disorders. *PLoS ONE* 2013;8(9):e74876. DOI: 10.1371/journal.pone.0074876

icddr,b

Grand Challenges Canada
Grands Défis Canada
BOLD IDEAS WITH BIG IMPACT

অংশগ্রহনের সম্মতি পত্র

প্রটোকল নং-১৮০৩৫	ভার্শন নং– ৩	তারিখঃ ২৬/০৬/২০১৮

ইভালুয়েটিং ইফেক্ট অফ ইনটিগ্রেটিং ই সি ডি একটিভিটিস ইনটু বাংলাদেশ গভর্ণমেন্টস্ আরবান ল্যাকটেটিং মাদারস্ এলাওয়েন্স প্রোগ্রাম ফর দি পুওর অন চিলড্রেনস্ কগনিশান এন্ড বিহেবিয়র ।

প্রধান গবেষক ঃ শেখ জামাল হোসেন

প্রতিষ্ঠান ঃ আর্ন্তজাতিক উদরাময় গবেষণা কেন্দ্র, বাংলাদেশ (আই,সি,ডি,ডি,আর,বি)

গবেষণার উদ্দেশ্য ঃ

আমরা আই সি ডি ডি আর, বি থেকে এটি গবেষনা পরিচালনা করছি। বাংলাদেশের শহর এলাকার দুগ্ধদানকারী মায়েদেরকে অর্থ ভাতা প্রদানের সাথে শিশুদের বুদ্ধিবৃত্তিক ও আচরনের উপর মনোঃসামাজিক উদ্দীপনার প্রভাব সম্পর্কে অবহিত হওয়ার লক্ষ্যে।

গবেষনার বিষয়টির সংক্ষিপ্ত বিবরন এবং এই গবেষনার প্রয়োজনীয়তা ঃ

উন্নয়নশীল দেশ সমূহে আনুমানিক ২শত মিলিয়ন শিশুরা দারিদ্রতা ও ঝুঁকি সমুহের কারনে তাদের পূর্ণ বিকাশে পৌঁছেনা। ৫ বছর বয়সের নীচের বাংলাদেশের অর্ধেকের বেশী শিশুদের দারিদ্রতা ও গৃহে পর্যাপ্ত উদ্দীপনা প্রদানের অভাবে বিকাশের ঝুঁকিতে থাকে।কিছু সময়ে স্বাস্থ্যসেবায় মাত্রাতিরিক্ত খরচের কারনে দরিদ্ররা আরো দরিদ্র হয় এবং দারিদ্রতার চক্রের মধ্যে পড়ে থাকে। দরিদ্র মায়েদেরকে শর্তমুক্ত ও শর্তহীন নগদ অর্থ প্রদান বিশ্বব্যাপী দারিদ্রতা দূরীকরনে ও মায়েদের এবং শিশুদের স্বাস্থ্য উন্নতির ক্ষেত্রে একটি উপায় হিসাবে প্রমাণিত হয়েছে। মহিলা ও শিশু বিষয়ক মন্ত্রনালয় দুগ্ধদানকারী শহর এলাকার মায়েদেরকে ভাতা প্রদান করছেন। এখন পর্যন্ত আমরা জানিনা যে স্বাস্থ্য বিষয়ক এই প্রকল্পের সাথে মনোঃসামাজিক উদ্দীপনার সংযোগ ঘটানোর ধারনাটি কি শিশুদের বিকাশের জন্য ভাল হবে কিনা। এই প্রকল্পে আমরা দেখতে চাচ্ছি বাংলাদেশের শহর এলাকার দুগ্ধদানকারী মায়েদেরকে অর্থ ভাতা প্রদানের সাথে শিশুদের বুদ্ধিবৃত্তিক ও আচরনের উপর মনোঃসামাজিক উদ্দীপনার প্রভাব সম্পর্কে ।

কেন আপনাকে এই গবেষনায় অংশগ্রহনের জন্য আহ্বান করা হচ্ছে ঃ

আপনি সরকার কর্তৃক শর্তহীন নগদ অর্থ পাচ্ছেন এ ছাড়াও আপনার ৬-১৬ মাস বয়সী একটি শিশু রয়েছে।

কর্ম পদ্ধতি ও প্রণালী/কার্যপ্রণালী (এই গবেষনায় অংশগ্রহন কারীর কাছ থেকে কি আশা করা হবে।) ঃ

এই গবেষনায় ৬০০ শিশুকে অন্তর্ভুক্ত করা হবে। ৩০০ জন অংশগ্রহন কারী শর্তহীন নগদ অর্থ এবং মনোঃসামাজিক উদ্দীপনা পাবে এবং ৩০০ জন শুধু শর্তহীন নগদ অর্থ মোট ২টি দলে অন্তর্ভুক্ত করা হবে। আপনি যে কোন একটি দলে অন্তর্ভুক্ত হবেন দেবচয়ন প্র দ্ধি তে.

যদি আপনি এই গবেষনায় অংশগ্রহন করতে সম্মত হন, সে ক্ষেত্রে একজন প্রশিক্ষিত পরীক্ষক আপনার আর্থ- সামাজিক অবস্থা, জীবন যাত্রার গুনগত মান , শিশু প্রতিপালন বিষয়ে মায়ের জ্ঞান, আপনার ডিপ্রেসন বিষয়ক উপসর্গ, পরিবারের খাদ্য নিরাপত্তার অবস্থা ও মাসিক পারিবারিক খরচ,স্বাস্থ্য খরচ, স্বাস্থ্য সেবা নেওয়ার অভ্যাস,ঘরে আপনার শিশু যে ধরনের খেলনা নিয়ে খেলে ও উদ্দীপনার কার্যক্রম পেয়ে থাকে, , এ ছাড়া সে আপনার শিশুর সাথে খেলার মাধ্যমে মেধা পরীক্ষা করবে। এছাড়াও আপনার এবং আপনার শিশুর উচ্চতা ,ওজন, এবং শিশুর উর্ধবাহুর মাঝের পরিধি এবং মাথার পরিধির পরিমাপনেওয়া হবে । এই পরিমাপটি এই গবেষনার শুরুতে ও শেষে করা হবে। এই সাক্ষাৎকারের জন্য ৪০-৫০ মিনিট সময় লাগবে। আমরা আপনাকে এবং আপনার শিশুকে এই গবেষনার প্রথমে এবং বছর শেষে আপনার বাড়ির আশেপাশের কোন স্থানীয় অফিসের কাছে নিয়ে খেলা-ধূলার মাধ্যমে তার মেধার বিকাশ সম্পর্কে জানবো। এটি করতে প্রায় ১ঘন্টা সময় লাগবে। তাছাড়া দৈবচয়ন পদ্ধতিতে কিছু মাকে নির্দিষ্ট করে তাদের বাড়িতে নির্দিষ্ট কর্মী ১৫ দিন পর পর গিয়ে বাচ্চার সাথে কিভাবে খেলতে হয় সে বিষয়ে আলোচনার মাধ্যমে দেখিয়ে দিবে। এটি করতে প্রায় ১ঘন্টা সময় লাগবে।

ঝুঁকি এবং সুবিধাদি ঃ

এতে আপনার শিশুর জন্য কোন ঝুঁকি নেই বরং এটি একটি মজার খেলার মত। যাই হোক আপনার শিশুর যদি কোন সুস্পষ্ট প্রতিবন্ধকতা থাকে, যা নিয়ে আপনি উদ্বিগ্ন, তার জন্য আমরা তাকে সঠিক প্রতিষ্ঠানে পাঠাবো এবং একটি রেফারেল কার্ড দিব যাতে আপনি সহজে আপনার শিশুকে তাদের কাছে নিয়ে যেতে পারেন।

গোপনীয়তা, নামহীনতা ও বিশ্বস্ততাঃ

আপনার থেকে নেয়া গবেষণা সম্পর্কিত সকল তথ্যের গোপনীয়তা রক্ষা করা হবে।কাগজে যে সকল তথ্য নেয়া হবে তা তালাবদ্ধ অবস্থায় সংগ্রহ করা হবে এবং কম্পিউটারে রাখা তথ্য সমূহ গোপনসংকেত এর মাধ্যমে সংরক্ষন করা হবে। আমাদের আই সি ডি ডি আর বির ইথিকাল রিভিউ কমিটি এবং গবেষণার সাথে সংশিষ্ট কর্মী বাদে এ তথ্য আর কেউ ব্যাবহার করতে পারবে না।

তথ্যের ভবিষ্যৎ ব্যবহারঃ

এই গবেষণায় সংগৃহীত তথ্য ভবিষ্যতের ব্যাবহারের জন্য রাখা হবে কোন কোন গবেষণালব্ধ তথ্যাদি হয়তোবা অন্যকোন গবেষকদের সাথে উপস্থাপন করা যেতে পারে, তবে সেক্ষেত্রে অবশ্যই আপনার নাম পরিচয়ের গোপনীয়তা রক্ষা করা হবে। পরবর্তীতে গবেষণার জন্য আমরা আপনার কাছে আবারও আসতে পারি।

গবেষণায় অংশগ্রহণ ও প্রত্যাহারের অধিকার ঃ

আপনার শিশুর গবেষণায় অংশগ্রহণ হচ্ছে ঐচ্ছিক। আপনি গবেষণায় অংশগ্রহণ নাও করতে পারেন অথবা যেকোন সময় নাম প্রত্যাহার করতে পারেন। তাতে আপনাকে কোন মাসুল দিতে হবেনা বা আই,সি,ডি,ডি,আর,বি হাসপাতালে চিকিৎসায় কোন ক্ষতি হবেনা।

কে এই গবেষণা সম্বন্ধে আমার প্রশ্নের উত্তর দিতে পারবে:

আপনার যদি কোন প্রশ্ন থাকে আপনি তা আমাদেরকে জিজ্ঞাসা করতে পারেন। এ ছাড়াও পরবর্তী সময়ে গবেষণা সংশিষ্ট কোন প্রশ্ন থাকলে আপনি এই ফোন নম্বরে ৮৮৬০৫২৩, এক্সটেনশন-২৩৩৩, সরাসরি শেখ জামাল হোসেন (০১৭১২১৯১৪১৪) (কলেরা হাসপাতাল, মহাখালী) কে জিজ্ঞাসা করতে পারেন। অধিকন্তু আপনি জনাব এম. এ. সালাম খান, কোয়ার্ডিনেসন ম্যানেজার, রিসার্চ এ্যাডমিনেস্ট্রেসন, ফোন-৯৮৮৬৪৯৮ অথবা ৮৮৬০৫২৩-৩২ এক্সটেনশন-৩২০৬ এ যোগাযোগ করতে পারেন। আপনি যদি এই গবেষণায় আপনি/আপনার শিশুর অংশগ্রহণে রাজি থাকেন তাহলে, তা নীচে স্বাক্ষর দিয়ে অথবা বাম হাতের বৃদ্ধাঙ্গুলির ছাপ দিন। আপনার সহযোগিতার জন্য অনেক ধন্যবাদ।

অংশগ্রহণ কারী নিজের এবং তার শিশুর পক্ষে ______________________ তারিখ: ______________________
স্বাক্ষর/বাম বৃদ্ধাঙ্গুলির ছাপ বৃদ্ধাঙ্গুলির ছাপ

তার শিশুর স্বাক্ষর/বাম বৃদ্ধাঙ্গুলির ছাপ বৃদ্ধাঙ্গুলির ছাপ ______________________ তারিখ: ______________________

সাক্ষীর স্বাক্ষর/বাম বৃদ্ধাঙ্গুলির ছাপ ______________________ তারিখ: ______________________

গবেষকের/প্রতিনিধির সাক্ষর ______________________ তারিখ: ______________________

Consent Form

Protocol No.18035	Version No. 1.0	Date: 25/04/2018

Protocol Title: **Evaluating effects of integrating ECD activities into Bangladesh Government's urban lactating mothers allowance program for the poor on children's cognition and behavior**

Investigator's name: Sheikh Jamal Hossain

Organization: icddr,b

Purpose of the research

We from icddr,b are conducting a research to know if play and stimulation along with receiving lactating allowance in urban settings improves Children's Cognition and Behaviour.

Background (brief introduction of the issue and the need for/ importance of the research)
In developing countries, more than 200 million of children do not reach their maximum potentiality because of poverty and associated risk factors. More than half of the Bangladeshi children <5 years are at risk for developmental delay due to poverty and sub-optimal home stimulation. Sometimes poor people become poorer due to catastrophic expenditure on health care and fall into the vicious cycle of poverty. Conditional and unconditional cash transfer to the poor mothers has proved a way of reducing poverty and improving health outcomes for mothers and children worldwide. Ministry of Women and Children's Affairs is providing lactating allowance for the poor urban mothers. We do not yet know if adding play and stimulation to this program improves child cognition and behaviour. With this study we intend to know if play and stimulation along with receiving lactating allowance in urban settings improves Children's Cognition and Behaviour

Why invited to participate in the study?
You were identified eligible to receive lactating allowance from government and your child is aged 6-16 months.

Methods and procedures [What is expected from the participants of the research study?]

In this study we include 600 children in two arms, per arm contain 300 children. The arms are (1) Lactating allowance + Psychosocial stimulation and 2 Only lactating allowance

If you agree to participate in the study, we will visit you and your child and collect some information about your quality of life, food availability in your family, your income and expenditure, how you seek health during any illness, any violence experienced by you in your family, type of toys and stimulation activities your child receives at home, etc. This visit will take about 40-50 minutes of your time. We will also bring you and your child at the beginning of the study and after a year to a nearby local office and see how your child plays with some toys to assess his/her behaviour and brain development. At that session, we will measure your child's weight, length, arm and head circumference, your weight, height and arm circumference, your depressive symptoms and collect information on your socio-economic condition. This session takes about one hour. In addition, we will select some of the participants, based on lottery to be visited every fortnight by an intervention

worker who will discuss with you how to play and stimulate your child. The fortnightly visits will take about one hour.

Risk and benefits

There is no risk to your child and it will be more like a fun game. However, if there is any obvious disability in your child that concerns you, we will refer him/her to the proper institutions and will refer you so that you can easily take your child to them.

Privacy, anonymity and confidentiality

All information collected from you will remain strictly confidential. Paper based record will be kept under lock, and computerised data will be password-protected. None other than the research staff of our study and the Ethical Review Committee of icddr,b that protects the interest of research participants will have access to your information.

Future use of information

Some of the information collected from this study may be kept for future use however in such cases information and data supplied to other researchers, will not conflict with or violate the maintenance of privacy, anonymity and confidentiality of information identifying participants in any way. We may come again to you for further research.

Right not to participate and withdraw

Your child's participation in the study is voluntary, and you are the sole authority to decide for and against your child's participation in this study. Refusal to take part in the study will involve no penalty. Even if you do not agree to join the study, or if you want to withdraw your participation from the study, you will still receive the same quality of medical care available to you through icddr,b, the government, and other organisation.

Answering your questions/ Contact persons

You are free to ask us questions about the study, if you have any. If you have additional questions later, you may contact me; Sheikh Jamal Hossain at icddr,b (Phone # 8860523-32, Ext-2333) at anytime required. Moreover, you can contact Mr. M.A. Salam Khan, Coordination Manager, Research Administration at 9886498 or 8860523-32. Ext.3206.

If you agree to our proposal of enrolling you and your child in our study, please indicate that by putting your signature or your left thumb impression at the specified space below. Thank you for your cooperation

______________________________ ________________
Signature or left thumb impression of participant Date

______________________________ ________________
Signature or left thumb impression of Parent/ Guardian/ Attendant Date

______________________________ ________________
Signature or left thumb impression of the witness Date

______________________________ ________________
Signature of the PI or his/her representative Date

(NOTE: In case of representative of the PI, she/he shall put her/his full name and designation and then sign)

International Centre for Diarrhoeal Disease Research, Bangladesh (icddr,b)
Maternal and Child Health Division (MCHD)
Project title: Unconditional Lactational Allowance + Pyshosocial Stimulation
Baseline Questionnaire

Child ID: /___/___/___/___/___/___/

Socio-Demographic and Economic Information (SES PART-A)

Child's ID (বাচ্চার আইডি) :

Child's Name (বাচ্চার নাম) :

Mother's Name (মায়ের নাম) :

Address with detail location (পূর্ণ ঠিকানা) :
Mobile Number:
Date of Test মূল্যায়ন গ্রহনের তারিখ (দিন/মাস/বছর): | _ | _ | / | _ | _ | / | _ | _ | _ | _ |

QUESTIONS<br>প্রশ্নসমূহ:	CODING CATEGORIES<br>কোডিং ক্যাটাগরিসমূহ
1. পরিবারের সদস্য সংখ্যা (কয়জন সদস্য একই পাতিলে খায়?) (Number of Family Members(How many members eat with same cooking)	|___|___|
2. আপনার ধর্ম কি? ১= ইসলাম, ২= হিন্দু, ৩= বৌদ্ধ, ৪= খ্রীষ্টান, ৭৭= অন্যান্য উল্লেখ করুন<br>(What is your religion? 1= Islam 2= Hindu 3= Buddist 4= Christian 77=Others Mention)__________	|___|___|
3. বাড়ীর মধ্যে শিশুর সংখ্যা (শূণ্য থেকে পাঁচ বছর বয়সের) (Number of children in the household(zero to five years)	|___|___|
4. মায়ের গর্ভধারণের সংখ্যা (যতবার অন্তসত্ত্বা হয়েছেন) (Number of pregnancy(How many times she was pregnant)	|___|___|
4.1 আপনার সন্তানের (বাচ্চার নাম)জন্ম কোথায় উল্লেখ করুন (Where was the birth place of the indexed (Research Participant) child.	1. Home<br>2. Hospital/clinics/ health centre
4.2 আপনার সন্তানকে কত মাস শুধু বুকের দুধ খাইয়েছেন ?(How many months do you feed breast exclusively?)	
5. মায়ের ছেলে সন্তানের সংখ্যা (Number of son she has)	|___|___|
6. মায়ের মেয়ে সন্তানের সংখ্যা (Number of daughter she has)	|___|___|

6.1 মায়ের বয়স (Age of mother) (year)	|___|___|
7. পিতার মূল পেশা কি? (যদি একটির বেশি পেশা হয়, তাহলে যেটা প্রধান তা উল্লেখ করুন) (Principle profession of the father(if more than one, please mention main profession)<br>0= বেকার (Unemployed)<br>1= অনিয়মিত চাকুরী (দিন মজুর, অদক্ষ শ্রমিক, গৃহস্থালী শ্রমিক, রিকশা/ভ্যান চালক, জেলে, অন্যের জমিতে কাজ করেন, সরবরাহকারী প্রভৃতি) (Irregular job: daily labour, unskill labour, household labour, richshaw/van puller, fisherman, agriculture labour who work other's field, supplier etc)<br>2= নিয়মিত চাকুরী (নিজস্ব ব্যবসা, দোকানদার, কারুশিল্পী, প্রাইভেট চাকুরী, বেতনভূক্ত , কারখানা এবং অফিসের দক্ষ কর্মচারী, বিক্রয়কর্মী) (Regular job: self business, shopkeeper, artist, private job, salaried employed, skill labour of industry and skill staff of a office, sales man)<br>77= অন্যান্য (উল্লেখ করুন) Others (Mention)______________________	|___|___|
8. মায়ের মূল পেশা কি? (যদি একটির বেশি পেশা হয়, তাহলে যেটা প্রধান তা উল্লেখ করুন) (Principle profession of the mother(if more than one, please mention main profession)<br>0= গৃহিনী (housewife)<br>1= অনিয়মিত চাকুরী (গৃহস্থালী, অন্যের খামারে কাজ করেন প্রভৃতি)(Irregular job: household chore, work other's agriculture field etc)<br>2= নিয়মিত চাকুরী (নিজস্ব জমিতে কৃষিকাজ করেন, নিজস্ব ব্যবসা, দোকানদার, কারুশিল্পী, প্রাইভেট চাকুরী বেতনভূক্ত, কারখানা এবং অফিসের দক্ষ কর্মচারী, মহিলা বিক্রয়কর্মী, সরকারী চাকুরীজীবী) (Regular job: self agricultural employed, self business, shopkeeper, artist, private job, salaried employed, skill labor of industry and skill staff of a office, sales man, government worker)<br>77= অন্যান্য (উল্লেখ করুন) Other (Mention) __________________________	|___|___|
9. মায়ের শিক্ষাগত যোগ্যতা (যে কয় বছর বিদ্যালয়ে পড়েছেন) (Mother's educational qualification(years of education)<br>12+ ডিগ্রি/ পাসকোর্স (পুরানো) = ২ বছর (12+ degree/pass course(old) = 2 years<br>12+ ডিগ্রি/ পাসকোর্স (পুরানো) = ৩ বছর (12+ degree/pass course(new) = 3 years<br>12+ অনার্স = ৪ বছর (12+ honours = 4 years)	|___|___|
10. পিতার শিক্ষাগত যোগ্যতা (যে কয় বছর বিদ্যালয়ে পড়েছেন) Father's educational qualification(years of education)<br>12+ ডিগ্রি/ পাসকোর্স (পুরানো) = ২ বছর (12+ degree/pass course(old) = 2 years)<br>12+ ডিগ্রি/ পাসকোর্স (নতুন) =৩ বছর (12+ degree/pass course(new)=3 years)<br>12+ অনার্স = ৪ বছর (12+ honours = 4 years)	|___|___|
11. প্রতি মাসে পরিবারে কত টাকা আয় হয় (Monthly income of the family in Taka)	

11.1 প্রতি মাসে পরিবারে কত টাকা ব্যয় হয় (Monthly expenditure of the family in Taka)	
12. আয়-ব্যায়ের ঘাটতি (মনে করুন) (Income and expenditure deficit (recall)<br>1 = সবসময় (always), 2 = মাঝে মধ্যে (sometimes), 3 =কখনোই না (never)	|__|__|
13. ঘরের ছাদ কি দিয়ে তৈরী (roof material)<br>1 = খড় (straw), 2 = টিন (tin), 3 = সিমেন্ট (cement), 4 = কাঠ (wood), 77 = যদি অন্য কিছু হয় উল্লেখ করুন (if anything else please mention)__________________	|__|
14. ঘরের মেঝে কি দিয়ে তৈরী (floor material)<br>1 = মাটি (Mud), 2 = খড়/ বাঁশ(straw/bamboo), 3 = সিমেন্ট (cement), 4 = কাঠ (wood),<br>77 = যদি অন্য কিছু হয় উল্লেখ করুন (if anything else please mention) ______________	|__|
15. ঘরের দেয়াল কি দিয়ে তৈরী (wall material)<br>1 = মাটি (Mud), 2 = খড়/ বাঁশ (straw/bamboo), 3 = সিমেন্ট (cement), 4 = টিন (tin),<br>77 = যদি অন্য কিছু হয় উল্লেখ করুন (if anything else please mention) ____________	|__|
16. আপনার ঘরে কতগুলো রুম আছে?(শুধু মাত্র যে ঘরগুলো থাকার জন্য ব্যবহার করা হয় , রান্না ঘর অন্তর্ভুক্ত হবে কিন্তু বাথরুম, গোসলখানা অন্তর্ভুক্ত হবে না) (How many rooms are there for sleep: kithen will be included but bathroom, toilet will be excluded)	|__|
17. আপনার ঘরে কি বিদ্যুৎ সংযোগ আছে? Do you have electricity in your house<br>1 = না (No) , 2 = হ্যাঁ (yes)	|__|
18. পানীয় জলের উৎস কি? What are the source of drinking water<br>1 = পুকুরের পানি (Pond water), 2 = সাপ্লাই পানি (Supply water), 3 = টিউবওয়েলের পানি (Tube-wel water), 77 = যদি অন্য কিছু হয় উল্লেখ করুন (if anything else please mention)	|__|
19. সাধারনত কোথায় রান্না করা হয়?(Where are the place of cooking)<br>1= যে ঘরে থাকা হয় বা ঘুমানো হয় (Sleeping room)<br>2= একই ঘরে আলাদা রুম রান্নাঘর হিসেবে ব্যবহার হয় (Same roof but different room)<br>3= আলাদা ঘর রান্নাঘর হিসেবে ব্যবহার হয় (different room)<br>4= ঘরের বাহিরে (Outside of the room)<br>77= অন্যান্য (উল্লেখ করুন) (if anything else please mention)<br>____________________	| __ |
20. আপনার ঘরে কি ধরণের টয়লেট সুবিধা আছে? (What are the toilet facility in your household)<br>1=কোন নির্দিষ্ট জায়গা নেই (কোন সুবিধা নেই, ঝোপঝাড়, মাঠ) (No specific place (no facility, bushy place , open field)<br>2=কাঁচা খোলা পায়খানা (ঝুলন্ত, খোলা,খোলা গর্ত) Katcha open toilet(hang, open, open hole)<br>3=সেমি-সেনিটারী (পানি সিল করার ব্যবস্থা আছে) (Semi sanitary(water seal)<br>4=সেনিটারী (সেপটিক ট্যাঙ্ক, আধুনিক টয়লেট) (Sanitary ,septic tank, modern toilet)	| __ |

21.আবাসন/ বাড়ি- (Residence)<br>1= অন্যের জায়গায় ফ্রি থাকে (own others but free),<br>2= ভাড়া (to-let) ,<br>3 =নিজের বাড়ী (own residence),<br>77= অন্যান্য উল্লেখ করূন) (if anything else please mention) -----------	| __ |
22. আপনার ঘরে (বা ঘরের যেকোন সদস্যের) কতগুলো জিনিস আছে?) (Do you belong to these materials)	
22.1. আলমারি (Almira)	| __ |
22.2. ড্রেসিং টেবিল (DressingTable)	| __ |
22.3. খাবার টেবিল (Dining Table)	| __ |
22.4. টেলিভিশন (Television)	| __ |
22.5. ফ্রিজ (Fridge)	| __ |
22.6. মোবাইল ফোন (Mobile Telephone)	| __ |
22.7 কার/ মাইক্রোবাস / মোটর সাইকেল (Car/Microbus/Motor cycle)	| __ |
22.8 কম্পিউটার / ল্যাপটপ (Computer/Laptop)	| __ |
23. নিচের প্রাণীগুলোর মধ্যে আপনার কয়টি গৃহপালিত পশু-পাখি আছে?	
23.1 গরু / মহিষ. (Cow/Buffalo)	|___|___|
23.2. ছাগল / ভেড়া (Goats / Lamb)	|___|___|
23.3. মুরগী /হাঁস (Chickens / Ducks)	|___|___|

Child's and Mom's Anthropometric Measurements

Anthropometry	
Weight of child in<br>শিশুর ওজন (গ্রাম)	|____|____||____||____|____| (gram)
Height of child in<br>শিশুর উচ্চতা (সেমি.)	|____|____||____|.|____| (cm)
MUAC of child in<br>শিশুর (সেমি.)	|____|____|.|____|(cm)
Head Circumference<br>শিশুর (সেমি.)	|____|____|.|____|(cm)
Weight of Mother<br>মায়ের ওজন (গ্রাম)	|____|____||____||____|____| (gram)
Height of Mother<br>মায়ের উচ্চতা (সেমি.)	|____|____||____|.|____|(cm)
MUAC of Mother	|____|____|.|____|(cm)

icddr,b KNOWLEDGE FOR GLOBAL LIFESAVING SOLUTIONS

Grand Challenges Canada Grands Défis Canada BOLD IDEAS WITH BIG IMPACT®

International Centre for Diarrhoeal Disease Research, Bangladesh (icddr,b)
Maternal and Child Health Division (MCHD)
Project title: **Unconditional Lactational Allowance + Pyshosocial Stimulation**

Name of child: ______________________ Child Id:
Name of Mother: ______________________ Date of interview: / / /

শিশুর পরিচর্যা বিষয়ে প্রশ্নাবলী
(Parenting)

তথ্য প্রদানকারীকে বলুন: এখানে কোন সঠিক বা ভুল উত্তর বলে কিছু নেই। আমরা শুধু আপনার মতামত জানতে চাই । আমি আপনাকে একটি প্রশ্ন করব, অত:পর প্রশ্নটির কয়েকটি সম্ভাব্য উত্তর বলব। আপনি আপনার জন্য প্রযোজ্য উত্তরটি আমাকে বলবেন।”

P.01	বাচ্চার (নাম) মধ্যে ভালো কিছু দেখলে যেমন; সুন্দর কাপড় বা জামা পরলে অথবা বাচ্চা কোন ভাল কাজ করলে আপনি কি তখন তাকে প্রশংসা করেন?	কখনও প্রশংসা করি না = ০,<br>খুব কম প্রশংসা করি= ১,<br>মাঝে মাঝে করি= ২,<br>সব সময় প্রশংসা করি = ৩	|___|
P02	বাচ্চারা কথা না শুনলে বা দুষ্টামি করলে কেউ কেউ মারধর করেন, আপনি কি মনে করেন?	বশ্যই মার দেওয়ার দরকার আছে=০ মাঝে মাঝে মারতে হয়=১,<br>একটু আধটু মারতে হয়=২,<br>একদম মার দেওয়া উচিৎ না=৩	|___|
03	আপনি যতই ব্যস্ত থাকেন না কেন, বাড়ীর কাজের সময় যেমন - ঘর পরিস্কার বা রান্না করার সময় বাচ্চার (নাম) সাথে কতটা কথা বলেন ?	এত ব্যস্ত যে কথা বলার সময় পাইনা =০<br>খুব কম কথা বলি =১<br>মাঝে মাঝে কথা বলি =২<br>বেশীর ভাগ সময় কথা বলি =৩	|___|
04	আপনি যতই ব্যস্ত থাকেন না কেন কাজের ফাঁকে ফাঁকে যেমন- ঘর পরিস্কার বা রান্না করার সময় বাচ্চার (নাম) সাথে কতটা খেলা করেন?	এত ব্যস্ত যে খেলা করার সময় পাইনা =০<br>ওর সাথে খুব কম খেলি =১<br>মাঝে মাঝে খেলা করি =২<br>বেশীর ভাগ সময় ওর সাথে খেলা করি =৩	|___|
05	তিন বছরের নীচের বাচ্চাদের লেখাপড়া শেখানো যেমন অ, আ, ক, খ বা যোগ বিয়োগ শেখানো কতটা দরকার বলে আপনি মনে করেন?	**জানিনা/অবশ্যই শেখানো দরকার=০,**<br>**মাঝে মাঝে শেখানো দরকার=১,**<br>**একটু আধটু শেখাতে পারি=২,**<br>**তিন বছরের আগে শেখানো উচিৎ না=৩**	|___|
06	লবণে আয়োডিন আছে কিনা তা ঘরে বসে পরীক্ষা করা সম্ভব। আপনি কি পরীক্ষা করে দেখেছেন ?	কখনো পরীক্ষা করিনি =০,<br>খুব কম করি=১<br>মাঝে মধ্যে করি =২<br>সব সময় করি =৩	|___|
07	মা যদি অসুস্থ হয়, যেমন- জ্বর,কাশি ঠান্ডা ডায়রিয়ায় কোন কোন মা বাচ্চাকে বুকের দুধ বন্ধ করে দেয়, আপনি কি করেন/ করেছেন?	জানিনা/বুকের দুধ বন্ধ করে দেই/ দিয়েছি =০, বেশীর ভাগ সময়ের জন্য বন্ধ করে দেই/ দিয়েছি =১,<br>মাঝে মাঝে বন্ধ করে দেই/ দিয়েছি =২<br>অসুস্থ থাকলেও বন্ধ করা উচিৎ না =৩	|___|
08	ওজন এবং উচ্চতার সাথে বাচ্চাদের বুদ্ধি বিকাশের কোন সম্পর্ক আছে বলে কি আপনি মনে করেন?	জানিনা/ না=০,<br>শুধু উচ্চতার সাথে সম্পর্ক আছে =১<br>শুধু ওজনের সাথে সম্পর্ক আছে=১,<br>উচ্চতা এবং ওজনের উভয়ের সাথে সম্পর্ক আছে =৩	|___|
9	বাচ্চাদের বুদ্ধি বিকাশের জন্য খাবারের মধ্যে আয়রণ বা আয়রণ সমৃদ্ধ খাবারের কি দরকার আছে?	জানিনা/কোন দরকার নেই =০<br>একটু আধটু দরকার =১<br>কিছুটা দরকার =২<br>যথেষ্ট আয়রন দরকার =৩	
10	কোন বয়স থেকে বাড়তি খাবার বাচ্চাকে দিতে হবে?		|___| মাস

(নীচের উত্তরগুলো পড়ে শুনাবেন না , মায়ের উত্তরগুলো লিখুন এবং নীচের উত্তরের সাথে মিলিয়ে স্কোর করুন)			
11	কোন কাজ শেখাতে গেলে বাচ্চা কিছুটা ঠিক ভাবে করে কিছুটা ভুল ভাবে করে। কোন কাজ পুরোটা ঠিক ভাবে শেখানোর জন্য আপনি কি করেন?	যেটুকু ঠিক ভাবে করতে পেরেছে তার জন্য বার বার প্রশংসা করি=১<br>যেটুকু ভুল করেছে তা বার বার ঠিক করে করতে বলি =০	|___|
12	খাওয়ানোর সময় বাচ্চা যদি ঠিকমত খেতে না চায় তবে কি কি কায়দা করে খাওয়ান ?<br><br>ক) অল্প অল্প করে খাওয়াই/ বারে বারে ধৈর্য্য ধরে খাওয়াই/ বিভিন্ন রকমের খাবার দেই<br>খ) আদর যত্ন করি/ কোলে নিয়ে বসাই গ) গল্প করি/ / গান গাই/ ছড়া বলি<br>গ) এটা ওটা দেখাই/ অভিনয় করি/ খেলা করি<br><br>অন্যান্য (উলেখ কর্ন) __________	জোর করে খাওয়াই, মার দেই, বকা দেয়া, ভয় দেখাই, লোভ দেখাই + অন্য যে কোন উত্তর =০<br>একটি উত্তর বা টিকের জন্য=১ ,<br>দুটি উত্তর বা টিকের জন্য =২ ,<br>দুয়ের অধিক =৩	|___|
13	বাচ্চা যাতে সময়মত কথা বলতে এবং বুঝতে পারে সে জন্য আপনি কি কি করেন ?<br><br>ক) বিভিন্ন জিনিষ (যেমন: ছবির নাম / রং এর নাম/ মানুষের নাম/পশুপাখীর নাম ইত্যাদি) এর নাম শিখাই<br>খ) কথা বলতে বলতে শিখাই / বারে বারে কথা বলি<br>গ) গল্প/ছড়া/কবিতা/গান করি ঘ) অভিনয় করি/ খেলা করি<br><br>অন্যান্য (উলেখ কর্ন)________________________	কিছু করি না, কথাতো এমনি এমনি শিখবে =০,<br>একটি উত্তর বা টিকের জন্য=১,<br>দুটি উত্তর বা টিকের জন্য=২,<br>দুয়ের অধিক=৩	|___|
14	আপনার বাচ্চাকে (নাম) ছবির বই দেখিয়ে দেখিয়ে কি শেখান যায় এবং কি ভাবে শেখান যায় ?<br>ক) জিনিসের নাম যেমন;ছবির নাম / রং এর নাম/ মানুষের নাম/পশুপাখীর নাম) শেখাই<br>খ) কবিতা/ গান/ গল্প করে শেখাই গ)অভিনয়/ খেলা করে শেখাই<br><br>অন্যান্য (উলেখ কর্ন)________________________	শিখাই না বা শেখানো যায় না =০<br>একটি উত্তর বা টিকের জন্য =১,<br>দুটি উত্তর বা টিকের জন্য=২,<br>দুয়ের অধিক=৩	|___|
15	আপনার বাচ্চার (নাম) সাথে পুতুল দিয়ে কি কি করেন বা শেখান ?<br><br>ক) বিভিন্ন জিনিষের নাম (যেমন;শরীরের অংশ, কাপড়ের নাম ইত্যাদি) শেখাই<br>খ) আদর করা/প্রশংসা করা শেখাই<br>গ)গল্প/ছড়া/ গান/ কবিতা বলি ঘ) খেলা করি<br><br>অন্যান্য (উলেখ কর্ন)________________________	আমি কিছু করিনা,ও নিজে নিজে খেলতে থাকে=০,<br>একটি উত্তর বা টিকের জন্য=১,<br>দুটি উত্তর বা টিকের জন্য=২,<br>দুয়ের অধিক =৩	|___|
16	আপনার বাচ্চার (নাম) হাতে কাগজ কলম দিয়ে কিছু কি শেখান বা ওকে সাথে নিয়ে কিছু করেন?<br><br>ক) দাগাদাগি করা শেখাই<br>খ) কলম ধরা শেখাই	আমি কিছু করিনা =০,<br>ও নিজে নিজে আঁকাআঁকি করে =০,<br>লেখালেখি শেখাই =০<br>একটি উত্তর বা টিকের জন্য = ১,<br>দুটি উত্তর বা টিকের জন্য =২, দুয়ের অধিক =৩	|___|

	গ) কাগজ দিয়ে কিছু বানানো শেখাই<br>ঘ) রংয়ের নাম শেখাই<br>ঙ) আদরকরি/প্রশংসা করি<br>অন্যান্য (উলেখ কর—ন) ______________________		
17	আপনার বাচ্চাকে (নাম) কাপড় পড়ানোর সময় কি কি করেন বা শেখান ?<br><br>ক) বিভিন্ন জিনিষের নাম (যেমন; শরীরের বিভিন্ন অংশের নাম, কাপড়ের নাম, রংয়ের নাম ইত্যাদি) শেখাই<br>খ) কাপড় পরা শেখাই,<br>গ) আদর করি/ প্রশংসা করি<br>ঘ) গল্প/ছড়া/ গান/ কবিতা বলি<br>ঙ) খেলা করি/ অভিনয় করি<br>অন্যান্য (উলেখ কর—ন) ____________________	কিছু করিনা বা শেখাইনা =০<br>একটি উত্তর বা টিকের জন্য = ১,<br>দুটি উত্তর বা টিকের জন্য =২,<br>দুয়ের অধিক =৩	
18	আপনার বাচ্চাকে (নাম) গোসল করাবার সময় ওকে নিয়ে কি কি করেন এবং ওকে কি কি শেখান ?<br><br>ক) বিভিন্ন জিনিষের নাম (যেমন; শরীরের অংশের নাম , মগ / বালটির নাম,কাপড়ের নাম ইত্যাদি)শেখাই<br>খ) ময়লা / পরিস্কার নিয়ে কথা বলি/ গরম- ঠান্ডা ইত্যাদি নিয়ে কথা বলি<br>গ) খেলা করি/ অভিনয় করি,<br>ঘ) গান করি/ছড়া/গল্প/ কবিতা বলি<br><br>অন্যান্য (উলেখ কর—ন):______________________	কিছু করিনা বা শেখাইনা =০,<br>একটি উত্তর বা টিকের জন্য= ১,<br>দুটি উত্তর বা টিকের জন্য =২,<br>দুয়ের অধিক =৩	
19	আপনার বাচ্চাকে (নাম) কি বাইরে বেড়াতে নিয়ে যান বা ঘুরাতে নিয়ে যান, তখন ওর সাথে কি কি করেন বা ওকে কি কি শেখান ?<br><br>ক) বিভিন্ন জিনিষ দেখিয়ে নাম (যেমন; আশেপাশের মানুষের, জীব জন্তুর নাম ইত্যাদি) শেখাই<br>খ) খেলা করি<br>গ) আদর করি/ প্রশংসা করি<br>ঘ) গল্প/ কবিতা/ গান করি<br>ঙ) আদব কায়দা শেখাই<br>চ) দিন/রাত,ঠান্ডা/গরম,আলো/অন্ধকার সম্পর্কে ধারনা দেই<br><br>অন্যান্য (উলেখ কর—ন) ______________________	কিছু করি না বা শেখাইনা =০<br>একটি উত্তর বা টিকের জন্য = ১ ,<br>দুটি উত্তর বা টিকের জন্য =২,<br>দুয়ের অধিক =৩	
20	বাচ্চাকে যখন কিছু শেখাতে চাচ্ছেন, তখন যদি সে ঐ কাজটা না করে অন্য কিছু করতে চায়, তখন আপনি কি করেন ?<br><br>ক) আদর করে শিখাই/ ভালোবেসে/ ধৈর্য্য ধরি<br>খ)মজার মজার কথা বলে বাচ্চার মনোযোগ আকর্ষন করার চেষ্টা করি<br>গ) খেলার মাধ্যমে আনন্দ দিয়ে বাচ্চার মনোযোগ আকর্ষন করার চেষ্টা করি<br>ঘ) ও যেটা করতে চায় সেটা নিয়ে তাকে শেখানোর জন্য চেষ্টা করি	এমনি ছেড়ে দেওয়া / জোর করা/মার দেওয়া/বকা দেওয়া = ০,<br>জোর করা/মার দেওয়া/বকা দেওয়া + অন্য যে কোন উত্তর =০<br>একটি উত্তর বা টিকের জন্য = ১,<br>দুটি উত্তর বা টিকের জন্য =২,<br>দুইয়ের অধিক =৩	

	অন্যান্য (উলেখ করুন) ___________________		

International Centre for Diarrhoeal Disease Research, Bangladesh (icddr,b)
Maternal and Child Health Division (MCHD)
Project title: **Unconditional Lactational Allowance + Pyshosocial Stimulation**
UCT Baseline forms: ID No | __ | __ |__ | __ | __ |__ |

Household Food Insecurity Access Scale (HFIAS)

নির্দেশনা ঃ টেবিলে দেওয়া প্রতিটি প্রশ্ন বিগত চার সপ্তাহ (৩০দিন) কে মনে করিয়ে দেয়ার জন্য করা হবে। যেমন প্রশ্নের বিষয়গুলো বিগত চার সপ্তাহে উত্তরদাতার সাথে কি ঘটেছে?(হ্যাঁ বা না) যদি উত্তরদাতা কোন একটি প্রশ্নের বিষয়বস্তুর উত্তরে 'হ্যাঁ' বলে, ঐ বিষয়বস্তু/ ঘটনা নিয়ে আরো কয়েকটি প্রশ্ন করতে হবে এটা নির্ণয়ের জন্য যে ঘটনাটি খুব কম (একবার বা দুইবার), মাঝে মাঝে (তিন থেকে দশ বার) অথবা প্রায়ই (১০ বারের বেশী) বিগত চার সপ্তাহের মধ্যে ঘটেছে কিনা।

উদাহরন ঃ

১। বিগত চার সপ্তাহে ঘরে পর্যাপ্ত পরিমান খাবার না থাকার কারনে আপনি কি চিন্তিত ছিলেন ?

০= না (প্রশ্ন ২ এ চলে যান)
১= হ্যাঁ

১ক। বিগত চার সপ্তাহে এরকম কতবার হয়েছে ?

১= খুব কম,(বিগত চার সপ্তাহে একবার বা দুইবার)
২= মাঝে মাঝে (বিগত চার সপ্তাহে তিন থেকে দশ বার)
৩= প্রায়ই(বিগত চার সপ্তাহে ১০ বারের বেশী)

নং	প্রশ্ন	উত্তর	কোড
১.	বিগত চার সপ্তাহে ঘরে পর্যাপ্ত পরিমান খাবার না থাকার কারনে আপনি কি চিন্তিত ছিলেন?<br>(In the past four weeks, did you worry that your household would not have enough food?)	না = ০<br>হ্যাঁ = ১	
১ক.	বিগত চার সপ্তাহে এরকম কতবার হয়েছে ? (না হলে ২ এ চলে যান)<br>(How often did this happen in the past four weeks?)	খুব কম=১<br>মাঝেমাঝে=২<br>প্রায়ই=৩	

২.	বিগত চার সপ্তাহে আর্থিক অভাব অনটনের কারনে আপনি বা আপনার পরিবারের কোন সদস্য পছন্দের খাবার খেতে পারেননি ?<br>(In the past four weeks, were you or any household member not able to eat the kinds of foods you preferred because of a lack of resources?)	না = ০<br>হ্যাঁ = ১	
২ক.	বিগত চার সপ্তাহে এরকম কতবার হয়েছে ? (না হলে ৩ এ চলে যান)<br>(How often did this happen in the past four weeks?	খুব কম=১<br>মাঝেমাঝে=২<br>প্রায়ই=৩	
৩.	বিগত চার সপ্তাহে আর্থিক অভাব অনটনের কারনে আপনি বা আপনার পরিবারের কোন সদস্য সীমিত ধরনের খাবার খেয়েছেন ?)<br>In the past four weeks, did you or any household member have to eat a limited variety of foods due to a lack of resources?	না = ০<br>হ্যাঁ = ১	
৩ক.	বিগত চার সপ্তাহে এরকম কতবার হয়েছে ? (না হলে ৪ এ চলে যান)<br>How often did this happen in the past four weeks?	খুব কম=১<br>মাঝেমাঝে=২<br>প্রায়ই=৩	
নং	প্রশ্ন	উত্তর	কোড
৪.	বিগত চার সপ্তাহে আর্থিক অভাব অনটনের কারনে অন্য খাবার কিনতে না পেরে আপনি বা আপনার পরিবারের কোন সদস্য এমন কোন খাবার খেয়েছেন যা আসলে আপনারা খেতে চাননা ?<br>(In the past four weeks, did you or any household member have to eat some foods that you really did not want to eat because of a lack of resources to obtain other types of food?)	না = ০<br>হ্যাঁ = ১	
৪ক.	বিগত চার সপ্তাহে এরকম কতবার হয়েছে ? (না হলে ৫ এ চলে যান)<br>(How often did this happen in the past four weeks?)	খুব কম=১<br>মাঝেমাঝে=২<br>প্রায়ই=৩	
৫.	বিগত চার সপ্তাহে যথেষ্ঠ পরিমান খাবার না থাকার কারনে আপনি বা আপনার পরিবারের কোন সদস্য প্রয়োজনের তুলনায় অল্প পরিমাণ খাবার খেয়েছেন কি?<br>(In the past four weeks, did you or any household member have to eat a smaller meal than you felt you needed because there was not enough food?)	না = ০<br>হ্যাঁ = ১	
৫ক.	বিগত চার সপ্তাহে এরকম কতবার হয়েছে ? (না হলে ৬ এ চলে যান)<br>(How often did this happen in the past four weeks?)	খুব কম=১<br>মাঝেমাঝে=২<br>প্রায়ই=৩	
৬.	বিগত চার সপ্তাহে যথেষ্ঠ পরিমান খাবার না থাকার কারনে আপনি বা আপনার পরিবারের কোন সদস্য অন্তত কোন এক বেলা খাবার কম খেয়েছেন কি?<br>(In the past four weeks, did you or any household member have to eat fewer meals in a day because there was not enough food?)	না = ০<br>হ্যাঁ = ১	
৬ক.	বিগত চার সপ্তাহে এরকম কতবার হয়েছে ? (না হলে ৭ এ চলে যান)<br>(How often did this happen in the past four weeks?)	খুব কম=১<br>মাঝেমাঝে=২<br>প্রায়ই=৩	
৭.	বিগত চার সপ্তাহে আর্থিক অভাব অনটনের কারনে খাবার কিনতে না পারায়, আপনার ঘরে কি খাবার উপযোগী কোন খাবার ছিলনা ?<br>(In the past four weeks, was there ever no food to eat of any kind in your household because of lack of resources to get food?	না = ০<br>হ্যাঁ = ১	

icddr,b KNOWLEDGE FOR GLOBAL LIFESAVING SOLUTIONS

Grand Challenges Canada Grands Défis Canada BOLD IDEAS WITH BIG IMPACT

৭ক.	বিগত চার সপ্তাহে এরকম কতবার হয়েছে ? (না হলে ৮ এ চলে যান)<br>(How often did this happen in the past four weeks?)	খুব কম=১<br>মাঝেমাঝে=২<br>প্রায়ই=৩	
৮.	বিগত চার সপ্তাহে আপনি বা আপনার পরিবারের কোন সদস্য প্রয়োজনীয় খাবারের অভাবে ক্ষুধা পেটে রাতে ঘুমিয়েছেন কি?<br>(In the past four weeks, did you or any household member go to sleep at night hungry because there was not enough food?)	না = ০<br>হ্যাঁ = ১	
৮ক.	বিগত চার সপ্তাহে এরকম কতবার হয়েছে ? (না হলে ৯ এ চলে যান)<br>(How often did this happen in the past four weeks?)	খুব কম=১<br>মাঝেমাঝে=২<br>প্রায়ই=৩	
৯.	বিগত চার সপ্তাহে প্রয়োজনীয় খাবারের অভাবে আপনি বা আপনার পরিবারের কেউ সারা দিনে ও রাতে কিছুই খাননি- এমন হয়েছে কি?<br>(In the past four weeks, did you or any household member go a whole day and night without eating anything because there was not enough food?)	না = ০<br>হ্যাঁ = ১	
৯ক.	বিগত চার সপ্তাহে এরকম কতবার হয়েছে ? (প্রশ্ন এখানেই শেষ করুন)<br>(How often did this happen in the past four weeks?)	খুব কম=১<br>মাঝেমাঝে=২<br>প্রায়ই=৩	

International Centre for Diarrhoeal Disease Research, Bangladesh (icddr,b)
Maternal and Child Health Division (MCHD)
Project title: **Unconditional Lactational Allowance + Pyshosocial Stimulation**

Family Care Indicators

Mother's name:	Child's Name:
Child's ID: / _ / _ / _ / _ / _ / _ / _ / _ / _ /	DOT (DD/MM/YY) ☐☐/☐☐/☐☐
Interviewer's Name:	

তথ্য প্রদানকারী মা ব্যতিত অন্যকেউ হলে তার নাম:

"আমি সে সমস্ত জিনিস সম্পর্কে জানতে আগ্রহী যা দিয়ে (নাম) বাড়িতে খেলা করে।" দয়া করে আমাকে সেগুলো দেখাবেন। খেলনাগুলো বাড়ীতে তৈরী, যেমনঃ ঘরের তৈরী মাটির খেলনা, কাপড়ের তৈরী পুতুল অথবা কেনা খেলনা এবং বাড়ির জিনিসপত্র ইত্যাদিও হতে পারে।

প্রশ্নগুলো কোড করতে হবে।মা যখন খেলনাগুলো দেখাবে, প্রশ্নগুলো মাকে বাড়িতে আছে এমন অন্যান্য খেলনার কথাও মনে করিয়ে দিবে।যে সমস্ত খেলনা মা দেখাতে পারবে শুধু সেগুলোই কোড করুন।শুধু খেলনা থাকলেই হবে না, সেই খেলনা দিয়ে নির্দিষ্ট প্রশ্নের খেলা খেলতে হবে বা নির্দিষ্ট প্রশ্নের কাজের জন্য ব্যবহার করতে হবে

No	Question	Response	Code
1	গত ৩০ দিনে (নাম) এমন কোন খেলনা দিয়ে খেলেছে যাতে বাজনা হয় বা বাজানো যায়, (যেমন বাদ্যযন্ত্র অথবা যে সব খেলনা শব্দ তৈরী করে, যেমন; বাচ্চার খেলার জন্য প্লাস্টিকের মোবাইল, খেলনা রেডিও, গান গাওয়ার পুতুল, ঢোল, বাঁশি, ইত্যাদি?) (In the last 30 days(Child name) did the	1= হ্যাঁ<br>0= না	

	child play with any toy that can make music or can be played as musical instrument (e.g. musical instrument or the toys that produce musical sound e.g. plastic mobile as toy, radio as toy, singing doll, tom-tom, pipe etc.) ?		
2	গত ৩০ দিনে (নাম) আঁকাআঁকি বা লেখা যায় এমন জিনিস দিয়ে খেলেছে (যেমন: রং করার জন্যে ছবির বই, পেন্সিল, কলম, চক,শ্লেট, লেখা অথবা কাঠি দিয়ে উঠানের মাটিতে/ ঘরের মেঝেতে দাগাদাগি, ইত্যাদি)? (In the last 30 days did the child (name) play with any toy that can be used for drawing or writing purpose (e.g. picture book for coloring, pencil, pen, chalk, slate or marking / writing with stick in the floor or courtyard etc.)	1= হ্যাঁ<br>0= না	
3	বাচ্চাদের উপযোগী কোন ছবির বই আছে (স্কুল বই ছাড়া)?<br>(Is there any picture book suitable for the child (except school book?)	1= হ্যাঁ<br>0= না	
4	গত ৩০ দিনে (নাম) এমন কোনো জিনিস দিয়ে কোন কিছু সেজে বা অভিনয় করে খেলেছে, যেমন-মা, ডাক্তার, শিক্ষক, নায়ক, পুতুল, খাওয়ার জন্য প্লেট এবং কাপ, ইত্যাদি?) (In the last 30 days did the child (name) play with anything that disguise himself or take the role of mother, doctor, teacher, actor, doll, plate & cup for acting purpose?)	1= হ্যাঁ<br>0= না	
5	গত ৩০ দিনে (নাম) ছুটাছুটি করে খেলতে পারে এমন কোনো খেলনা দিয়ে খেলেছে (যেমন বল, দড়ি লাফানো, ব্যাট, দড়ি দিয়ে বানানো দোলনা, টানা বা ঠেলা দেওয়া গাড়ী, ইত্যাদি)? (In the last 30 days(Child name) did the child play with any toy with which he ran about (e.g. ball& bat, rope for jumping, rocking cradle made of rope, a car that can be pulled or pushed etc ?)	1= হ্যাঁ<br>0= না	
6	বাচ্চার এমন কোন খেলনা আছে যা দিয়ে বিভিন্ন আকৃতি (তিনকোণা, চারকোণা, গোল) এবং রং শেখা যায় (Does the child have any toy with which he can get idea regarding shape (triangular, rectangular, round) & color?)	1= হ্যাঁ<br>0= না	

No	Question	Response	Code
7	বাচ্চার এমন কোন খেলনা আছে (যেমন-গুটি,লেগো, ব্লক) যা একটার উপর আরেকটা রেখে, বা পাশাপাশি রেখে টাওয়ার,ঘর, গাড়ী ইত্যাদি বানানো যায় ?<br>(Does the child have any toy (globular shaped, logo, block) with which tower; house, car etc can be made by placing them one over another or side by side.)	1= হ্যাঁ<br>0= না	
নির্দেশনা: 8 ও 9 নং প্রশ্নের উত্তর সংখ্যায় লিখুন। সংখ্যাটি 10 বা 10 এর বেশী হলে 10 লিখুন			
8	বাড়িতে স্কুল বই সহ কয়টি বই আছে (বাচ্চাদের ছবির বই ছাড়া) ?<br>What is the number of book in your house including school book (except picture book for children)……..		
9	বাড়িতে কয়টি ম্যাগাজিন এবং পেপার আছে?<br>What is the number of paper & magazine at your house?		
এখন আপনার কাছে কিছু কাজ বা খেলার কথা জানতে চাইব যা আপনি অথবা বাবা বা বাড়ীর বড় কেউ বাচ্চার সাথে গত ৩ দিনে করেছে কিনা জানাবেন?			
10.	বই পড়ে শুনিয়েছেন,ছবির বই /ছবি / পোষ্টার দেখিয়েছেন-<br>10(1) মা 10(2) বাবা 10(3) পরিবারের অন্যকেউ যার বয়স ১৫ বছরের উপরে<br>(Book was read or picture book/picture/poster was shown to your child by-<br>1.Mother 2. Father 3. other family member who is above 15 years of old)		

11	বাচ্চাকে (নাম) গল্প বলেছেন-<br>11(1) মা 11(2) বাবা 11(3) পরিবারের অন্যকেউ যার বয়স ১৫ বছরের উপরে<br>(Story was told to the child (name) by-<br>1.Mother 2. Father 3. other family member who is above 15 years of old)		
12	গান / ছড়া গান,গজল গেয়েছেন -<br>12(1) মা 12(2) বাবা 12(3) পরিবারের অন্যকেউ যার বয়স ১৫ বছরের উপরে<br>(Song, rhyme, religious song was sung to the child (name) by-<br>1.Mother 2. Father 3. other family member who is above 15 years of old)		
13	বাচ্চার (নাম) সাথে খেলনা দিয়ে খেলেছেন<br>13(1) মা 13(2) বাবা 13(3) পরিবারের অন্যকেউ যার বয়স ১৫ বছরের উপরে<br>(Game was played with the child (name) using toys by-<br>1.Mother 2. Father 3. other family member who is above 15 years of old)		
14	বাচ্চাকে (নাম) সময় দিয়ে কোন কিছুর নাম, গনণা এবং আঁকাআকি শিখিয়েছেন-<br>14(1) মা 14(2) বাবা 14(3) পরিবারের অন্যকেউ যার বয়স ১৫ বছরের উপরে<br>(*Name of* something, counting number & drawing was taught to the child (name) by allocating time for him by-<br>1. Mother 2. Father 3. other family member who is above 15 years of old)		

International Centre for Diarrhoeal Disease Research, Bangladesh (icddr,b)
Maternal and Child Health Division (MCHD)
Project title: **Unconditional Lactational Allowance + Pyshosocial Stimulation**
Quality of Life questionnaire
উত্তর দাতার জীবন যাত্রার গুনগত মান

নীচের প্রশ্নসমূহ আপনার জীবনের গুনগত মান, স্বাস্থ্য ও অন্যান্য বিষয়গুলি সম্পর্কে আপনার অনুভূতি বিশ্লেষণ করছে। অনুগ্রহ করে আপনার অনুভূতি বিবেচনা করে সকল প্রশ্নের উত্তর দিন, যা স্বাক্ষাতপ্রার্থী গোল চিহ্ন দিয়ে লিপিবদ্ধ করবেন। কোন একটি প্রশ্ন সম্পর্কে অনিশ্চিত হলেও যা সবচাইতে যথাযথ মনে হয় বেছে নিন।অনুগ্রহপূর্বক আপনার জীবনের গুনগত মান, আশা, তুষ্টি ও গত ৪ সপ্তাহে জীবনের সাথে সংশ্লিষ্ট বিষয়াদির দিকে লক্ষ্য রাখুন।

	প্রশ্ন	খুব নিম্ন	নিম্ন	নিম্ন নয় ভাল ও নয়	ভাল	খুব ভাল	উত্তর
১।	আপনার জীবন যাত্রার গুনগত মান কেমন মুল্যায়ন করেন?<br>How would you rate your quality of life?	১	২	৩	৪	৫	

		খুব অসন্তুষ্ট	অসন্তুষ্ট	সন্তুষ্ট বা অসন্তুষ্ট নই	সন্তুষ্ট	খুব সন্তুষ্ট	

২।	আপনার স্বাস্থ্য সম্পর্কে কেমন সন্তুষ্ট?<br>How satisfied are you with your health?	১	২	৩	৪	৫	

		মোটেই না	স্বল্প	মোটামুটি পরিমান	খুব বেশী	অত্যধিক পরিমান	
৩।	আপনি যা করতে চান, শারীরিক ব্যাথা তা থেকে আপনাকে কি পরিমান বাধাগ্রস্ত করে?<br>To what extent do you feel that physical pain prevents you from doing what you need to do?	৫	৪	৩	২	১	
৪।	দৈনন্দিন জীবন নির্বাহে আপনার চিকিৎসা সহায়তা কতটুকু প্রয়োজন?<br>How much do you need any medical treatment to function in your daily life?	৫	৪	৩	২	১	
৫।	জীবনকে কতটুকু উপভোগ করেন?<br>How much do you enjoy life?	১	২	৩	৪	৫	
৬।	আপনি আপনার জীবনকে কতখানি অর্থবহ মনে করেন?<br>To what extent do you feel your life to be meaningful?	১	২	৩	৪	৫	

		মোটেই না	সামান্য	মোটামুটি	যথেষ্ট	অত্যধিক	উত্তর
৭।	আপনি মনোযোগ দিতে কতটুকু সক্ষম<br>How well are you able to concentrate?	১	২	৩	৪	৫	
৮।	আপনি আপনার দৈনন্দিন জীবনে কেমন নিরাপদ মনে করেন?<br>How save do you feel in your daily life?	১	২	৩	৪	৫	

		মোটেই না	স্বল্প	মোটামুটি	প্রায়শঃই	সম্পূর্ণ	
৯।	আপনাদের ভৌত পরিবেশ কেমন স্বাস্থ্যকর?<br>How healthy is your physical environment?	১	২	৩	৪	৫	
১০।	প্রাত্যহিক জীবন যাপনের জন্য আপনার যথেষ্ট শক্তি আছে কি?<br>Do you have enough energy for everyday life?	১	২	৩	৪	৫	
১১।	আপনি আপনার শারীরিক অবয়ব মেনে নিতে পারেন কি?<br>Are you able to accept your bodily appearance?	১	২	৩	৪	৫	
১২।	আপনার অর্থনৈতিক চাহিদা মিটানোর জন্য যথেষ্ট টাকা পয়সা আছে কি?<br>Have you enough money to meet your needs?	১	২	৩	৪	৫	
১৩।	আপনি দৈনন্দিন যাবতীয় দরকারী তথ্যাদি পান কি?<br>How available to you is the information that you need in your day to day life?	১	২	৩	৪	৫	
১৪।	আপনার অবসর উপভোগের কেমন সুযোগ আছে ?<br>To what extent do you have the opportunity for leisure activities?	১	২	৩	৪	৫	

		খুব খারাপ	খারাপ	খারাপ ও নয় ভালও নয়	ভাল	খুব ভাল	
১৫।	আপনি চলাফেরা করতে কতটুকু সক্ষম?<br>How well are you able to get around?	১	২	৩	৪	৫	

		খুব অসন্তুষ্ট	অসন্তুষ্ট	সন্তুষ্ট বা অসন্তুষ্ট নই	সন্তুষ্ট	খুব সন্তুষ্ট	
১৬।	আপনার ঘুম নিয়ে আপনি কেমন সন্তুষ্ট?<br>How satisfied are you with your sleep?	১	২	৩	৪	৫	
১৭।	আপনি আপনার দৈনন্দিন জীবন প্রবাহের জন্য কার্যক্ষমতায় কেমন সন্তুষ্ট?<br>How satisfied are you with your ability to perform your daily living activities?	১	২	৩	৪	৫	
১৮।	আপনার কর্মক্ষমতা সম্পর্কে আপনি কতটুকু সন্তুষ্ট?<br>How satisfied are you with your capacity for work?	১	২	৩	৪	৫	
১৯।	নিজেকে নিয়ে আপনি কেমন সন্তুষ্ট?<br>How satisfied are you with yourself?	১	২	৩	৪	৫	
২০।	আপনি আপনার ব্যক্তিগত সম্পর্ক নিয়ে কেমন সন্তুষ্ট?<br>How satisfied are you with your personal relationships?	১	২	৩	৪	৫	
২১।	আপনার যৌনজীবন নিয়ে আপনি কত সন্তুষ্ট? (বিবাহিতদের জন্য)<br>How satisfied are you with your sex life?	১	২	৩	৪	৫	
২২।	আপনি আপনার বন্ধুদের কাছ থেকে যে সহায়তা পান তাতে সন্তুষ্ট কি?<br>How satisfied are you with the support you get from your friends?	১	২	৩	৪	৫	
২৩।	আপনার বাসস্থানের অবস্থা সম্পর্কে আপনি কেমন সন্তুষ্ট?<br>How satisfied are you with the conditions of your living place?	১	২	৩	৪	৫	
২৪।	আপনি উপযুক্ত সাস্থ্যসেবার প্রাপ্ত সুযোগ নিয়ে কেমন সন্তুষ্ট?<br>How satisfied are you with your access to health service?	১	২	৩	৪	৫	
২৫।	আপনার পরিবহন সুবিধা নিয়ে আপনি কেমন সন্তুষ্ট?<br>How satisfied are you with your transport?	১	২	৩	৪	৫	

		কখোনো নয়	কদাচিৎ	প্রায়শঃই	প্রায় সবসময়	সবসময়	
২৬।	কত ঘন ঘন আপনি হতাশ, উদ্বেগ,উদ্যমহীনতা, বিপদগ্রস্ততার মত নেতিবাচক লাভ করেন?<br>How often do you have negative feelings such as blue mood, despair, anxiety, depression?	৫	৪	৩	২	১	

International Centre for Diarrhoeal Disease Research, Bangladesh (icddr,b)
Maternal and Child Health Division (MCHD)
Project title: **Unconditional Lactational Allowance + Pyshosocial Stimulation**
Domestic Violence Questionnaire

Name of child: ____________________________ Child Id:
Name of Mother: ____________________________ Date of interview: / / /

SL	প্রশ্ন (Question)	উত্তর(Answer)	Code	মন্তব্য
1	গত মাসে পরিবারের কোন সদস্য দ্বারা আপনি দৈহিকভাবে নির্যাতনের শিকার হয়েছেন?(Have you been physically abused in the last month by your family member?)	হ্যাঁ (Yes)=1<br>না (No)=2<br>জানিনা (Do not know)=99		
2	যদি হ্যাঁ হয়, গত মাসে পরিবারের সদস্য দ্বারা দৈহিকভাবে নির্যাতনের ধরন উল্লেখ করুন (আঘাত করা, চড়-থাপ্পর,লাথি মারা,ধাক্কা দেওয়া/ছুঁড়ে দেওয়া বা অন্যান্য)(If Yes,within the last month, would you pls mention the type of physical abuse(hit, slapped, kicked, pushed or shoved, or otherwise) by your family member)	কোন কিছু দিয়ে আঘাত (hit by anything)=1<br>চড়-থাপ্পর(slapped)=2<br>লাথি মারা(kicked)=3<br>ধাক্কা দেওয়া/ছুঁড়ে দেওয়া গুতা মারা (pushed=4<br>(shoved)=5<br>অন্যান্য(উল্লেখ করুন) =6(pls mention)...............		
3	কার দ্বারা (by whom)	স্বামী (Huband)=<br>অন্যান্য(সম্পর্ক লিখুন) Other =(Write relation)......		
4	কত বার হয়েছে (Number of times)________________	কতদিন পর পর এরকম হয় (Frequency)_______		
5	আপনার শরীরের কোন জায়গায় তিনি দৈহিক আঘাত করা হয়েছে (Where is the place of your body he/she physically asult you)			

6	আপনি কি দৈহিক নির্যাতনের জন্য চিকিৎসক/কবিরাজের কাছে গিয়েছিলেন (Have you visited doctor/kabiraj for this physical assult)	হ্যাঁ (Yes)<br>না (No)<br>জানিনা (Do not know)		
7	গত মাসে পরিবারের কোন সদস্য দ্বারা আপনি মানষিক ভাবে নির্যাতনের শিকার হয়েছেন(Have you been mentally/emotionally abused in the last month by your family member ?)	হ্যাঁ (Yes)<br>না (No)<br>জানিনা (Do not know)		
8	মানষিক নির্যাতনের ধরন উল্লেখ করুনType of mental or physical abuse (pls mention)			
9	যদি হ্যাঁ হয়, কার দ্বারা (If YES, by whom)	স্বামী (Huband)<br>অন্যান্য(সম্পর্ক লিখুন) Other (Write relation)......		
10	কত বার হয়েছে (Number of times)	কতদিন পর পর এরকম হয়(Frequency)________		
11	গত মাসে আপনার স্বামী কি জোর করে সহবাস করেছে(Does your husband in the last month force you into sex)	হ্যাঁ (Yes)<br>না (No)<br>জানিনা (Do not know		
12	আপনি কি পরিবারের সদস্যদের ভয় পান (Are you afraid of your family members?)	হ্যাঁ (Yes)<br>না (No)<br>জানিনা (Do not know		
13	যদি হ্যাঁ হয়, তবে কার (If yes for whom)	স্বামী (Huband)<br>অন্যান্য(সম্পর্ক লিখুন) Other (Write relation)......		
14	আপনার পরিবারের কোন সদস্যকে কি আপনি বিপদজনক মনে করেন (Do you feel peril from any of your family member)	হ্যাঁ (Yes)<br>না (No)<br>জানিনা (Do not know		
15	যদি হ্যাঁ হয়, তবে কার(If yes for whom)	স্বামী (Huband)<br>অন্যান্য(সম্পর্ক লিখুন) Other (Write relation)......	1<br>2	

International Centre for Diarrhoeal Disease Research, Bangladesh (icddr,b)
Maternal and Child Health Division (MCHD)
Project title: **Unconditional Lactational Allowance + Pyshosocial Stimulation**

Expenditure and income questionnaire

Name of child: ___________________________ Child Id:
Name of Mother: ____________________________ Date of interview: / / /

এখন আমি আপনার মাসিক আয়-ব্যয় বিষয়ে জিজ্ঞাসা করবো আপনি দয়া করে নীচের প্রশ্নগুলোর উত্তর দিবেন।(Now I will ask you about your monthly expenditure and income. Would you please answer me the bellow questions?)

Monthly expenditure questionnaire

Sl	Questionnaire	Total Tk.	Remark
1	গত ৩০দিনে আপনার খাবারের জন্য কত খরচ হয়েছে?(Food expenditure cost for last 30 days)		
2	গত ৩০দিনে কাপড় ও জুতার জন্য আপনার কত খরচ হয়েছে?(Clothing and footwear cost for last 30 days)		
3	বাচ্চাদের পড়াশুনার জন্য কত খরচ হয়েছে? (Children's education cost for last 30 days)		
4.	খেলনা, বই-পত্র,বিনোদন শুধুমাত্র আপনার ৫বছরের নীচের বাচ্চার জন্য কত খরচ হয়েছে (দয়া করে উল্লেখ করুন) Cost for toys, books, recreation (visit) only for children under 5 years old(pls mention)<br>1.খেলনা(Toys)___________<br>2.বই-পত্র(Books)______________<br>3. বিনোদন(ভ্রমণ/দেখতে যান) Recreation(visit)_________<br>4. অন্যান্য.(নাম ও খরচ)____________		
5	গত ৩০দিনে ঘরের আসবাবপত্র ও সরঞ্জামাদিতে কত খরচ হয়েছে? (Household furniture and utensils cost for last 30 days (plate, glass, chair, mirror, cosmetics etc)		
6	গত ৩০দিনে ঘরে ভোগ্য দ্রব্যাদিতে কত খরচ হয়েছে?(Household consumables cost for last 30 days (soaps, cigarettes, mobile bill, wood, others)		
7	গত ৩০দিনে যাতায়াত বাবদ কত খরচ হয়েছে?(Households transport cost for last 30 days)		
8	গত ৩০দিনে গৃহপালিত পশুর জন্য কত খরচ হয়েছে?(Household expenditure on domestic animals for last 30 days)		
9	গত ৩০দিনে স্বাস্থ্য সেবায় কত খরচ হয়েছে?(Health care expenditure for last 30 days)<br>1.পরামর্শ খরচ(Consultation fee)_______________<br>2.ডায়াগনষ্টিক খরচ(Diagnostic cost)_________________<br>3.ঔষধ খরচ(Medicine cost)____________________<br>4. পরিবহন/ যাতায়াত খরচ (যদি হেলথ্ কেয়ার প্রোভাইডারকে দেখা করার জন্য কোন খরচ হয় তাহলে প্রশ্ন ৬ উপেক্ষা করুন)(Transport cost(pls avoide Q 6 if cost is for visit of a health care provider)____________________<br>5. অন্যান্য.___________		

10	যে কোন ঋণ সংক্রান্ত খরচ (কিস্তি, মাইক্রোক্রেডিট ইত্যাদি) (Any cost related to debt (instalment, microcredit etc)pls write down-------		
11	গত ৩০দিনে অন্যান্য খরচ (উল্লেখ করুন) (Other cost for last 30 days (please mention) ………………………………………		

Monthly expenditure and income questionnaire

	Item	BDT	Remark
12	গত ৩০দিনে কাজের মাধ্যমে আয় (মাসিক বেতন, দিন মজুরী ইত্যাদি) (Income through Jobs (monthly salaried, daily labour etc) for last 30 days)		
13	গত ৩০দিনে ফসল, ফল-মূল, হাঁস-মুরগী,পোল্ট্রি দ্রব্যাদি ইত্যাদি বিক্রি দ্বারা আয় (Income through selling regular crops, fruits, poultry, poultry product etc for last 30 days)		
14	গত ৩০দিনে শর্তহীন নগদ অর্থ পাওয়ার দ্বারা আয় (Income through unconditional cash for last 30 days)<br>1. গর্ভকালীন এবং দুগ্ধদানকারী মায়েদের ভাতা Pregn. and lactating allowance)________<br>2. সরকারী অন্যান্য ভাতাদি উল্লেখ বরুন Govt. other allowance(pls mention)_______<br>3. অন্যান্য (আত্নীয়, নেতাদের থেকে ইত্যাদি)Others(relatives, leaders etc)________		
15	গত ৩০দিনে ভাড়া থেকে কোন আয়(Income through rent for last 30 days)		
16	গত ৩০দিনে জমা থেকে আয় ।(দয়া করে উল্লেখ করুন (Income received as debt for last 30 days).Pls mention -----------<br>1.ক্ষুদ্র ঋণ (Microcredit) ____<br>2. ব্যাংকের সুদ (Bank interest)_____<br>3. অন্যান্য (Others)............		
17	গত ৩০দিনে অন্যান্য আয় (উল্লেখ করুন) Others income for last 30 days (pls mention)		

18. আপনার বর্তমান অর্থনৈতিক অবস্থায় কতটা সন্তুষ্ট ?
(How satisfactory is your current economic situation)

1.খুব ভাল(Very good)	2. ভাল (Good)	3. সাধারন (Normal)
4.ভাল নয় (Not very good)	5.খুব খারাপ (Very bad)	

19. আপনার পরিবারের আয় কমপক্ষে কত হলে ভাল/সচ্ছল ভাবে জীবন যাপন করা যাবে বলে মনে করেন ? (For your household, what do you consider to be the minimum monthly income with which you can live well?)

__________ টাকা (BDT

icddr,b KNOWLEDGE FOR GLOBAL LIFESAVING SOLUTIONS

Grand Challenges Canada Grands Défis Canada BOLD IDEAS WITH BIG IMPACT

International Centre for Diarrhoeal Disease Research, Bangladesh (icddr,b)
Maternal and Child Health Division (MCHD)
Project title: **Unconditional Lactational Allowance + Pyshosocial Stimulation**
Health seeking behaviour questionnaire

Name of child: ______________________ Child Id:
Name of Mother: ______________________ Date of interview: / / /

প্রশ্ন	উত্তর	কোড
১। গত ১ মাসে আপনি শারীরিকভাবে অসুস্থ হয়েছেন কি ?	১. হ্যা ২. না	
১(ক)হ্যা হলে কোন্ ধরনের সমস্যা	১ জ্বর ২ কাশি ৩.পাতলা পায়খানা ও বমি ৪. শরীর ব্যথা ৫. মাথা ব্যথা ৬. পেটে ব্যথা ৭. ইনজুরী ৮.শরীরে বল পায়না ৯. চোখে ঝাপসা দেখা ১০.অন্য অসুখ উল্লেখ করুন...	
২। গত ১ মাসে কোন শারীরিক অসুবিধার কারণে ডাক্তার বা কবিরাজ দেখিয়েছেন কি ?	১. হ্যা ২. না	
৩। কো্থায় দেখিয়েছেন	১. সরকারী স্বাস্থ্যকেন্দ্র, ২. এন জি ও স্বাস্থ্যকেন্দ্র ৩. প্রাইভেট ক্লিনিক/হাসপাতাল, ১৩. অন্যান্য-----------	
৪ ।কাকে দেখিয়েছেন	১. রেজিস্টার্ড ডাক্তার<br>২.মেডিকেল এসিস্ট্যান্ট/নার্স/এফ ডাব্লিউ,ভি/প্যারামেডিক্স,<br>৬. সরকারী স্বাস্থ্যকর্মী<br>৭. হোমিওপ্যাথিক ডাক্তার<br>৮. ফার্মেসী ম্যান,<br>৯.পল্লী চিকিৎসক<br>১০. পীর /ফকির / ওঝা, কবিরাজ/৫<br>১১. নিজে নিজেই, ১২. আত্মীয় স্বজন,	
৫। কত টাকা খরচ হয়েছে	১.ডাক্তার ফি ------<br>২.যাতায়াত--------<br>৩.ডায়গনষ্টিক---<br>৪.ঔষধ------<br>৫.থাকা খাওয়া ---<br>৬.অন্যান্য-----------	
৬। হাসপাতালে বা ক্লিনিকে থাকতে হয়েছে কি না	১. হ্যা ২. না	
৭। টাকা কিভাবে যোগাড় করেছেন	১. নিজের জমা ছিল<br>২. ছেলে বা মেয়ে দিয়েছে<br>৩. জমি বা কোন কিছু বিক্রি করেছেন<br>৪.ঋণ করেছেন।<br>৫. অন্যান্য-----------	
৮। চিকিৎসা করানোর জন্য আপনাকে কে নিয়ে গিয়েছিল	১. ছেলে/ মেয়ে<br>২. নিজের স্ত্রী<br>৩. নাতি, পুতনি<br>৪. প্রতিবেশীরা	

	৫. অন্যান্য----	
৯। বর্তমানে আপনি নিজে আয় করেন কি না ?	১. হ্যা ২.না	
১০। হ্যা হলে মাসে কত টাকা আয় করেন		
১১। ভবিষ্যতে নিজের কোন স্বাস্থ্য সমস্যা হলে চিকিৎসা টাকা উৎস কি?	১. নিজের জমানো টাকা ২. সংসারের অন্য কারো টাকা ৩. ঋণ করা টাকা, ৪.সম্পদ বিক্রি করা টাকা ৫. অন্যান্য-----------	

International Centre for Diarrhoeal Disease Research, Bangladesh (icddr,b)
Maternal and Child Health Division (MCHD)
Project title: **Unconditional Lactational Allowance + Pyshosocial Stimulation**
SELF REPORTING QUESTIONNAIRE 20 (SRQ)

**If no response for any question, write NA as response**	
Mother's name:	Child Name:
Date (DD/MM/YY) ☐☐ / ☐☐ / ☐☐	Interviewer Name:

#	Question	00 = No<br>01 = Yes
1	গত চার সপ্তাহের মধ্যে আপনার কি প্রায়ই মাথা ব্যাথা করেছে? (Do you often have headaches?)	☐☐
2	গত চার সপ্তাহের মধ্যে আপনার কি খাবারে অরুচি হয়েছে ? (Is your appetite poor?)	☐☐
3	গত চার সপ্তাহের মধ্যে আপনার কি ঘুমে অসুবিধা হয়েছে? (Do you sleep badly?)	☐☐
4	গত চার সপ্তাহের মধ্যে আপনি কি অল্পতেই বা একটুতেই ভয় পেয়েছেন? (Are you easily frightened?)	☐☐
5	গত চার সপ্তাহের মধ্যে আপনার কি হাত কেঁপেছে? Do your hands shake?	☐☐
6	গত চার সপ্তাহের মধ্যে আপনি কি ঘাবড়িয়েছেন, দুশ্চিন্তায় থেকেছেন বা টেনশন করেছেন? (Do you feel nervous, tense or worried?)	☐☐
7	আপনার কি হজমে সমস্যা হয়েছে গত চার সপ্তাহে? (Is your digestion poor?)	☐☐
8	পরিস্কারভাবে কিছু চিন্তা করতে কি আপনার অসুবিধা হয়েছে গত চার সপ্তাহে? (Prob - আপনি একটা চিন্তা করছেন তার মাঝে আরো অনেকগুলো চিন্তা ঢুকে যাচ্ছে কি?) (Do you have trouble thinking clearly?)	☐☐
9	গত চার সপ্তাহের মধ্যে আপনার কি মনে হয়েছে যে আপনার জীবনে সুখ নাই? (Do you feel unhappy?)	☐☐
10	গত চার সপ্তাহের মধ্যে আপনি কি স্বাভাবিকের চেয়ে বেশী কান্নাকাটি করেছেন?(Do you cry more than usual?)	☐☐
11	গত চার সপ্তাহের মধ্যে আপনার প্রত্যেক দিনের কাজ কি আপনার করতে ভাল লাগে নাই? Do you find it difficult to enjoy your daily activities?	☐☐
12	গত চার সপ্তাহের মধ্যে আপনার কি কোন ব্যাপারে মন ঠিক করতে কোন অসুবিধা হয়েছে? (Do you find it difficult to make decisions?)	☐☐
13	প্রত্যেক দিন আপনি যে সব কাজ করেন সেইসব কাজে কোন ক্ষতি বা কোন অসুবিধা হয়েছে গত চার সপ্তাহে? (Prob - সবসময়েই কাজটা যেভাবে করেন সেভাবেই করেছেন নাকি ঠিকমত করতে পারেন নাই?) ? Is your daily work suffering?	☐☐
14	গত চার সপ্তাহের মধ্যে আপনার কি মনে হয়েছে যে আপনি গুরুত্বপূর্ণ কোন কিছু করতে পারছেন না ? (Prob - আপনার কাছে যেটা মনে হয় জীবন সংসারে জরুরী বা প্রয়োজনীয়, তা কি করতে পারছেন না মনে হয়েছে Are you unable to play a useful part in life?)	☐☐

15	গত চার সপ্তাহের মধ্যে আপনার কি কোন কিছুতেই মন লাগে নাই বা বিভিন্ন ব্যাপারে আগ্রহ কমে গেছে/ আগ্রহ হারিয়ে ফেলেছেন? (Have you lost interest in things?)	☐ ☐
16	গত চার সপ্তাহের মধ্যে আপনার কি মনে হয়েছে যে আপনার কোন দামই নাই? Do you feel that you are a worthless person?	☐ ☐
17	গত চার সপ্তাহের মধ্যে আপনার কি কখনও মনে হয়েছে নিজের জীবন নিজেই শেষ করে দেই? (Prob - আপনি কি নিজের জীবন নিজে শেষ করে দেবার চিন্তা করেছেন? (Has the thought of ending your life been on your mind)	☐ ☐
18	আপনার কি সবসময় হয়রান বা কাহিল লেগেছে গত চার সপ্তাহে? (Do you feel tired all the time?)	☐ ☐
19	আপনার পেটে কোন অসুবিধা বা অস্বস্তি হয়েছে গত চার সপ্তাহে? (Are you easily tired?)	☐ ☐
20	আপনি কি অল্পতেই বা একটুতেই হয়রান বা কাহিল হয়ে গেছেন গত চার সপ্তাহে? (Do you have uncomfortable feelings in you)	☐ ☐

International Centre for Diarrhoeal Disease Research, Bangladesh (icddr,b)
Maternal and Child Health Division (MCHD)
Project title: **Unconditional Lactational Allowance + Pyshosocial Stimulation**
Service delivery questionnaire (Facility)

SL	Name of services (have within three months)	Answer	
1	Iodine supplementation before or during pregnancy	Yes = 1	No = 0
2	Antenatal corticosteroids for women at risk of preterm birth	Yes = 1	No = 0
3	Magnesium sulphate for women at risk of preterm birth	Yes = 1	No = 0
4	Aniplatelet agents for women at risk of pre-eclampsia	Yes = 1	No = 0
5	Therapeutic hypothermia for hypoxic for ischaemic encephalopathy	Yes = 1	No = 0
6	Psychological interventions for common perinatal mental disorders	Yes = 1	No = 0
7	Iron supplementation for children	Yes = 1	No = 0
8	Multiple micronutrient supplementation in children	Yes = 1	No = 0

9	Supplementary feeding for disadvantaged children	Yes = 1	No = 0
10	Parenting programs	Yes = 1	No = 0
11	Integrated parenting and nutrition programs	Yes = 1	No = 0
12	Out-of- home interventions (pre-primary education)	Yes = 1	No = 0
13	Conditional cash transfer	Yes = 1	No = 0
14	Delayed cord camping(ie, more placental transfusion)	Yes = 1	No = 0
15	Breast feeding promotion, education, or support	Yes = 1	No = 0
16	Unconditional cash transfer	Yes = 1	No = 0
17	Periconceptional folic acid fortification or supplementation	Yes = 1	No = 0
18	Birth interval at least 36-60 months	Yes = 1	No = 0
19	Preconceptional diabetes care	Yes = 1	No = 0
20	Iron and Iron folate supplementation during pregnancy	Yes = 1	No = 0
21	Multiple micronutrient supplementation during pregnancy	Yes = 1	No = 0
22	Balanced protein energy supplementation during pregnancy	Yes = 1	No = 0
23	Intermittent preventive therapy and use of bednets for malaria prevention in mothers and children	Yes = 1	No = 0
24	Antibiotics for premature rupture of membranes	Yes = 1	No = 0

25	Lower genital tract infection screening and treatment in pregnant mothers	Yes = 1	No = 0
26	Antibiotics for symptomatic bacteriuria in children	Yes = 1	No = 0
27	Detection and treatment of syphilis in pregnant mothers	Yes = 1	No = 0
28	Smoking cessation interventions in parents	Yes = 1	No = 0
29	Continuous support during childbirth	Yes = 1	No = 0
30	Kangaroo mother care, Skin to skin, Cap and wrap(thermal care)	Yes = 1	No = 0
31	Topical emollient therapy for preterm neonates	Yes = 1	No = 0
32	Intramuscular vitamin for neonates	Yes = 1	No = 0
33	Hand washing behavior and water quality improvement eg, water, sanitation and hygiene(WASH)	Yes = 1	No = 0
34	Rotavirus HiB and pneumococcal vaccinations in children	Yes = 1	No = 0
35	Vitamin A supplementations in children	Yes = 1	No = 0
36	Zinc supplementation and treatment for acute diarrhea in children	Yes = 1	No = 0
37	Deworming drug treatment in children	Yes = 1	No = 0
38	Complementary feeding education and provision	Yes = 1	No = 0
39	Treatment of moderate and severe acute malnutrition in children	Yes = 1	No = 0

40	Interventions to prevent child maltreatment (eg, specific home visiting and parenting programmes)	Yes = 1	No = 0

International Centre for Diarrhoeal Disease Research, Bangladesh (icddr,b)
Maternal and Child Health Division (MCHD)
Project title: **Unconditional Lactational Allowance + Pyshosocial Stimulation**
Tanahsi Framework Analysis Questionnaire (Home based)

Briefly describe to mother: You have a child of 6-16 years old, when you were pregnant and after delivery the child you went to and still now you go to a service centre for the services of antenatal care, health education, early chid development education, parenting programme, nutritional care and others health problems, please tell us which facility you previously used for you and your children.

Name of service centre:

Now would you please answer what services particularly you received from your stated service centre? (interviewer check the bellow list and fill the answer)

Question 1:

		Service Accessibility			
SL	Name of services (have within three months)	How do you go there usually:<br><br>1=on foot<br>2=transport	Name of transport<br><br>1=Bus, auto rickshaw, easy bike, ambulance, motorcycle,<br>2=Boat, engine boat, launch, ferry<br>3=rickshaw, bicycle<br>4= On foot<br>5=Others	Is it easiest way to go there?<br><br>Yes=1<br><br>No=2	How long it take time to go there (minutes)
1	Iodine supplementation before or during pregnancy(Antenatal care)				
2	Antenatal corticosteroids for women at risk of preterm birth (Antenatal care)				
3	Magnesium sulphate for women at risk of preterm birth(Antenatal care)				
4	Aniplatelet agents for women at risk of pre-eclampsia (Antenatal care)				
5	Therapeutic hypothermia for hypoxic for ischaemic encephalopathy				

6	Psychological interventions for common perinatal mental disorders(Antenatal care)				
7	Iron supplementation for children				
8	Multiple micronutrient supplementation in children				
9	Supplementary feeding for disadvantaged children				
10	Parenting programs				
11	Integrated parenting and nutrition programs				
12	Out-of- home interventions (pre-primary education)				
13	Conditional cash transfer				
14	Delayed cord camping(ie, more placental transfusion)				
15	Breast feeding promotion, education, or support				
16	Unconditional cash transfer				
17	Periconceptional folic acid fortification or supplementation(Antenatal care)				
18	Birth interval at least 36-60 months				
19	Preconceptional diabetes care				
20	Iron and Iron folate supplementation during pregnancy (Antenatal care)				
21	Multiple micronutrient supplementation during pregnancy(Antenatal care)				
22	Balanced protein energy supplementation during pregnancy(Antenatal care)				
23	Intermittent preventive therapy and use of bednets for malaria prevention in mothers and children(Antenatal + postnatal care)				
24	Antibiotics for premature rupture of membranes (Antenatal care)				
25	Lower genital tract infection screening and treatment in pregnant mothers(Antenatal care)				

26	Antibiotics for symptomatic bacteriuria in children				
27	Detection and treatment of syphilis in pregnant mothers				
28	Smoking cessation interventions in parents				
29	Continuous support during childbirth				
30	Kangaroo mother care, Skin to skin, Cap and wrap(thermal care)				
31	Topical emollient therapy for preterm neonates				
32	Intramuscular vitamin for neonates				
33	Hand washing behavior and water quality improvement eg, water, sanitation and hygiene(WASH)				
34	Rotavirus HiB and pneumococcal vaccinations in children				
35	Vitamin A supplementations in children				
36	Zinc supplementation and treatment for acute diarrhea in children				
37	Deworming drug treatment in children				
38	Complementary feeding education and provision				
39	Treatment of moderate and severe acute malnutrition in children				
40	Interventions to prevent child maltreatment (eg, specific homevisiting and parenting programmes)				

International Centre for Diarrhoeal Disease Research, Bangladesh (icddr,b)
Maternal and Child Health Division (MCHD)
Indirect Cost measurement Questionnaire(Parental Time)

Child's Name:	Child's ID: / _ / _ / _ / _ / _ / _ / _ / _ / _ /
Interviewer's Name:	DOT (DD/MM/YY) ☐☐/☐☐/☐☐
Mother's name:	Father's Name:
Mother's Income:	Father's Income:
Mother's Age:	Father's Age:
Village Name:	Union Name:<br>Ward#

আপনারা আপনাদের শিশুর সাথে বই পড়া, ছবির বই দেখানো, গান শোনানো, বা খেলনা দিয়ে গত তিন দিনে কত সময় খেলেছেন বা কত সময় কাটিয়েছেন, সে সম্পর্কে জানতে এসেছি ।

SL	Item no	Who did this with index child in past three days?		Day	Minute spend	Total
1.	বাচ্চাকে বই পড়ে শুনিয়েছেন,ছবির বই /ছবি / পোষ্টার দেখিয়েছেন- (Book was read or picture book/picture/poster was shown to your child by-	Mother		1st day		
				2nd day		
				3rd day		
		Father		1st day		
				2nd day		
				3rd day		
		Others, (Relation with child above 18yrs)		1st day		
		Age	2nd day	2nd day		
		Sex	3rd day	3rd day		
2.	বাচ্চাকে (নাম) গল্প বলেছেন- (Story was told to the child (name) by-	Mother		1st day		
				2nd day		
				3rd day		
		Father		1st day		
				2nd day		
				3rd day		
		Others, (Relation with child above 18yrs)		1st day		
		Age	Education	2nd day		
		Sex	Monthly income	3rd day		
3.	গান / ছড়া গান,গজল গেয়েছেন - (Song, rhyme, religious song was sung to the child (name) by-	Mother		1st day		
				2nd day		
				3rd day		
		Father		1st day		
				2nd day		
				3rd day		
		Others, (Relation with child above 18yrs)		1st day		
		Age	Education	2nd day		
		Sex	Monthly income	3rd day		

4.	বাচ্চার (নাম) সাথে খেলনা দিয়ে খেলেছেন (Game was played with the child (name) using toys by-	Mother		1st day		
				2nd day		
				3rd day		
		Father		1st day		
				2nd day		
				3rd day		
		Others, (Relation with child above 18yrs)		1st day		
		Age	Education	2nd day		
		Sex	Monthly income	3rd day		
5.	বাচ্চাকে (নাম) সময় দিয়ে কোন কিছুর নাম, গননা এবং আঁকাআকি শিখিয়েছেন- *(Name of* something, counting number & drawing was taught to the child (name) by allocating time for him by-	Mother		1st day		
				2nd day		
				3rd day		
		Father		1st day		
				2nd day		
				3rd day		
		Others,(Relation with child above 18yrs)		1st day		
		Age	Education	2nd day		
		Sex	Monthly income	3rd day		

Bayley™
Scales of Infant and Toddler Development™
THIRD EDITION

Record Form

Child's name: ____________________

Sex: ☐ M ☐ F ID #: ____________________

Examiner's name: ____________________

School/Child care program: ____________________

Reason for referral: ____________________

Subtest Summary Scores

Subtest	Total Raw Score	Scaled Score	Composite Score	Percentile Rank	Conf. Interval (__%)
**Cognitive** (Cog)					
			Use Table A.5		
**Language** (Lang)					
Receptive Communication (RC)					
Expressive Communication (EC)					
**Sum**					
			Use Table A.4		
**Motor** (Mot)					
Fine Motor (FM)					
Gross Motor (GM)					
**Sum**					
			Use Table A.4		
**Social-Emotional** (SE)					
			Use Table A.5		
**Adaptive Behavior**					
*Communication (Com)					
Community Use (CU)					
Functional Pre-Academics (FA)					
Home Living (HL)					
*Health and Safety (HS)					
*Leisure (LS)					
*Self-Care (SC)					
*Self-Direction (SD)					
*Social (Soc)					
*Motor (MO)			(GAC)		
**Sum**					
			Use Table A.6		

*For children younger than one year, the GAC is calculated using only those skill areas indicated by an asterisk.

Calculate Age and Start Point

	Years	Months	Days
Date Tested			
Date of Birth			
Age			
Age in Months and Days	Years × 12 + months		
Adjustment for Prematurity	Adjust through 24 months		
Adjusted Age			
Start Point	Calculate start point according to chart below		

Age	Start Point
16 days–1 month 15 days	A
1 month 16 days–2 months 15 days	B
2 months 16 days–3 months 15 days	C
3 months 16 days–4 months 15 days	D
4 months 16 days–5 months 15 days	E
5 months 16 days–6 months 15 days	F
6 months 16 days–8 months 30 days	G
9 months 0 days–10 months 30 days	H
11 months 0 days–13 months 15 days	I
13 months 16 days–16 months 15 days	J
16 months 16 days–19 months 15 days	K
19 months 16 days–22 months 15 days	L
22 months 16 days–25 months 15 days	M
25 months 16 days–28 months 15 days	N
28 months 16 days–32 months 30 days	O
33 months 0 days–38 months 30 days	P
39 months 0 days–42 months 15 days	Q

PEARSON

Copyright © 2006, 1993, 1984, 1969 by NCS Pearson, Inc.
All rights reserved. Printed in the United States of America.

8 9 10 11 12 A B C D E

PsychCorp

ISBN 015402723-5
9 780154 027238

Template for Budget Version: 1 Date: 27/12/2011

Detailed Budget for the study titled: _Urban_LA_PS__

Name of Principal Investigator: Sheikh Jamal Hossain
Protocol Number: PR-18035
Division: MCHD
Funding Source: GCC
Budget: Direct: US$225107; Indirect: US$24892; Total: US$250000
Study period: From : May 2018 through April 2020
Strategic Priority Code(s):

**Line Items**	**Budget**										
Payroll and Benefits:	Name of personnel/position	Pay level	% Effort	# of posts	Monthly Rate	Year-1	Year-2	Year-3	Year-4	Year-5	Total amount (US$)
	PI (Jamal)	NOB/2	40%			11,442	16,432				27,874
	Co-PI-Dr Jena	P4/3	5%			8,083	9,317				17,400
	Co-I -Dr Fahmida	NOD/5	5%			2,675	1,605				4,279
	Co_I_Dr. Muhith	NOB/2	20%			1,560					1,560
	Consultant					-	3,000				3,000
	Finance Manager	NOA/2	25%			4,725	5,199				9,924
	**Sub-total of Payroll and benefits:**										196,009
Travel and transport	Travel costs of testers/mothers for baseline										1,980
	Travel costs of testers/mothers for endline										1,980
	Travel and related cost (Govt. Personnel, PI, CoPI, project personnel & others stake holders)										5,800
	PI_Int. Travel(ticket, perdium & others)										7,000
	Travel costs of testers/mothers for baseline										1,980

	**Sub-total of Travel and Transport:**						16,760
Supply and materials	Bayley record form & others						7,260
	Bayley Permission Fee and related cost						1,500
	Stimulation materials & related cost						660
	Supplies item and Others						1,999
	Meeting & workshop, dissemination and (all related cost)						2,500
	**Sub-total of supply and materials:**						13,919
Other contractual							
	**Sub-total of other contractual:**						
**Total direct costs:**							225,107
**Total indirect cost:**							24,892
**Total costs:**							**250000**

ANNEX-2

Gender analysis of development and growth

Gender tools	Are there sex differences in	How do biological differences between men and women influence their:	How do the environmental factors affects	How do gender norms/ values affect men and women's	How do access to control over resources affects
**In relation to growth and development**					
**Vulnerability: (Male/female)**	Children having less growth and development for both male and female children are vulnerable to coping with all socioeconomic factors.	Some biological factors while female children mature, such as premenstrual problems, pregnancy, infertility**,** perimenopause and menopause leads women more vulnerable to growth and development**.**	Female experience more negative events and have less control over important areas of their life than men.	Both male and female faces problems to norms and values.	[May not be applicable]
**Health seeking behavior**	Female **usually do not seek health care due to shame (social stigma) compare to their counter part boys.**	May not be applicable	Due to environmental factors girls do not go out of home alone. So they lack health care sometimes.	Social norms is girls should not move alone.	
**Ability to access health services**	Female have less access to health services than boys.		Most of the time male go outside alone which increase their access to health service than girls.		Female have less freedom and access to health services than boys
**Preventive and treatment option, responses to treatment**	Female may be more responsive for development for their simplicity to preventive program.	Female may have more responsive to developmental intervention.	Positive and supportive environment can improve treatment response among girls	Social stigma of lack of developmen t can interrupt management among girls	Not applicable
**Experiences with health services and health providers**	Not applicable	Not applicable	Not applicable		Not applicable

Check-List

Check-list for Submission of Research Protocol For Consideration of the Research Review Committee (RRC) [Please check all appropriate boxes]

1. Has the proposal been reviewed, discussed and cleared by all listed investigators?<br><br>☒ Yes ☐ No<br><br>If the response is No, please clarify the reasons:
2. Has the proposal been peer-reviewed externally?<br><br>☐ Yes ☐ No ☒ External Review Exempted<br><br>If the response is 'No' or "External Review Exempted", please explain the reasons: It is a competitive fund which ultimately was a result of peer review.<br><br>If the response is "Yes", please indicate if all of their comments have been addressed?<br>☐ Yes (please attach)<br><br>☐ No (please indicate reason(s)):
3. Has the budget been reviewed and approved by icddr,b's Finance?<br>☒ Yes ☐ No (reason):<br>____
4. Has the Ethics Certificate(s) been attached with the Protocol?<br><br>☒ Yes ☐ No<br>If the answer is 'No', please explain the reasons:
_______________________________________ ___________<br>Signature of the Principal Investigator Date

Guidelines for Preparing Abstract for ERC

The Ethical Review Committee will not consider any application that does not include an abstract summary. The abstract should summarise the purpose of the study, the methods and procedures to be used, by addressing each of the following items. If an item is not applicable, please note accordingly, describing the reason:

9. Describe the requirements for a 'study population' and explain the rationale for inclusion of special groups in this study population, such as children or groups whose ability to give voluntary informed consents might be compromised.

10. Assess and describe potential risk(s) – physical, psychological, social, legal or other, and also assess their likelihood and seriousness. If research methods are anticipated to involve potential risks, describe alternate methods, if any, which were considered and why they will not be used.

11. Describe procedures for protecting against or minimising potential risks, and an assessment of their likely effectiveness.

12. Include a description of the methods for safeguarding confidentiality and protecting anonymity.

13. When there are potential risks to the participants, or when the privacy of the individual may be affected, the investigators are required to obtain a written informed consent, duly signed by the prospective participants. For minors and individuals with compromised ability to provide a valid consent, informed consent must be obtained from their parents or legal guardians. Describe consent procedures to be followed including how and where informed consent will be obtained.

    d) If signed consent will not be obtained, explain why this requirement should be waived and provide an alternative procedure that would be used.

    e) If information is to be withheld from a participant, provide justification for this course of action.

    f) If there is a potential risk to the participant or privacy of the individual might be affected while applying any particular procedure include a statement in the consent form to clarify whether or not compensation and/or treatment will be available and who will support the costs.

14. If study involves an interview, describe the place and processes, and approximate length of the interview.

15. Assess the potential benefits to be gained or risk the individual participants might be subjected to, and also the benefits that might accrue to the society in general as a result of the planned work. Clarify if and how the benefits outweigh the risks.

16. State if the activity requires the use of records (hospital, medical, birth, death or other), organs, tissues, body fluids, the foetus or the abortus.

The statement to the potential participants should include information specified in item 2,3,4,5(c) and 7, and also indicate the approximate time they would be required to remain in the activity.

References:

1. Black MM, Walker SP, Fernald LC, Andersen CT, DiGirolamo AM, Lu C, et al. Early childhood development coming of age: science through the life course. The Lancet. 2017;389(10064):77-90.
2. Hamadani JD, Tofail F, Huda SN, Alam DS, Ridout DA, Attanasio O, et al. Cognitive deficit and poverty in the first 5 years of childhood in Bangladesh. Pediatrics. 2014;134(4):e1001-e8.
3. Bangladesh Bureau of Statistics (BBS) SaID, Ministry of planning. Population Projection of Bangladesh:Dynamics and Trends 2011-2061. 2015.
4. Ellis P, Roberts M. Leveraging urbanization in South Asia: Managing spatial transformation for prosperity and livability: World Bank Publications; 2015.
5. Afsana K, Wahid SS. Health care for poor people in the urban slums of Bangladesh. The Lancet. 2013;382(9910):2049-51.
6. Adato M, Hoddinott J. Conditional cash transfer programs: A" magic bullet" for reducing poverty? 2007.
7. Fiszbein A, Schady NR. Conditional cash transfers: reducing present and future poverty: World Bank Publications; 2009.
8. Rawlings LB, Rubio GM. Evaluating the impact of conditional cash transfer programs. The World Bank Research Observer. 2005;20(1):29-55.
9. Fernald LC, Gertler PJ, Neufeld LM. Role of cash in conditional cash transfer programmes for child health, growth, and development: an analysis of Mexico's Oportunidades. The Lancet. 2008;371(9615):828-37.
10. Levere M, Acharya G, Bharadwaj P. The role of information and cash transfers on early childhood development: evidence from Nepal. National Bureau of Economic Research, 2016.
11. Robertson L, Mushati P, Eaton JW, Dumba L, Mavise G, Makoni J, et al. Effects of unconditional and conditional cash transfers on child health and development in Zimbabwe: a cluster-randomised trial. The Lancet. 2013;381(9874):1283-92.
12. Pega F, Liu SY, Walter S, Pabayo R, Saith R, Lhachimi SK. Unconditional cash transfers for reducing poverty and vulnerabilities: effect on use of health services and health outcomes in low-and middle-income countries. The Cochrane Library. 2017.
13. Hamadani JD, Huda SN, Khatun F, Grantham-McGregor SM. Psychosocial stimulation improves the development of undernourished children in rural Bangladesh. The Journal of nutrition. 2006;136(10):2645-52.
14. Ahmed SM, Evans TG, Standing H, Mahmud S. Harnessing pluralism for better health in Bangladesh. The Lancet. 2013;382(9906):1746-55.
15. Ministry of Women and Children Affairs GoB. the Children Policy 2011. 2011.
16. Britto PR, Lye SJ, Proulx K, Yousafzai AK, Matthews SG, Vaivada T, et al. Nurturing care: promoting early childhood development. The Lancet. 2017;389(10064):91-102.

17. Hamadani JD, Huda SN, Khatun F, Grantham-McGregor SM. Psychosocial stimulation improves the development of undernourished children in rural Bangladesh. J Nutr. 2006;136(10):2645-52.
18. Nahar B, Hamadani J, Ahmed T, Tofail F, Rahman A, Huda S, et al. Effects of psychosocial stimulation on growth and development of severely malnourished children in a nutrition unit in Bangladesh. European Journal of Clinical Nutrition. 2009;63(6):725-31.
19. Nahar B, Hossain MI, Hamadani JD, Ahmed T, Huda SN, Grantham-McGregor SM, et al. Effects of a community-based approach of food and psychosocial stimulation on growth and development of severely malnourished children in Bangladesh: a randomised trial. Eur J Clin Nutr. 2012;66(6):701-9.
20. Tofail F, Hamadani JD, Mehrin F, Ridout DA, Huda SN, Grantham-McGregor SM. Psychosocial stimulation benefits development in nonanemic children but not in anemic, iron-deficient children. J Nutr. 2013;143(6):885-93.
